# Transposase subunit architecture and its relationship to genome size and the rate of transposition in prokaryotes and eukaryotes

**DOI:** 10.1093/nar/gky794

**Published:** 2018-09-04

**Authors:** George Blundell-Hunter, Michael Tellier, Ronald Chalmers

**Affiliations:** School of Life Sciences, University of Nottingham, Queen’s Medical Centre, Nottingham NG7 2UH, UK

## Abstract

Cut-and-paste transposons are important tools for mutagenesis, gene-delivery and DNA sequencing applications. At the molecular level, the most thoroughly understood are Tn*5* and Tn*10* in bacteria, and mariner and hAT elements in eukaryotes. All bacterial cut-and-paste transposases characterized to date are monomeric prior to interacting with the transposon end, while all eukaryotic transposases are multimers. Although there is a limited sample size, we proposed that this defines two pathways for transpososome assembly which distinguishes the mechanism of the bacterial and eukaryotic transposons. We predicted that the respective pathways would dictate how the rate of transposition is related to transposase concentration and genome size. Here, we have tested these predictions by creating a single-chain dimer version of the bacterial Tn*5* transposase. We show that artificial dimerization switches the transpososome assembly pathway from the bacterial-style to the eukaryotic-style. Although this had no effect *in vitro*, where the transposase does not have to search far to locate the transposon ends, it increased the rate of transposition in bacterial and HeLa cell assays. However, in contrast to the mariner elements, the Tn*5* single-chain dimer remained unaffected by over-production inhibition, which is an emergent property of the transposase subunit structure in the mariner elements.

## INTRODUCTION

DNA transposons are widely distributed in nature and are an important source of variation (1). Most elements encode only one, or sometimes two, proteins that are required for transposition. Bioinformatic and biochemical analyses revealed that there are several families of transposons with distinctly different molecular mechanisms for mobilization and amplification. Some of the families, and modes of transposition, have a restricted phylogenetic distribution, while others are almost universal. The most widely distributed elements are probably those in which the transposase has an RNaseH-like structural-fold with a DDE(D) triad of amino acids in the active site. Of these, the cut-and-paste elements are the most successful, and include many examples such as Tn*10*, Tn*5* and members of the IS*630*-Tc*1*-mariner (ITm) superfamily (2).

Transposon mutagenesis is a widely used tool in genetics and biochemistry. Sleeping Beauty and piggyBac are the most efficient in mammalian cells, while Tn*10*, Tn*5* and phage Mu are often used in bacteria (3,4,5,6). *In vitro* reconstituted versions of Tn*5* and Mu are particularly useful because the transpososomes can be assembled from purified components and used to mutagenize DNA *in vitro* or delivered into cells by transformation or electroporation (7,8,9). When used *in vitro*, these systems are almost 100% efficient and their applications include the fragmentation of DNA and the delivery of sequence tags for deep sequencing library construction. However, attempts at integration in mammalian cells have provided patchy results. Microinjection, electroporation and particle bombardment of Tn*5* and bacteriophage Mu transpososomes have been used to integrate DNA into the genomes of mammalian cells, rice plants, yeast and trypanosomes (10,11,12,13). However, the two-plasmid transfection-assay, widely used for Sleeping Beauty and piggyBac transgenesis, has not been reported for Tn*5*. In this type of assay, the reporter-transposon is on one plasmid, while the transposase expression cassette is on another (14).

Before the transposon can be excised from the donor site, the transposase must first bind both ends of the element and bring them together in a paired ends complex (PEC), also sometimes called a synaptic complex or transpososome. In Tn*5* the complex contains two monomers, which each cleave both strands of DNA at the respective ends of the transposon (15,16). The requirement for synapsis before catalysis is presumably an adaptive feature that protects the transposon and the host from the DNA damage that might arise from a partial-reaction at one end of the element. Assembly of the transpososome is also a key checkpoint: all known regulatory-mechanisms operate before this stage of the reaction, and after the complex has formed the transposon is committed to transposition.

Genome size is an important factor that affects the rate of transposition in eukaryotic cells were transcription and translation are uncoupled (i.e. take place in different compartments). If a transposon has a broad phylogenetic distribution, the transposase must be able to search for and find the transposon ends in genomes that range in size over five orders of magnitude (∼10^6^ - 10^11^ bp). In the bacterial elements, such as Tn*10*, Tn*5* and IS*911*, for example, this difficulty is largely circumvented by a *cis*-acting transposase; in other words each transposon sees only the transposase transcribed and translated from its own transposase gene (17,18,19). There are several mechanisms for *cis*-action but they all require coupled transcription and translation, which ensures that the transposase is synthesized close to the transposon. This means that the apparent transposase-concentration is largely independent of the genome size and cellular volume. However, in eukaryotes the transposase must search for transposon ends after entering the nucleus.

**Figure 1.**
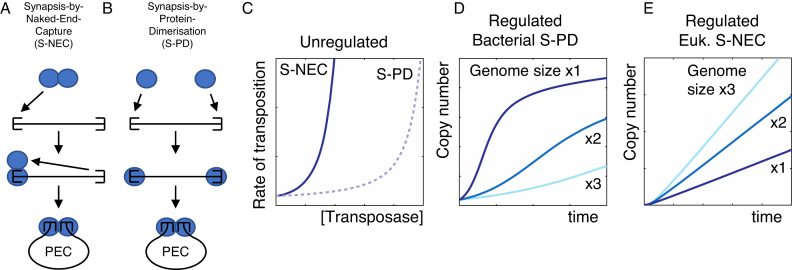
Two pathways for transpososome assembly. (**A** and **B**) The two pathways for transpososome assembly are illustrated: blue spheres are transposase subunits; half-rectangles are transposon ends. The flanking DNA is omitted for clarity. In the S-NEC pathway a transposase dimer binds to the transposon end and recruits a second, unbound, end into the developing transpososome. In the S-PD pathway the transposase subunits bind transposon ends independently and the ends are brought together by protein dimerization. In Tn*10*, the archetypal bacterial cut-and-paste transposon, and the closely related Tn*5*, the synaptic complex is very stable and its assembly is essentially irreversible. In the crystal structure of the Tn*5* post-cleavage intermediate two thirds of the interface between the two halves of the complex is provided by protein–DNA interactions (40). Tight binding of the transposase and the transposon ends is therefore established only upon synapsis. PEC, paired-ends complex also sometimes called the transpososome or synaptic complex. (**C–E**) Computer simulations of a transposon genomic-invasion were performed as in (22). In part D the simulation includes a *cis*-acting transposase and a *trans*-acting inhibitor. In part E regulation is an emergent property of the S-NEC pathway and no additional components are required.

Previously, we investigated this problem in a mariner transposon using a combination of biochemical analysis and computer simulations (20,21,22,23,24,25). Biochemical analysis had shown that many bacterial DDE(D) transposase are monomers and multimerize only after interacting with the transposon ends (e.g. (26,27,28,29)). In contrast, those eukaryotic members of the superfamily examined to date are multimers in solution (e.g. (30,31,32,33,34,35)). Our hypothesis was that the multimeric state defined two pathways for synaptic-complex assembly (Figure 1A and B). In eukaryotes, the transposase multimer would first bind one end and then recruit a second unbound end. We term this synapsis-by-naked-end-capture (S-NEC) (22). In many bacterial elements, the monomeric transposase interacts independently with the transposon ends and the transpososome is assembled by a mechanism that we term synapsis by protein dimerization (S-PD) (22). This is intrinsically less efficient because two transposase monomers and two transposon ends must interact simultaneously (Figure 1B). A computer simulation demonstrated that an S-NEC reaction is faster than S-PD at any given transposase concentration (Figure 1C). We went on to incorporate autoregulation into the simulations to get insight into the ways in which the exponential amplification of transposons can be suppressed (22). One of the insights was the way in which the rate of transposition responds to genome size (Figure 1D and E). In an S-PD reaction the rate decreases with genome size (Figure 1D). In a eukaryotic system, in the absence of *cis*-action, this is expected because transposase is sequestered in non-specific DNA-interactions and takes longer to locate the transposon ends and assemble the transpososome. This response might also be expected in an S-NEC reaction. However, the simulation yielded a counter-intuitive result: above a certain transposase concentration, the rate of transposition increases with genome size (Figure 1E). This is because non-specific interactions relieve auto-inhibition of the transposase (22).

The veracity of the S-NEC simulations was supported by the dose-response curves for the Hsmar1, piggyBac and Sleeping Beauty transposases in HeLa cells (22). To extend our experimental tests of the hypothesis and the simulations, we wished to manipulate the subunit architecture of the transposases. We do not know of any reports of mutations that disrupt the subunit interface of a natural transposase multimer without at the same time inhibiting the activity e.g. (36,37). However, we wondered whether we could engineer a dimeric version of a monomeric bacterial transposase and what effect this would have on the reaction.

## MATERIALS AND METHODS

### Plasmid construction

Plasmid sequences are provided in Supplementary Table S1. For protein expression and purification, the hyperactive Tn5 transposase gene described by (28) was cloned in pTYB2 (NEB) between the NdeI and XmaI sites to yield pRC2128. The single-chain dimeric transposase was constructed by joining two copies of the transposase gene with a 22 amino acid linker (GPSRGGGSEGGGSEGGSGTSQL) between the C-terminus of the first monomer and N-terminus of the second to yield pRC2102. Site directed mutagenesis was used to introduce point mutations into the respective subunits of the single-chain dimer. The active site DDE to ADE mutations in the N- and C-terminal subunits of the single-chain dimer were expressed from pRC2143 and pRC2107, respectively. The G462D dimerization mutations in the N- and C-terminal subunits of the single-chain dimer were expressed from pRC2152 and pRC2153, respectively. The reporter plasmid for the *in vitro* transposition reactions was pRC2106. This contained the Tn5 mosaic ends (28) flanking a Neomycin resistance gene.

The papillation reporter transposons for Hsmar1 was encoded on plasmid pRC681. This constructed prior to integration into the genome at the *argE* locus by lambda Red recombination (see below). The Hsmar1 reporter had a pair of transposon ends flanking a promoterless *lacZ* and a kanamycin resistance gene (25,38). The Tn*5* reporter was generated by polymerase chain reaction (PCR), which was used to add *argE* homology arms and the Tn*5* mosaic ends (28) to the lacZ-kan fragment from pRC681. For the papillation assay with ampicillin the monomeric and single-chain dimeric Tn*5* transposases were expressed from pRC2117 and pRC2109, respectively, which are derived from pMAL-C2X (NEB). The transposases were inserted between the NdeI and SbfI sites. This removes the maltose binding protein gene and leaves the transposase under the control of the *ptac* promoter. The Chloramphenicol resistant plasmids of the monomeric and dimeric Tn5 transposase were pRC2114 and pRC2113, respectively, which are identical to pRC2117 and pRC2109 except for the addition of a chloramphenicol resistance cassette at the SwaI site. The Hsmar1 transposase was expressed from pRC1721, which has the bacterial codon optimized transposase gene cloned between the NdeI and XbaI sites of pMAL-C2X (NEB). As before, this removes the maltose binding protein gene and leaves the transposase under the control of the *ptac* promoter.

For the HeLa cell assays, Tn*5* transposase was codon optimized for expression in human cells and cloned into pcDNA3.1 with an SV40 nuclear localization signal (NLS) added to the N-terminus and a poly-histidine tag on the C-terminus. The monomeric and single-chain dimer versions were named pRC2148 and pRC2147, respectively. The GFP control was constructed by PCR amplification of sfGFP, and cloned it into pQE trisystem His-Strep 1 (Qiagen) at the NcoI site and named pRC2116. For the GFP-transposase localization experiments, sfGFP was PCR amplified and inserted into pRC2148 or pRC2147 at the HpaI site (located between the SV40 NLS and the transposase), in order to make pRC2164 and pRC2163, respectively. The transposon reporter plasmid (pRC2194) encoded a neomycin resistance marker, driven by EM7 and PGK promoters, flanked by a pair of Tn*5* mosaic ends (28) in an inverted configuration. The transposon was 2589 bp long.

### *Escherichia coli* strain construction

Papillation reporter strains were derived from RC5095, which is identical to ER1793 (NEB): *Escherichia coli F- fhuA2 Δ(lacZ)r1 glnV44 e14-(McrA-) trp-31 his-1 rpsL104 xyl-7 mtl-2 metB1 Δ(mcrC-mrr)114::IS10*. The Hsmar1 and Tn*5* reporter strains were created by using PCR to amplify the reporter transposons with homology arms for lambda red recombineering into the *ArgE* locus. The Hsmar1 reporter was amplified directly from pRC681, which encodes a lacZ'-Kan fragment flanked by a pair of transposon ends. The sequences of the homology-arms at either end of the reporters were: 5′-cggatgcggcgcgagcgccttatccggcctacgttttaatgccagca and 5′-gcttcaagaaactgcggcgctaaatcgtggctaacttcatgggcggt. The Tn*5* reporter was amplified from the same template except that the Hsmar1 transposon ends were replaced by a pair of Tn*5* mosaic ends.

### Tn*5* transposase expression and purification

The Tn*5* transposase expression plasmids were transformed into ER2566 (NEB: *F^−^ fhuA2 [lon] ompT lacZ::T7 genel gal sulA11 delta(mcrC-mrr)114::IS10R(mcr-73::miniTn10)2 R(zgb-210::Tn10)1 (Tetsensitive) endA1 [dcm]*). A colony was grown in 20 ml LB supplemented with 100 μg/ml ampicillin at 37°C for 16 h, before being used to inoculate 2 l of LB supplemented with 100 μg/ml ampicillin. Cells were grown at 37°C to OD_600_ = 0.4, whereupon it was made 0.5 mM in isopropyl β-D-1-thiogalactopyranoside (IPTG) and the temperature adjusted to 23°C for 4 h. The cells were then centrifuged, 5000 × *g* at 4°C for 20 min. The cell pellet was resuspended in 40 ml column buffer (20 mM Tris pH 7.5, 700 mM NaCl, 0.1% Triton X-100, 10% glycerol) and frozen at −80°C. The cells were thawed and a protease inhibitor cocktail tablet (Roche Applied Science) was added. The cells were lysed using a French Pressure cell (American Instrument Co., Inc., Silver Spring, MD, USA) at 10 000 Psi. The lysate was centrifuged at 12 000 × *g* at 4°C for 1 h. The supernatant was loaded onto a column containing 2 ml of chitin resin (NEB). The resin was washed with 30 ml column buffer, followed by 6 ml cleavage buffer (column buffer + 50 mM dithiothreitol [DTT]). The column was then capped and loaded with 1 ml cleavage buffer, before being stored for 16 h at 4°C. Ten 0.5 ml aliquots of the eluate were collected and analyzed on a 10% sodium dodecyl sulphate-polyacrylamide gel electrophoresis (SDS-PAGE) gel. Transposase was quantified through a Bradford assay. The samples were then aliquoted and frozen at −80°C.

### *In vitro* transposition assay

The transposition reaction buffer contained 100 mM potassium glutamate, 0.5 μg/ml bovine serum albumin (BSA), 25 mM Tris.HCl pH 7.5, 0.5 μM β-mercaptoethanol, 10 mM MgCl_2_. Reactions (20 μl) contained 400 ng (7 nM) of pRC2106, which encodes two 19 bp Tn*5* mosaic ends (28) flanking a kanamycin resistance gene. The transposase was added last and was 100 nM unless stated otherwise. Reactions were incubated at 37°C for 4 h unless stated otherwise, and were terminated by addition of SDS to 0.2% SDS and heating to 68°C for 5 min. Reactions were analyzed on a 1% agarose Tris/Borate/Ethylenediaminetetraacetic acid (TBE) buffered gel, which was electrophoresed overnight at 2.3 V/cm. The gel was then stained with ethidium bromide and photographed.

### Electrophoretic mobility shift assay (EMSA)

The electrophoretic mobility shift assay (EMSA) was performed essentially as described previously (25). The binding buffer contained 100 mM potassium glutamate, 0.5 μg/ml BSA, 25 mM Tris.HCl pH 7.5, 0.5 μM β-mercaptoethanol. Each reaction (20 μl) contained 2 nM transposon end fragment and 100 nM transposase. DNA fragments encoding the Tn*5* mosaic end were generated by digesting pRC916 with BamHI and XbaI (93 bp), or NotI and KpnI (154 bp) (39). Fragments were end-labeled with α-^32^P dCTP and the Klenow enzyme, purified on an 8% TBE polyacrylamide gel and recovered by the crush and soak method. Transpososome complexes were assembled for 15 min at room temperature before addition of 4.5 μl 80% glycerol to aid gel loading on an 8% TBE polyacrylamide gel, which was electrophoresed at 120 V and imaged using a Fuji FLA-3000 phosphorimager. To analyze cleavage, the reactions were supplemented with 10 mM MgCl_2_ and incubated for a further 2 h at 37°C.

### Papillation assay

The papillation reporter strains for Tn*5* and Hsmar1 were *E. coli* RC5123 and RC5096, respectively. Cells were transformed with 1 ng of transposase expression plasmid. Cells were heat shocked at 42°C, supplemented with 1 ml of LB and incubated at 37°C for an hour. For those expression plasmids that encode ampicillin resistance, the antibiotic was added to a final concentration of 100 μg/ml, after which the cells were incubated for a further 4 h at 37°C. Cells were then serially diluted to 10^−4^-fold with LB and 100 μl was spread on LB agar plates containing 100 μg/ml ampicillin, 0.1% lactose, 40 μg/ml X-gal and galactose and/or IPTG where indicated. For those expression plasmids that encoded resistance to chloramphenicol, the heat shock and 1 h incubation was the same. After the 1 h incubation 100 μl of cells was spread on the LB agar plates containing 25 μg/ml chloramphenicol 0.1% lactose, 40 μg/ml X-gal and galactose and/or IPTG where indicated.

### Western blotting

Cells were lysed using RIPA buffer (50 mM Tris pH 7.5, 150 mM NaCl, 1 mM Na2-EDTA, 1% Triton X-100, 0.5% sodium deoxycholate and 0.1% SDS) and 40 μl protease inhibitor cocktail (Roche Applied Science, one tablet dissolved in 1 ml water). Samples containing 30 μg of protein were mixed with 4× SDS loading buffer (200 mM Tris.HCl pH 6.8, 400 mM DTT, 8% SDS, 0.4% bromophenol blue, 40% glycerol) and incubated in a boiling water bath for 5 min before being loaded on 10% SDS-PAGE gels. After electrophoresis the gel electroblotted to polyvinylidene fluoride (PVDF) membrane. The membrane was incubated in TBST (50 mM Tris, pH 7.6, 150 mM NaCl, 0.1% Tween^®^ 20) + 5% nonfat dry milk for at least 1 h at 4°C. The membrane was incubated with 1 ml of primary antibody (Serotec anti-6× histidine tag mouse monoclonal) diluted 1000-fold in TBSTM (TBST + 5% nonfat dry milk) for 1 h at 4°C. It was then washed three times with TBSTM and incubated with 1 ml secondary antibody (Dako polyclonal HPR-conjugated rabbit anti-mouse diluted 2000-fold in TBSTM). The membrane was then developed using the Promega ECL Chemiluminescence kit and imaged using the Fujifilm LAS-3000 imaging system.

### HeLa cell transfection and integration assay

Cells were seeded into 6-well plates (9 cm^2^ per well) in Dulbecco’s modified Eagle’s medium (DMEM) buffer (Sigma-Aldrich) + 10% FBS (fetal bovine serum), penicillin (100 units per ml) and streptomycin (100 μg/ml). The cells were grown to 70–80% confluence at 37°C in 5% CO_2_ atmosphere. Cells were then drained and provided with 3 ml of DMEM media + 10% FBS without penicillin or streptomycin. Cells were transfected with up to 2 μg of plasmid DNA using Lipofectamine 2000 (Invitrogen), according to the manufacturer's instructions.

For the transposon integration assay, HeLa cells were transfected with 1 μg of a transposon reporter plasmid (pRC2194) plus 1 μg of the transposase expression plasmid (pRC2147 or pRC2148). After 24 h the cells were resuspended by trypsin/ethylenediaminetetraacetic acid (EDTA) treatment and counted using a Hawksley-Neubauer chamber. Plates (100 mm^2^) were seeded with 4 × 10^5^ cells in 10 ml media supplemented with 800 μg/ml G418. This media was changed every 3 days for 2 weeks to allow selection for neomycin resistance. The cells were then fixed to the plates by treating with 10 ml of 10% formaldehyde in PBS, for 15 min. Cell foci were then stained with 10 ml of 1% methylene blue in 70% ethanol for 30 min. Plates were then washed with water, air-dried and photographed. For analysis of transposon insertion sites, genomic DNA was collected from all the remaining foci in the assay and insertions were mapped using splinkerette or inverse PCR mapping.

### Fluorescence microscopy

The cells were transfected with 1 μg of GFP-transposase expression plasmid as described above. After 24 h, the live cells were imaged, in the 6-well plate, using a Carl Zeiss Axiovert S100 TV Inverted Microscope with an HBO 100 illuminator.

## RESULTS

### A Tn*5* transposase single-chain dimer

**Figure 2.**
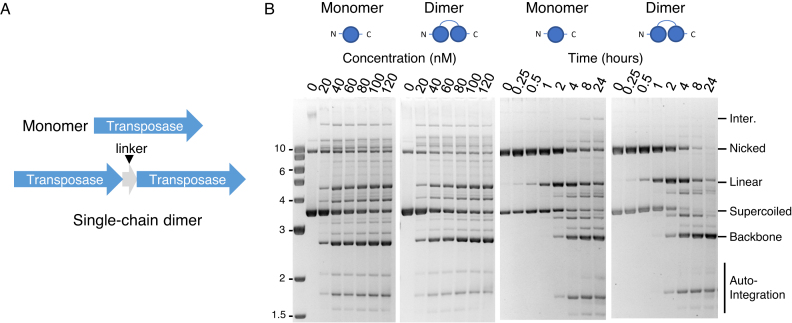
Activity of a Tn*5* transposase single-chain dimer. (**A**) The arrangement of the coding regions and the linker in the expression vectors for the monomeric and dimeric versions of the transposase are illustrated. (**B**) Concentration titrations and kinetic analyses of the monomeric and dimeric versions of the transposase. Transposition reactions contained 400 ng (7 nM) of plasmid substrate and 100 nM transposase unless stated otherwise. Photographs of ethidium bromide stained agarose gels are shown. An Illustration of the substrate and products are provided in Supplementary Figure S2.

To promote the self-association of Tn*5* transposase monomer we designed a single-chain dimer (Figure 2A). It is based on a hyperactive transposase with three point mutations: one eliminates an alternative start codon, one increases the affinity for the transposon end and the third makes the protein *trans*-acting by suppressing an inhibitory conformational change (28). In the post-cleavage structure of the hyperactive Tn*5* transpososome the distance between the C- and N-termini of the subunits is only 12 Å (40). To allow ample flexibility for potential conformational changes at other stages of the reaction we inserted a glycine- and serine-rich linker of 22 amino acids between the subunits. The monomeric and dimeric transposase were purified (Supplementary Figure S1) and assayed *in vitro* using a plasmid substrate (Figure 2B). The reaction yields an array of heterogeneous integration products, which are difficult to quantify (but see Supplementary Figure S2 and Refs. (32,41) for a description). However, excision of the transposon from the plasmid yields the plasmid backbone, which is a homogeneous end product of the reaction and a convenient measure of the overall efficiency. This is because almost all of the excised transposon goes onto complete integration if incubated long enough. Our first assay was to titrate the reaction with the monomeric and the single-chain dimeric transposases (Figure 2B). Although the reactions were qualitatively similar, the single-chain dimer appeared to be slightly less active because a greater amount of the protein was required to achieve maximum activity. Analysis of the reaction kinetics, using optimal amounts of each protein, produced almost identical band patterns. Since these assays have very little non-specific DNA, we would not expect to detect any putative advantage that the single-chain dimer may have in searching for the transposon ends.

### Transpososome assembly pathway

**Figure 3.**
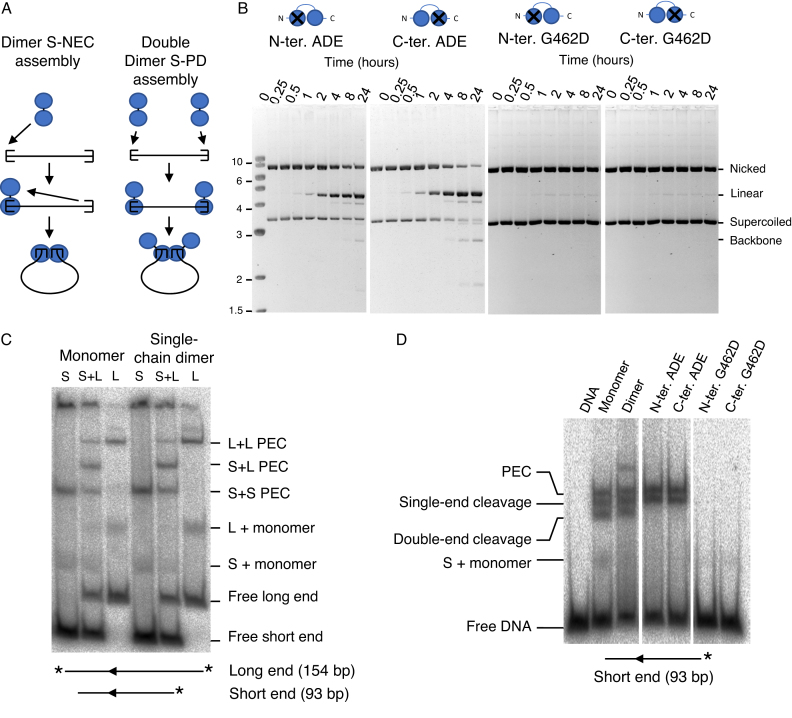
The single-chain dimeric assembles the transpososome by the S-NEC pathway. (**A**) The intended S-NEC pathway and the hypothetical double-dimer S-PD pathway are illustrated. In the double-dimer pathway the single-chain dimeric transposase assembles the transpososome by the S-PD pathway used by the monomeric form. (**B**) Kinetic analysis of single-chain dimer transposase containing point mutations in one of the two subunits. In the first pair of panels, the DDE motif, which coordinates the catalytic metal ion in the active site, is changed to ADE by site-directed mutagenesis. This yields linear plasmid when the single wild-type active site cleaves one of the transposon ends. This is the same result as with the equivalent mutation in an Hsmar1 transposase single-chain dimer (25). In the second pair of panels, the ability of the protein to dimerize is abolished by the G462D mutation (43). Photographs of ethidium bromide stained agarose gels are shown. (**C**) EMSA of the monomeric and single-chain dimeric transposases. Complexes were assembled using radiolabeled DNA fragments of different lengths. When both DNA fragments are included in the reaction mixture a new transpososome complex of intermediate mobility appears. This demonstrates that the complex contains a pair of transposon ends. The faint bands between the free DNA and the PECs is possibly the transposase bound to a single transposon end (L or S + monomer). This could either be present in the original binding reaction or produced by decay of the PEC during electrophoresis. The gel was recorded on a phosphorimager. *, position of the radioactive label. (**D**) EMSA of the cleavage reaction with the indicated transposase derivatives. Binding reactions were as in part C with the shorter of the two DNA fragments.

Although the activity of the monomer and single-chain dimer were similar, it is unclear whether the transpososome assembly mechanism is different. It is possible that assembly still proceeds via the S-PD mechanism with one single-chain dimer at each transposon end (Figure 3A). To address this issue we mutated one of the DDE motifs in the active sites of the single-chain dimer to ADE. In the monomeric protein, this abolishes the activity (42). This is because the transpososome has inactive subunits at both ends of the transposon. The mutated single-chain dimer yielded a significant amount of linear plasmid. This is produced by cleavage at one of the two transposon ends by the one active subunit in the dimer (Figure 3B). This is consistent with the S-NEC model for transpososome assembly in which the subunits in the single-chain dimer are engaged at either end of the same copy of the transposon. The reactions also produced a trace amount of plasmid backbone, which requires a functional active site at both ends of the element. We therefore conclude that although the predominant pathway for transpososome assembly is S-NEC, there may be a low background level of double-dimer complexes formed. Alternatively, the phenotype of the active site mutation may be slightly leaky.

To extend the test of the S-NEC model we introduced the G462D substitution into one or other of the subunits of the single-chain dimer. This mutation inhibits the activity of the monomeric transposase by destabilizing the dimer interface in the transpososome (43). In the single-chain dimer, the activity is abolished when the mutation is introduced into either of the subunits (Figure 3B). This is consistent with the S-NEC pathway for transpososome assembly. If the double-dimer pathway for assembly was operating, we would expect up to 50% activity, which is not observed.

We also compared the binding of the transposase monomer and single-chain dimer to linear DNA fragments in an EMSA (Figure 3C). The DNA fragments were of two different lengths, which we term long end and short end. When assembled using the short fragment, the transpososome complex migrates further down the gel. When long and short fragments are both included in the reaction, a mixed complex is detected at an intermediate position. This demonstrates that the transpososome complex contains a pair of transposon ends. There was no difference in the band pattern with transposase monomer and the single-chain dimer. This suggests that the single-chain dimer is able to assemble a normal transpososome.

Our final test was to examine the cleavage pattern produced by the monomer and single-chain dimer transposases in the EMSA. When the binding reaction is supplemented with Mg^2+^, the single-end and double-end cleavage products are detected (Figure 3D). The active site mutations in the single-chain dimer produced only single-end cleavage products, which is consistent with the plasmid assays in Figure 3B. No transpososome complex was detected for the dimer interface mutant, which is also consistent with the plasmid assays in Figure 3B. Overall, the behavior of the single-chain dimer mutants in the plasmid and EMSA assays is consistent with the S-NEC pathway for transpososome assembly.

### The single-chain dimer is hyperactive in bacteria

**Figure 4.**
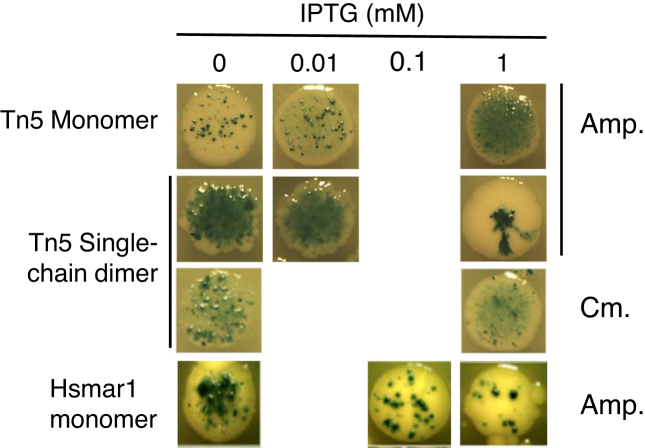
The transposase single-chain dimer is hyperactive in a bacterial papillation assay. Transposition events during the development of a bacterial colony are revealed as blue spots on a white background, due to mobilization of a promoterless *lacZ'* reporter transposon on the chromosome. The transposases were expressed from a *ptac* promoter, which is induced by IPTG. The indicated amounts of IPTG were added to the LB agar plates. The activity of the single-chain dimer promotes plasmid loss. Cells can be forced to retain the plasmid if a chloramphenicol selection marker is used. We do not use the chloramphenicol marker routinely because it stresses the cells. This becomes noticeable upon prolonged incubation and in experiments when multiple antibiotic markers are present. In one experiment, a chloramphenicol marker was added to the expression vector to force retention of the plasmid by the cells. Representative colonies are shown. The entire plates are shown in Supplementary Figure S3.

In our *in vitro* transposition reactions there is so little non-specific competitor DNA that the transposon-end interactions are unlikely to limit the rate. If non-specific binding sites are considered to be 1 bp apart, they are in 2000-fold excess over transposon ends in a 4 kb substrate plasmid. However, in *E. coli* there are three orders of magnitude more non-specific sites than in the *in vitro* assay (i.e. 4 MB genome versus 4 kb plasmid). We therefore tested the single-chain dimer in a bacterial papillation assay (38,44). In this assay, the mobilization of a promoter-less *lacZ* transposon is detected as blue spots on the background of a white colony on X-gal plates (Figure 4). The transposon is on the chromosome and the transposase is expressed from a *ptac* promoter on a plasmid. In the absence of induction, leaky expression of the monomeric transposase gives well defined blue spots on a white background. Induction of transposase expression with IPTG increases the number of spots and produces a blue-background halo, which is associated with a high rate of transposition.

At the lowest expression level, the single-chain dimer is much more active than the monomer and produces an almost completely blue colony (Figure 4). Full induction of transposase expression yields a colony morphology typical of an extremely high rate of transposition. The sectors spreading out toward the edge of the colony are produced by early transposition events when the colony contains only a handful of cells. The deep blue central spot and the sectors spreading out toward the edge are caused by transposition events very early in the development of the colony (45). Most of the colony is white because the cells have lost the transposase expression plasmid. This was confirmed by the lack of clones recovered after re-streaking on selective plates, and by performing plasmid mini-preps on clones recovered from non-selective plates. The reason that the colony grows even when most of the cells have lost the plasmid is that the β-lactamase diffuses through the plate and destroys the ampicillin. When we changed the selective marker on the plasmid to chloramphenicol it prevented growth of cells that had lost the plasmid, and the colonies on 1 mM IPTG media had a uniform coverage of blue papillae (Figure 4). For the Hsmar1 transposase, re-streaking of the colonies confirmed that the cells maintained the Hsmar1 transposase expression vector after induction. This is because Hsmar1 is autoregulated by over-production inhibition (OPI) (22,38).

OPI was first noted for Mos1 in *Drosophila mauritiana* and was subsequently observed in other mariner element (38,46,47,48). Much later, the phenomenon was explained as an emergent property of the transposition reaction, which arose from the multimerization of the transposase prior to DNA binding (22,35,49). OPI has been demonstrated for Hsmar1 in HeLa cells and *in vitro* (22,38). This is now confirmed by the bacteria papillation assay that shows that the highest rate of transposition is in the absence of induction (Figure 4). It is therefore noteworthy that the Tn*5* single-chain dimer does not behave in this way. Indeed, it was already clear from the *in vitro* transposase titration that the Tn*5* single-chain dimer is not inhibited by an 8-fold molar excess of the protein over transposon ends (Figure 2).

### The single-chain dimer is hyperactive in mammalian cells

**Figure 5.**
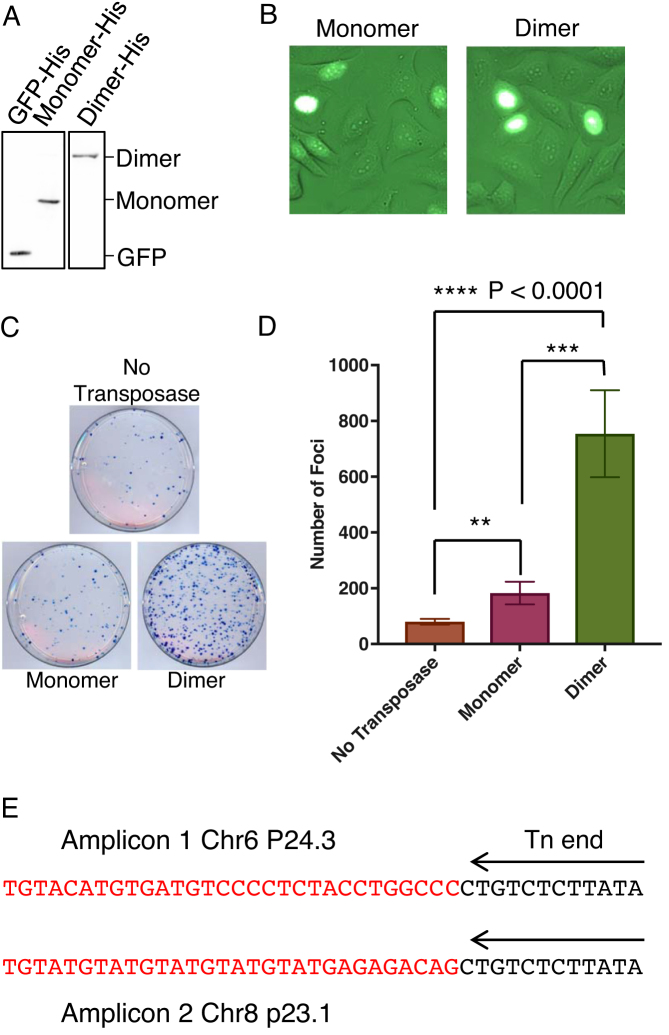
The transposase single-chain dimer is hyperactive in HeLa cells. (**A**) HeLa cells were transfected with plasmid expression vectors encoding the indicated His-tagged proteins. Cell extract was western blotted and probed with an anti-His-tag antibody. (**B**) A GFP moiety was added to the N-terminus of the transposases, which were imaged by fluorescence microscopy to confirm that the cells could transport the transposases to the nucleus. (**C**) A chromosomal integration assay was performed by transfecting cells with the transposase expression vector and a plasmid with a reporter transposon encoding a neomycin resistance marker. Following challenge with G418, surviving cells formed foci, which were detected by methylene blue staining. (**D**) Foci from part C were counted and plotted. Error bars are standard error of the mean, *n* = 8. Ratio paired *T*-test: **, *P* < 0.01; ***, *P* < 0.0003; ****, *P* < 0.0001; *n* = 8. (**E**) Two integration events from single-chain dimer transposition reactions in HeLa cells were mapped by PCR using genomic DNA from the surviving foci after G418 treatment.

The monomeric and single-chain dimeric transposases were codon optimized and cloned into the pcDNA3.1 expression vector, which has a strong viral promoter. The transposase genes had an NLS and a poly-histidine tag (His-tag) added at the 5′- and 3′-ends, respectively. The level of expression after transfection into HeLa cells was assayed by western blotting using an anti-His-tag antibody, with His-tagged GFP cloned in the same vector as a positive control (Figure 5A). The single-chain dimer was expressed less strongly than the other two proteins. To determine whether the transposase was transported to the nucleus, we fused GFP to the transposases and examined the cells by fluorescent microscopy (Figure 5B). This confirmed that the cells were capable of transporting the transposase to the nucleus.

To determine the rate of transposition in HeLa cells we took advantage of a widely-used two-plasmid transfection assay. One plasmid was the transposase expression vector, while the other carried a reporter-transposon encoding a neomycin-resistance marker. One day after transfection, the cells were challenged with G418. Cells in which the transposon had been integrated into the genome went on to develop into foci, which were stained with methylene blue and counted (Figure 5C and D). The number of foci with the monomeric transposase is about 2-fold above the background of illegitimate recombination of the reporter plasmid into the genome. After accounting for the background, the single-chain dimer is about 7-fold more active than the monomeric transposase, despite the fact that it was expressed at a significantly lower level. The transposon integration was verified by PCR on genomic DNA from two foci (Figure 5E).

## DISCUSSION

Monomeric and dimeric members of the DDE(D) superfamily of transposases appear to use distinct pathways for assembly of the transpososome (Figure 1A and B). The dimeric enzymes interact first with one transposon end and then recruit a second unbound end. The monomeric transposases appear to multimerize only after interacting with the transposon end sequence (e.g. (26,27,28,29)). All other things being equal, this type of reaction will be slower because more components have to come together in one place at the same time. Multimerization in advance of DNA binding would therefore appear to be advantageous in searching for transposon ends in a large genome.

We have explored the effects of transposase multimerization by creating a single-chain dimer of the Tn*5* transposase (Figure 2). This had the predicted effect of changing the pathway of transpososome assembly from S-PD to S-NEC (Figure 3). This was confirmed by introducing active site and dimerization mutations into one of the two subunits in the single-chain dimer (Figure 3B). The strongest evidence was from the G462D mutant, which disrupts the dimer interface (43). When present in either subunit of the single-chain dimer, the mutation abolishes the activity completely and therefore excludes any residual double-dimer S-PD assembly (Figure 3A). Since the single-chain dimer behaves normally in the cleavage and EMSA assays, it appears that the switch from the S-PD to S-NEC pathway does not affect subsequent steps of the reaction (Figures 2B, 3C and D).

**Figure 6.**
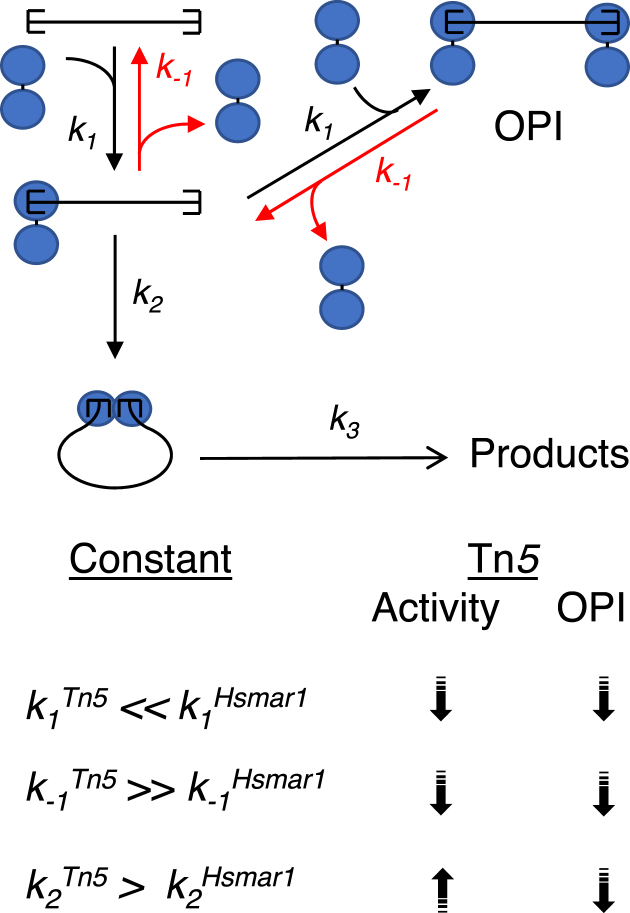
Kinetic diagram of the S-NEC pathway and mechanism of OPI. The association and dissociation rate constants for transposase binding are *k_1_* and *k_-1_*. Double occupancy of the transposon ends inhibits the reaction because there are no unbound ends for recruitment into the transpososome. Synapsis (*k_2_*) is considered to be irreversible and to represent a commitment to transposon excision. All subsequent steps of the reaction can be subsumed into a single rate constant, *k*_3_ (22). The relative magnitudes of the rate constants for Tn*5* and Hsmar1 are summarized below the diagram. For *Tn5*, the relative values of *k_1_* and *k_-1_* are unfavorable for OPI.

*In vivo*, the single-chain dimer was hyperactive in the bacterial and HeLa cell assays (Figures 4 and 5). Presumably, the S-NEC pathway increases the frequency at which a pair of transposon ends will interact with a pair of transposase subunits. In Hsmar1, a eukaryotic member of the DDE(D) family, OPI results from the double occupancy of the transposon ends by transposase dimers (Figure 6). This inhibits the reaction because there are no free ends for recruitment into the developing transpososome (22). However, the Tn*5* single-chain dimer was not subject to OPI *in vitro* or in the bacterial assay (Figures 2 and 3).

The absence of OPI in Tn*5* is probably due to weak single-end binding. Several lines of evidence support the idea that the association and dissociation rate constants, *k_1_* and *k_-1_*, are unfavorable to OPI (Figure 6). Tn*5* transposase has significant non-specific DNA binding activity and only a single alpha helix that makes sequence-specific interactions in the major groove of the DNA at the transposon end (40). The association rate constant, *k_1_*, must therefore be relatively low, although it has not been measured. The lack of extensive sequence-specific interactions also means that the dissociation rate constant, *k_-1_*, must be relatively high. This is reflected in the fact that little or no single-end complex is detected in an EMSA with the G462D dimerization mutant, which prevents assembly of the paired-ends complex (Figure 3D) (43). The combination of a low value of *k_1_* and a high value of *k_-1_* are unfavorable for OPI because there will always be free transposon ends available for recruitment into the S-NEC pathway (Figure 6).

In contrast to the monomeric bacterial enzymes, the human Hsmar1 transposase has several properties that enhance OPI. The first is that it forms a stable dimer in the absence of DNA. Subunit exchange experiments estimated t_1/2_ at about 3 h, which corresponds to a dissociation rate constant of 6 × 10^−5^ s^−1^ (22). This is very stable; for example, the Cro repressor subunits have a t_1/2_ of about 23 s (50). Next, the Hsmar1 transposase has a low affinity for non-specific DNA and two helix-turn-helix domains for sequence-specific binding (22,32,51). This means that the value of *k_1_* will be high compared to Tn*5* transposase. Single-end binding in Hsmar1 is further increased by an unusually long dwell time (low *k_-1_*), which we estimated at about 10 min. This is also very stable; typical helix-turn-helix proteins have dwell times of <1 min (22). Finally, single-end binding is accompanied by a conformational change that sharply reduces the affinity of the second transposase monomer for the second transposon end. This equates to a lower than expected value for the association rate constant *k_2_*. Together, these factors enhance OPI and provide Hsmar1 with a mechanism for autoregulation.

Are there any other ways in which Tn*5* transposition could be improved in the large genome of a eukaryotic cell? One approach would be to fuse the single-chain dimer to a strong DNA-binding domain and to add its cognate binding-site next to the transposon end. This would boost *k_1_*^App.^ and reduce *k_-1_*^App.^ by tethering the transposase close to the transposon end.

## Supplementary Material

Supplementary Data
