# Supplementary material for: Transposase subunit architecture and its relationship to genome size and the rate of transposition in prokaryotes and eukaryotes
Source: Nucleic Acids Res. 2018 Sep 4;46(18):9637–46. doi: 10.1093/nar/gky794 (PMC6182136; doi:10.1093/nar/gky794)
Supplement: Supplementary Data [file gky794_supplemental_files.pdf]

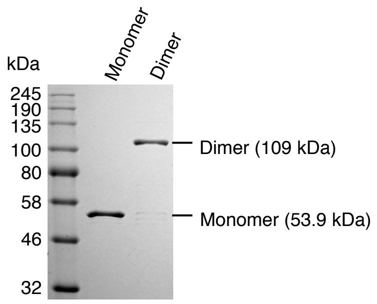

Supplementary Figure 1 SDS PAGE analysis of purified transposases

The monomeric and single-chain dimeric transposase were purified using intein cleavage off a chitin affinity column as described in the Methods section. Each lane was loaded with 1 μg of protein.

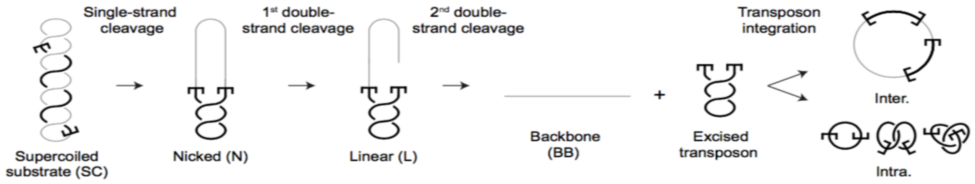

Supplementary Figure 2 The steps of the transposition reaction are illustrated.

Integration may be intermolecular into an unreacted substrate plasmid or intramolecular to yield autointegration products.

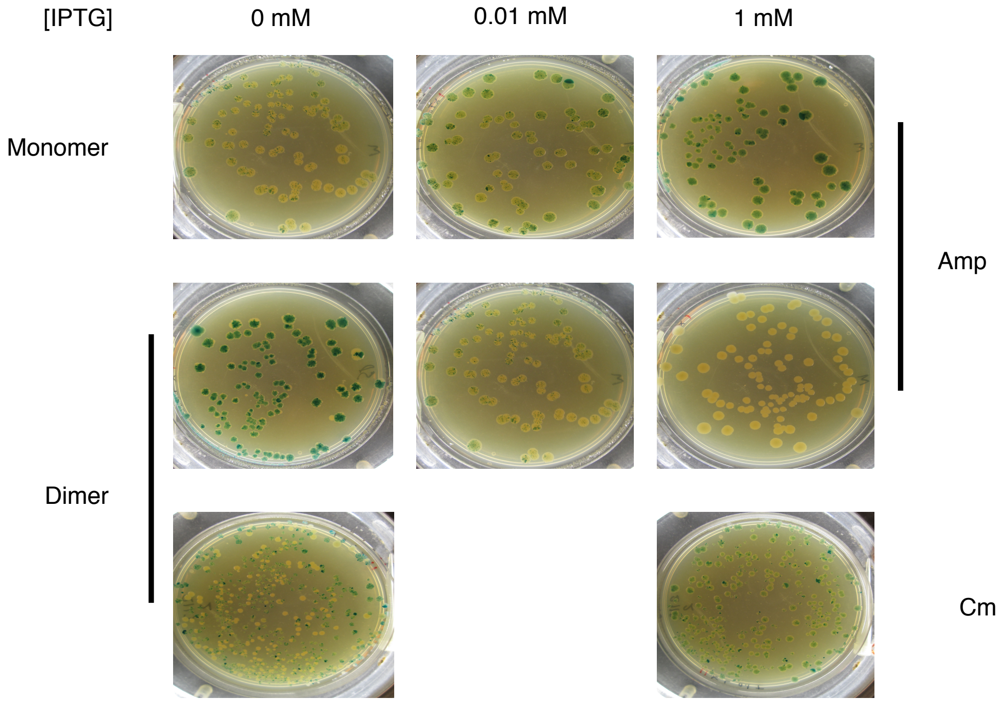

Supplementary Figure 3 Papillation plates

Full plate images of the assay shown in Figure 4.

Supplemental Table 1 Plasmid Sequences

pRC681
gaattcTATTAGGTTGGTGCAAAAGTAATTGCGGTTTTggatccATGACCATGA
TTACGGATTCACTGGCCGTCGTTTTACAACGTCGTGACTGGGAAAACCC
TGGCGTTACCCAACTTAATCGCCTTGCAGCACATCCCCCTTTCGCCAGC
TGGCGTAATAGCGAAGAGGCCCGCACCGATCGCCCTTCCCAACAGTTG
CGCAGCCTGAATGGCGAATGGCGCTTTGCCTGGTTTCCGGCACCAGAA
GCGGTGCCGGAAAGCTGGCTGGAGTGCGATCTTCCTGAGGCCGATACT
GTCGTCGTCCCCTCAAACTGGCAGATGCACGGTTACGATGCGCCCATCT
ACACCAACGTGACCTATCCCATTACGGTCAATCCGCCGTTTGTTCCCAC
GGAGAATCCGACGGGTTGTTACTCGCTCACATTTAATGTTGATGAAAGCT
GGCTACAGGAAGGCCAGACGCGAATTATTTTTGATGGCGTTAACTCGGC
GTTTCATCTGTGGTGCAACGGGCGCTGGGTCGGTTACGGCCAGGACAG
TCGTTTGCCGTCTGAATTTGACCTGAGCGCATTTTTACGCGCCGGAGAA
AACCGCCTCGCGGTGATGGTGCTGCGCTGGAGTGACGGCAGTTATCTG
GAAGATCAGGATATGTGGCGGATGAGCGGCATTTTCCGTGACGTCTCGT
TGCTGCATAAACCGACTACACAAATCAGCGATTTCCATGTTGCCACTCGC
TTTAATGATGATTTCAGCCGCGCTGTACTGGAGGCTGAAGTTCAGATGTG
CGGCGAGTTGCGTGACTACCTACGGGTAACAGTTTCTTTATGGCAGGGT
GAAACGCAGGTCGCCAGCGGCACCGCGCCTTTCGGCGGTGAAATTATC
GATGAGCGTGGTGGTTATGCCGATCGCGTCACACTACGTCTGAACGTCG
AAAACCCGAAACTGTGGAGCGCCGAAATCCCGAATCTCTATCGTGCGGT
GGTTGAACTGCACACCGCCGACGGCACGCTGATTGAAGCAGAAGCCTG
CGATGTCGGTTTCCGCGAGGTGCGGATTGAAAATGGTCTGCTGCTGCTG
AACGGCAAGCCGTTGCTGATTCGAGGCGTTAACCGTCACGAGCATCATC
CTCTGCATGGTCAGGTCATGGATGAGCAGACGATGGTGCAGGATATCCT
GCTGATGAAGCAGAACAACTTTAACGCCGTGCGCTGTTCGCATTATCCG
AACCATCCGCTGTGGTACACGCTGTGCGACCGCTACGGCCTGTATGTGG
TGGATGAAGCCAATATTGAAACCCACGGCATGGTGCCAATGAATCGTCT
GACCGATGATCCGCGCTGGCTACCGGCGATGAGCGAACGCGTAACGCG
AATGGTGCAGCGCGATCGTAATCACCCGAGTGTGATCATCTGGTCGCTG
GGGAATGAATCAGGCCACGGCGCTAATCACGACGCGCTGTATCGCTGG
ATCAAATCTGTCGATCCTTCCCGCCCGGTGCAGTATGAAGGCGGCGGAG
CCGACACCACGGCCACCGATATTATTTGCCCGATGTACGCGCGCGTGGA
TGAAGACCAGCCCTTCCCGGCTGTGCCGAAATGGTCCATCAAAAAATGG
CTTTCGCTACCTGGAGAGACGCGCCCGCTGATCCTTTGCGAATACGCCC
ACGCGATGGGTAACAGTCTTGGCGGTTTCGCTAAATACTGGCAGGCGTT
TCGTCAGTATCCCCGTTTACAGGGCGGCTTCGTCTGGGACTGGGTGGAT
CAGTCGCTGATTAAATATGATGAAAACGGCAACCCGTGGTCGGCTTACG
GCGGTGATTTTGGCGATACGCCGAACGATCGCCAGTTCTGTATGAACGG
TCTGGTCTTTGCCGACCGCACGCCGCATCCAGCGCTGACGGAAGCAAA
ACACCAGCAGCAGTTTTTCCAGTTCCGTTTATCCGGGCAAACCATCGAA
GTGACCAGCGAATACCTGTTCCGTCATAGCGATAACGAGCTCCTGCACT
GGATGGTGGCGCTGGATGGTAAGCCGCTGGCAAGCGGTGAAGTGCCTC
TGGATGTCGCTCCACAAGGTAAACAGTTGATTGAACTGCCTGAACTACC
GCAGCCGGAGAGCGCCGGGCAACTCTGGCTCACAGTACGCGTAGTGCA
ACCGAACGCGACCGCATGGTCAGAAGCCGGGCACATCAGCGCCTGGCA
GCAGTGGCGTCTGGCGGAAAACCTCAGTGTGACGCTCCCCGCCGCGTC
CCACGCCATCCCGCATCTGACCACCAGCGAAATGGATTTTTGCATCGAG

CTGGGTAATAAGCGTTGGCAATTTAACCGCCAGTCAGGCTTTCTTTCACA
GATGTGGATTGGCGATAAAAAACAACTGCTGACGCCGCTGCGCGATCAG
TTCACCCGTGCACCGCTGGATAACGACATTGGCGTAAGTGAAGCGACCC
GCATTGACCCTAACGCCTGGGTCGAACGCTGGAAGGCGGCGGGCCATT
ACCAGGCCGAAGCAGCGTTGTTGCAGTGCACGGCAGATACACTTGCTGA
TGCGGTGCTGATTACGACCGCTCACGCGTGGCAGCATCAGGGGAAAAC
CTTATTTATCAGCCGGAAAACCTACCGGATTGATGGTAGTGGTCAAATGG
CGATTACCGTTGATGTTGAAGTGGCGAGCGATACACCGCATCCGGCGC
GGATTGGCCTGAACTGCCAGCTGGCGCAGGTAGCAGAGCGGGTAAACT
GGCTCGGATTAGGGCCGCAAGAAAACTATCCCGACCGCCTTACTGCCG
CCTGTTTTGACCGCTGGGATCTGCCATTGTCAGACATGTATACCCCGTAC
GTCTTCCCGAGCGAAAACGGTCTGCGCTGCGGGACGCGCGAATTGAAT
TATGGCCCACACCAGTGGCGCGGCGACTTCCAGTTCAACATCAGCCGCT
ACAGTCAACAGCAACTGATGGAAACCAGCCATCGCCATCTGCTGCACGC
GGAAGAAGGCACATGGCTGAATATCGACGGTTTCCATATGGGGATTGGT
GGCGACGACTCCTGGAGCCCGTCAGTATCGGCGGAATTCCAGCTGAGC
GCCGGTCGCTACCATTACCAGTTGGTCTGGTGTCAAAAATAATGTCTCAA
AATCTCTGATGTTACATTGCACAAGATAAAAATATATCATCATGAACAATA
AAACTGTCTGCTTACATAAACAGTAATACAAGGGGTGTTATGAGCCATAT
TCAACGGGAAACGTCTTGCTCGAGGCCGCGATTAAATTCCAACATGGAT
GCTGATTTATATGGGTATAAATGGGCTCGCGATAATGTCGGGCAATCAG
GTGCGACAATCTATCGATTGTATGGGAAGCCCGATGCGCCAGAGTTGTT
TCTGAAACATGGCAAAGGTAGCGTTGCCAATGATGTTACAGATGAGATG
GTCAGACTAAACTGGCTGACGGAATTTATGCCTCTTCCGACCATCAAGCA
TTTTATCCGTACTCCTGATGATGCATGGTTACTCACCACTGCGATCCCCG
GGAAAACAGCATTCCAGGTATTAGAAGAATATCCTGATTCAGGTGAAAAT
ATTGTTGATGCGCTGGCAGTGTTCCTGCGCCGGTTGCATTCGATTCCTG
TTTGTAATTGTCCTTTTAACAGCGATCGCGTATTTCGTCTCGCTCAGGCG
CAATCACGAATGAATAACGGTTTGGTTGATGCGAGTGATTTTGATGACGA
GCGTAATGGCTGGCCTGTTGAACAAGTCTGGAAAGAAATGCATAAGCTT
TTGCCATTCTCACCGGATTCAGTCGTCACTCATGGTGATTTCTCACTTGA
TAACCTTATTTTTGACGAGGGGAAATTAATAGGTTGTATTGATGTTGGAC
GAGTCGGAATCGCAGACCGATACCAGGATCTTGCCATCCTATGGAACTG
CCTCGGTGAGTTTTCTCCTTCATTACAGAAACGGCTTTTTCAAAAATATG
GTATTGATAATCCTGATATGAATAAATTGCAGTTTCATTTGATGCTCGATG
AGTTTTTCTAATCAGAATTGGTTAATTGGTTGTAACACTGGCAGAGCATTA
CGCTGACTTGACGGGACGGCGGCTTTGTTGAATAAATCGAACTTTTGCT
GAGTTGAAGGATCAGATCACGCATCTTCCCGACAACGCAGACCGTTCCG
TGGCAAAGCAAAAGTTCAAAATCACCAACTGGTCCACCTACAACAAAggat
ccAAAACCGCAATTACTTTTGCACCAACCTAATAgaattCTGGCGTAATAGC
GAAGAGGCCCGCACCGATCGCCCTTCCCAACAGTTGCGCAGCCTGAAT
GGCGAATGGGACGCGCCCTGTAGCGGCGCATTAAGCGCGGCGGGTGT
GGTGGTTACGCGCAGCGTGACCGCTACACTTGCCAGCGCCCTAGCGCC
CGCTCCTTTCGCTTTCTTCCCTTCCTTTCTCGCCACGTTCGCCGGCTTTC
CCCGTCAAGCTCTAAATCGGGGGCTCCCTTTAGGGTTCCGATTTAGTGC
TTTACGGCACCTCGACCCCAAAAAACTTGATTAGGGTGATGGTTCACGTA
GTGGGCCATCGCCCTGATAGACGGTTTTTCGCCCTTTGACGTTGGAGTC
CACGTTCTTTAATAGTGGACTCTTGTTCCAAACTGGAACAACACTCAACC
CTATCTCGGTCTATTCTTTTGATTTATAAGGGATTTTGCCGATTTCGGCCT
ATTGGTTAAAAAATGAGCTGATTTAACAAAAATTTAACGCGAATTTTAACA

AAATATTAACGCTTACAATTTAGGTGGCACTTTTCGGGGAAATGTGCGCG
GAACCCCTATTTGTTTATTTTTCTAAATACATTCAAATATGTATCCGCTCAT
GAGACAATAACCCTGATAAATGCTTCAATAATATTGAAAAAGGAAGAGTA
TGAGTATTCAACATTTCCGTGTCGCCCTTATTCCCTTTTTTGCGGCATTTT
GCCTTCCTGTTTTTGCTCACCCAGAAACGCTGGTGAAAGTAAAAGATGCT
GAAGATCAGTTGGGTGCACGAGTGGGTTACATCGAACTGGATCTCAACA
GCGGTAAGATCCTTGAGAGTTTTCGCCCCGAAGAACGTTTTCCAATGAT
GAGCACTTTTAAAGTTCTGCTATGTGGCGCGGTATTATCCCGTATTGACG
CCGGGCAAGAGCAACTCGGTCGCCGCATACACTATTCTCAGAATGACTT
GGTTGAGTACTCACCAGTCACAGAAAAGCATCTTACGGATGGCATGACA
GTAAGAGAATTATGCAGTGCTGCCATAACCATGAGTGATAACACTGCGG
CCAACTTACTTCTGACAACGATCGGAGGACCGAAGGAGCTAACCGCTTT
TTTGCACAACATGGGGGATCATGTAACTCGCCTTGATCGTTGGGAACCG
GAGCTGAATGAAGCCATACCAAACGACGAGCGTGACACCACGATGCCTG
TAGCAATGGCAACAACGTTGCGCAAACTATTAACTGGCGAACTACTTACT
CTAGCTTCCCGGCAACAATTAATAGACTGGATGGAGGCGGATAAAGTTG
CAGGACCACTTCTGCGCTCGGCCCTTCCGGCTGGCTGGTTTATTGCTGA
TAAATCTGGAGCCGGTGAGCGTGGGTCTCGCGGTATCATTGCAGCACTG
GGGCCAGATGGTAAGCCCTCCCGTATCGTAGTTATCTACACGACGGGGA
GTCAGGCAACTATGGATGAACGAAATAGACAGATCGCTGAGATAGGTGC
CTCACTGATTAAGCATTGGTAACTGTCAGACCAAGTTTACTCATATATACT
TTAGATTGATTTAAAACTTCATTTTTAATTTAAAAGGATCTAGGTGAAGAT
CCTTTTTGATAATCTCATGACCAAAATCCCTTAACGTGAGTTTTCGTTCCA
CTGAGCGTCAGACCCCGTAGAAAAGATCAAAGGATCTTCTTGAGATCCTT
TTTTTCTGCGCGTAATCTGCTGCTTGCAAACAAAAAAACCACCGCTACCA
GCGGTGGTTTGTTTGCCGGATCAAGAGCTACCAACTCTTTTTCCGAAGG
TAACTGGCTTCAGCAGAGCGCAGATACCAAATACTGTCCTTCTAGTGTAG
CCGTAGTTAGGCCACCACTTCAAGAACTCTGTAGCACCGCCTACATACC
TCGCTCTGCTAATCCTGTTACCAGTGGCTGCTGCCAGTGGCGATAAGTC
GTGTCTTACCGGGTTGGACTCAAGACGATAGTTACCGGATAAGGCGCAG
CGGTCGGGCTGAACGGGGGGTTCGTGCACACAGCCCAGCTTGGAGCGA
ACGACCTACACCGAACTGAGATACCTACAGCGTGAGCTATGAGAAAGCG
CCACGCTTCCCGAAGGGAGAAAGGCGGACAGGTATCCGGTAAGCGGCA
GGGTCGGAACAGGAGAGCGCACGAGGGAGCTTCCAGGGGGAAACGCC
TGGTATCTTTATAGTCCTGTCGGGTTTCGCCACCTCTGACTTGAGCGTCG
ATTTTTGTGATGCTCGTCAGGGGGGCGGAGCCTATGGAAAAACGCCAGC
AACGCGGCCTTTTTACGGTTCCTGGCCTTTTGCTGGCCTTTTGCTCACAT
GTTCTTTCCTGCGTTATCCCCTGATTCTGTGGATAACCGTATTACCGCCT
TTGAGTGAGCTGATACCGCTCGCCGCAGCCGAACGACCGAGCGCAGCG
AGTCAGTGAGCGAGGAAGCGGAAGAGCGCCCAATACGCAAACCGCCTC
TCCCCGCGCGTTGGCCGATTCATTAATGCAG

pRC916
GCGCCCAATACGCAAACCGCCTCTCCCCGCGCGTTGGCCGATTCATTAA
TGCAGCTGGCACGACAGGTTTCCCGACTGGAAAGCGGGCAGTGAGCGC
AACGCAATTAATGTGAGTTAGCTCACTCATTAGGCACCCCAGGCTTTACA
CTTTATGCTTCCGGCTCGTATGTTGTGTGGAATTGTGAGCGGATAACAAT
TTCACACAGGAAACAGCTATGACCATGATTACGCCAAGCTCTAATACGAC
TCACTATAGGGAAAGCTCGGTACCACGCATGCTGCAGACGCGTTACGTA
TCGGATCCAGAATTCGTGAGCGTGGGTCTCGCGGTCTGTCTCTTATACA

CATCTCAACCATCATCGACAAGCTTCTCGAGCCTAGGCTAGCTCTAGAC
CACACGTGTGGGGGCCCGAGCTCGCGGCCGCTGTATTCTATAGTGTCA
CCTAAATGGCCGCACAATTCACTGGCCGTCGTTTTACAACGTCGTGACT
GGGAAAACCCTGGCGTTACCCAACTTAATCGCCTTGCAGCACATCCCCC
TTTCGCCAGCTGGCGTAATAGCGAAGAGGCCCGCACCGATCGCCCTTC
CCAACAGTTGCGCAGCCTGAATGGCGAATGGAAATTGTAAGCGTTAATA
TTTTGTTAAAATTCGCGTTAAATTTTTGTTAAATCAGCTCATTTTTTAACCA
ATAGGCCGAAATCGGCAAAATCCCTTATAAATCAAAAGAATAGACCGAGA
TAGGGTTGAGTGTTGTTCCAGTTTGGAACAAGAGTCCACTATTAAAGAAC
GTGGACTCCAACGTCAAAGGGCGAAAAACCGTCTATCAGGGCGATGGC
CCACTACGTGAACCATCACCCTAATCAAGTTTTTTGGGGTCGAGGTGCC
GTAAAGCACTAAATCGGAACCCTAAAGGGAGCCCCCGATTTAGAGCTTG
ACGGGGAAAGCCGGCGAACGTGGCGAGAAAGGAAGGGAAGAAAGCGA
AAGGAGCGGGCGCTAGGGCGCTGGCAAGTGTAGCGGTCACGCTGCGC
GTAACCACCACACCCGCCGCGCTTAATGCGCCGCTACAGGGCGCGTCA
GGTGGCACTTTTCGGGGAAATGTGCGCGGAACCCCTATTTGTTTATTTTT
CTAAATACATTCAAATATGTATCCGCTCATGAGACAATAACCCTGATAAAT
GCTTCAATAATATTGAAAAAGGAAGAGTATGAGTATTCAACATTTCCGTGT
CGCCCTTATTCCCTTTTTTGCGGCATTTTGCCTTCCTGTTTTTGCTCACCC
AGAAACGCTGGTGAAAGTAAAAGATGCTGAAGATCAGTTGGGTGCACGA
GTGGGTTACATCGAACTGGATCTCAACAGCGGTAAGATCCTTGAGAGTT
TTCGCCCCGAAGAACGTTTTCCAATGATGAGCACTTTTAAAGTTCTGCTA
TGTGGCGCGGTATTATCCCGTATTGACGGAGTCAGGCAACTATGGATGA
ACGAAATAGACAGATCGCTGAGATAGGTGCCTCACTGATTAAGCATTGG
TAACTGTCAGACCAAGTTTACTCATATATACTTTAGATTGATTTAAAACTT
CATTTTTAATTTAAAAGGATCTAGGTGAAGATCCTTTTTGATAATCTCATG
AACAATAAAACTGTCTGCTTACATAAACAGTAATACAAGGGGTGTTATGA
GCCATATTCAACGGGAAACGTCTTGCTCTAGGCCGCGATTAAATTCCAAC
ATGGATGCTGATTTATATGGGTATAAATGGGCTCGCGATAATGTCGGGC
AATCAGGTGCGACAATCTATCGATTGTATGGGAAGCCCGATGCGCCAGA
GTTGTTTCTGAAACATGGCAAAGGTAGCGTTGCCAATGATGTTACAGATG
AGATGGTCAGACTAAACTGGCTGACGGAATTTATGCCTCTTCCGACCATC
AAGCATTTTATCCGTACTCCTGATGATGCATGGTTACTCACCACTGCGAT
CCCCGGGAAAACAGCATTCCAGGTATTAGAAGAATATCCTGATTCAGGT
GAAAATATTGTTGATGCGCTGGCAGTGTTCCTGCGCCGGTTGCATTCGA
TTCCTGTTTGTAATTGTCCTTTTAACAGCGATCGCGTATTTCGTCTCGCTC
AGGCGCAATCACGAATGAATAACGGTTTGGTTGATGCGAGTGATTTTGAT
GACGAGCGTAATGGCTGGCCTGTTGAACAAGTCTGGAAAGAAATGCATA
AACTTTTGCCATTCTCACCGGATTCAGTCGTCACTCATGGTGATTTCTCA
CTTGATAACCTTATTTTTGACGAGGGGAAATTAATAGGTTGTATTGATGTT
GGACGAGTCGGAATCGCAGACCGATACCAGGATCTTGCCATCCTATGGA
ACTGCCTCGGTGAGTTTTCTCCTTCATTACAGAAACGGCTTTTTCAAAAAT
ATGGTATTGATAATCCTGATATGAATAAATTGCAGTTTCATTTGATGCTCG
ATGAGTTTTTCTAAGAATTAATTCATGACCAAAATCCCTTAACGTGAGTTT
TCGTTCCACTGAGCGTCAGACCCCGTAGAAAAGATCAAAGGATCTTCTT
GAGATCCTTTTTTTCTGCGCGTAATCTGCTGCTTGCAAACAAAAAAACCA
CCGCTACCAGCGGTGGTTTGTTTGCCGGATCAAGAGCTACCAACTCTTT
TTCCGAAGGTAACTGGCTTCAGCAGAGCGCAGATACCAAATACTGTCCT
TCTAGTGTAGCCGTAGTTAGGCCACCACTTCAAGAACTCTGTAGCACCG
CCTACATACCTCGCTCTGCTAATCCTGTTACCAGTGGCTGCTGCCAGTG

GCGATAAGTCGTGTCTTACCGGGTTGGACTCAAGACGATAGTTACCGGA
TAAGGCGCAGCGGTCGGGCTGAACGGGGGGTTCGTGCACACAGCCCA
GCTTGGAGCGAACGACCTACACCGAACTGAGATACCTACAGCGTGAGCT
ATGAGAAAGCGCCACGCTTCCCGAAGGGAGAAAGGCGGACAGGTATCC
GGTAAGCGGCAGGGTCGGAACAGGAGAGCGCACGAGGGAGCTTCCAG
GGGGAAACGCCTGGTATCTTTATAGTCCTGTCGGGTTTCGCCACCTCTG
ACTTGAGCGTCGATTTTTGTGATGCTCGTCAGGGGGGCGGAGCCTATGG
AAAAACGCCAGCAACGCGGCCTTTTTACGGTTCCTGGCCTTTTGCTGGC
CTTTTGCTCACATGTTCTTTCCTGCGTTATCCCCTGATTCTGTGGATAACC
GTATTACCGCCTTTGAGTGAGCTGATACCGCTCGCCGCAGCCGAACGAC
CGAGCGCAGCGAGTCAGTGAGCGAGGAAGCGGAAGA

pRC1721
CCGACACCATCGAATGGTGCAAAACCTTTCGCGGTATGGCATGATAGCG
CCCGGAAGAGAGTCAATTCAGGGTGGTGAATGTGAAACCAGTAACGTTA
TACGATGTCGCAGAGTATGCCGGTGTCTCTTATCAGACCGTTTCCCGCG
TGGTGAACCAGGCCAGCCACGTTTCTGCGAAAACGCGGGAAAAAGTGG
AAGCGGCGATGGCGGAGCTGAATTACATTCCCAACCGCGTGGCACAAC
AACTGGCGGGCAAACAGTCGTTGCTGATTGGCGTTGCCACCTCCAGTCT
GGCCCTGCACGCGCCGTCGCAAATTGTCGCGGCGATTAAATCTCGCGC
CGATCAACTGGGTGCCAGCGTGGTGGTGTCGATGGTAGAACGAAGCGG
CGTCGAAGCCTGTAAAGCGGCGGTGCACAATCTTCTCGCGCAACGCGT
CAGTGGGCTGATCATTAACTATCCGCTGGATGACCAGGATGCCATTGCT
GTGGAAGCTGCCTGCACTAATGTTCCGGCGTTATTTCTTGATGTCTCTGA
CCAGACACCCATCAACAGTATTATTTTCTCCCATGAAGACGGTACGCGAC
TGGGCGTGGAGCATCTGGTCGCATTGGGTCACCAGCAAATCGCGCTGTT
AGCGGGCCCATTAAGTTCTGTCTCGGCGCGTCTGCGTCTGGCTGGCTG
GCATAAATATCTCACTCGCAATCAAATTCAGCCGATAGCGGAACGGGAA
GGCGACTGGAGTGCCATGTCCGGTTTTCAACAAACCATGCAAATGCTGA
ATGAGGGCATCGTTCCCACTGCGATGCTGGTTGCCAACGATCAGATGGC
GCTGGGCGCAATGCGCGCCATTACCGAGTCCGGGCTGCGCGTTGGTGC
GGATATCTCGGTAGTGGGATACGACGATACCGAAGACAGCTCATGTTAT
ATCCCGCCGTTAACCACCATCAAACAGGATTTTCGCCTGCTGGGGCAAA
CCAGCGTGGACCGCTTGCTGCAACTCTCTCAGGGCCAGGCGGTGAAGG
GCAATCAGCTGTTGCCCGTCTCACTGGTGAAAAGAAAAACCACCCTGGC
GCCCAATACGCAAACCGCCTCTCCCCGCGCGTTGGCCGATTCATTAATG
CAGCTGGCACGACAGGTTTCCCGACTGGAAAGCGGGCAGTGAGCGCAA
CGCAATTAATGTGAGTTAGCTCACTCATTAGGCACAATTCTCATGTTTGA
CAGCTTATCATCGACTGCACGGTGCACCAATGCTTCTGGCGTCAGGCAG
CCATCGGAAGCTGTGGTATGGCTGTGCAGGTCGTAAATCACTGCATAAT
TCGTGTCGCTCAAGGCGCACTCCCGTTCTGGATAATGTTTTTTGCGCCG
ACATCATAACGGTTCTGGCAAATATTCTGAAATGAGCTGTTGACAATTAAT
CATCGGCTCGTATAATGTGTGGAATTGTGAGCGGATAACAATTTCACACA
GGAAACAGCCAGTCCGTTTAGGTGTTTTCACGAGCACTTCACCAACAAG
GACCATAGCATATGGAAATGATGCTCGATAAGAAACAGATTCGTGCGATC
TTTCTCTTTGAGTTTAAAATGGGTCGCAAAGCGGCGGAGACGACGCGTA
ATATTAACAACGCGTTCGGTCCTGGCACCGCGAACGAGCGTACCGTGCA
ATGGTGGTTCAAAAAGTTTCGCAAAGGCGACGAATCTCTGGAGGACGAA
GAGCGTTCTGGCCGCCCGTCCGAGGTTGACAACGACCAGCTGCGTGCA
ATCATCGAAGCTGATCCGCTGACTACCACCCGCGAAGTTGCTGAAGAAC

TGAATGTGGATCACTCTACTGTGGTTCGCCACCTGAAACAGATCGGTAAA
GTAAAAAAACTGGACAAATGGGTTCCTCATGAACTGTCTGAAAACCAGAA
AAACCGTCGTTTCGAAGTTAGCTCCTCTCTGATTCTGCGTAACAACAACG
AACCGTTCCTGGATCGTATCGTAACCTGTGATGAGAAATGGATTCTGTAT
GATAACCGTCGCCGCTCTGCTCAGTGGCTGGATCGCGAAGAAGCTCCAA
AACACTTCCCGAAACCGAATCTGCACCAGAAGAAAGTCATGGTAACCGT
ATGGTGGTCTGCCGCAGGTGTTATCCACTATTCCTTCCTGAACCCGGGC
GAAACTATCACCAGCGAAAAATACTGCCAGCAGATTGACGAAATGCACC
GTAAACTGCAGCGTCTGCAGCCAGCACTGGTGAATCGTAAAGGTCCGAT
CCTGCTGCATGATAACGCCCGTCCGCACGTTGCCCAACCGACCCTGCA
GAAACTGAACGAACTGGGCTATGAAGTTCTGCCACACCCGCCGTACTCC
CCGGATCTGTCCCCGACTGACTACCATTTCTTCAAGCATCTGGACAACTT
CCTGCAGGGTAAACGTTTTCACAACCAACAGGACGCAGAAAACGCTTTC
CAGGAGTTCGTCGAAAGCCGTTCCACTGACTTCTACGCGACCGGTATCA
ACAAGCTGATCAGCCGTTGGCAGAAATGCGTGGACTGTAACGGCAGCTA
CTTCGATTAAGGATCCTCTAGAGTCGACCTGCAGGCAAGCTTGGCACTG
GCCGTCGTTTTACAACGTCGTGACTGGGAAAACCCTGGCGTTACCCAAC
TTAATCGCCTTGCAGCACATCCCCCTTTCGCCAGCTGGCGTAATAGCGA
AGAGGCCCGCACCGATCGCCCTTCCCAACAGTTGCGCAGCCTGAATGG
CGAATGGCAGCTTGGCTGTTTTGGCGGATGAGATAAGATTTTCAGCCTG
ATACAGATTAAATCAGAACGCAGAAGCGGTCTGATAAAACAGAATTTGCC
TGGCGGCAGTAGCGCGGTGGTCCCACCTGACCCCATGCCGAACTCAGA
AGTGAAACGCCGTAGCGCCGATGGTAGTGTGGGGTCTCCCCATGCGAG
AGTAGGGAACTGCCAGGCATCAAATAAAACGAAAGGCTCAGTCGAAAGA
CTGGGCCTTTCGTTTTATCTGTTGTTTGTCGGTGAACGCTCTCCTGAGTA
GGACAAATCCGCCGGGAGCGGATTTGAACGTTGCGAAGCAACGGCCCG
GAGGGTGGCGGGCAGGACGCCCGCCATAAACTGCCAGGCATCAAATTA
AGCAGAAGGCCATCCTGACGGATGGCCTTTTTGCGTTTCTACAAACTCTT
TTTGTTTATTTTTCTAAATACATTCAAATATGTATCCGCTCATGAGACAATA
ACCCTGATAAATGCTTCAATAATATTGAAAAAGGAAGAGTATGAGTATTCA
ACATTTCCGTGTCGCCCTTATTCCCTTTTTTGCGGCATTTTGCCTTCCTGT
TTTTGCTCACCCAGAAACGCTGGTGAAAGTAAAAGATGCTGAAGATCAGT
TGGGTGCACGAGTGGGTTACATCGAACTGGATCTCAACAGCGGTAAGAT
CCTTGAGAGTTTTCGCCCCGAAGAACGTTCTCCAATGATGAGCACTTTTA
AAGTTCTGCTATGTGGCGCGGTATTATCCCGTGTTGACGCCGGGCAAGA
GCAACTCGGTCGCCGCATACACTATTCTCAGAATGACTTGGTTGAGTACT
CACCAGTCACAGAAAAGCATCTTACGGATGGCATGACAGTAAGAGAATT
ATGCAGTGCTGCCATAACCATGAGTGATAACACTGCGGCCAACTTACTTC
TGACAACGATCGGAGGACCGAAGGAGCTAACCGCTTTTTTGCACAACAT
GGGGGATCATGTAACTCGCCTTGATCGTTGGGAACCGGAGCTGAATGAA
GCCATACCAAACGACGAGCGTGACACCACGATGCCTGTAGCAATGGCAA
CAACGTTGCGCAAACTATTAACTGGCGAACTACTTACTCTAGCTTCCCGG
CAACAATTAATAGACTGGATGGAGGCGGATAAAGTTGCAGGACCACTTC
TGCGCTCGGCCCTTCCGGCTGGCTGGTTTATTGCTGATAAATCTGGAGC
CGGTGAGCGTGGGTCTCGCGGTATCATTGCAGCACTGGGGCCAGATGG
TAAGCCCTCCCGTATCGTAGTTATCTACACGACGGGGAGTCAGGCAACT
ATGGATGAACGAAATAGACAGATCGCTGAGATAGGTGCCTCACTGATTA
AGCATTGGTAACTGTCAGACCAAGTTTACTCATATATACTTTAGATTGATT
TACCCCGGTTGATAATCAGAAAAGCCCCAAAAACAGGAAGATTGTATAAG
CAAATATTTAAATTGTAAACGTTAATATTTTGTTAAAATTCGCGTTAAATTT

TTGTTAAATCAGCTCATTTTTTAACCAATAGGCCGAAATCGGCAAAATCC
CTTATAAATCAAAAGAATAGACCGAGATAGGGTTGAGTGTTGTTCCAGTT
TGGAACAAGAGTCCACTATTAAAGAACGTGGACTCCAACGTCAAAGGGC
GAAAAACCGTCTATCAGGGCGATGGCCCACTACGTGAACCATCACCCAA
ATCAAGTTTTTTGGGGTCGAGGTGCCGTAAAGCACTAAATCGGAACCCT
AAAGGGAGCCCCCGATTTAGAGCTTGACGGGGAAAGCCGGCGAACGTG
GCGAGAAAGGAAGGGAAGAAAGCGAAAGGAGCGGGCGCTAGGGCGCT
GGCAAGTGTAGCGGTCACGCTGCGCGTAACCACCACACCCGCCGCGCT
TAATGCGCCGCTACAGGGCGCGTAAAAGGATCTAGGTGAAGATCCTTTT
TGATAATCTCATGACCAAAATCCCTTAACGTGAGTTTTCGTTCCACTGAG
CGTCAGACCCCGTAGAAAAGATCAAAGGATCTTCTTGAGATCCTTTTTTT
CTGCGCGTAATCTGCTGCTTGCAAACAAAAAAACCACCGCTACCAGCGG
TGGTTTGTTTGCCGGATCAAGAGCTACCAACTCTTTTTCCGAAGGTAACT
GGCTTCAGCAGAGCGCAGATACCAAATACTGTCCTTCTAGTGTAGCCGT
AGTTAGGCCACCACTTCAAGAACTCTGTAGCACCGCCTACATACCTCGC
TCTGCTAATCCTGTTACCAGTGGCTGCTGCCAGTGGCGATAAGTCGTGT
CTTACCGGGTTGGACTCAAGACGATAGTTACCGGATAAGGCGCAGCGGT
CGGGCTGAACGGGGGGTTCGTGCACACAGCCCAGCTTGGAGCGAACGA
CCTACACCGAACTGAGATACCTACAGCGTGAGCTATGAGAAAGCGCCAC
GCTTCCCGAAGGGAGAAAGGCGGACAGGTATCCGGTAAGCGGCAGGGT
CGGAACAGGAGAGCGCACGAGGGAGCTTCCAGGGGGAAACGCCTGGT
ATCTTTATAGTCCTGTCGGGTTTCGCCACCTCTGACTTGAGCGTCGATTT
TTGTGATGCTCGTCAGGGGGGCGGAGCCTATGGAAAAACGCCAGCAAC
GCGGCCTTTTTACGGTTCCTGGCCTTTTGCTGGCCTTTTGCTCACATGTT
CTTTCCTGCGTTATCCCCTGATTCTGTGGATAACCGTATTACCGCCTTTG
AGTGAGCTGATACCGCTCGCCGCAGCCGAACGACCGAGCGCAGCGAGT
CAGTGAGCGAGGAAGCGGAAGAGCGCCTGATGCGGTATTTTCTCCTTAC
GCATCTGTGCGGTATTTCACACCGCATATATGGTGCACTCTCAGTACAAT
CTGCTCTGATGCCGCATAGTTAAGCCAGTATACACTCCGCTATCGCTAC
GTGACTGGGTCATGGCTGCGCCCCGACACCCGCCAACACCCGCTGACG
CGCCCTGACGGGCTTGTCTGCTCCCGGCATCCGCTTACAGACAAGCTGT
GACCGTCTCCGGGAGCTGCATGTGTCAGAGGTTTTCACCGTCATCACCG
AAACGCGCGAGGCAGCTGCGGTAAAGCTCATCAGCGTGGTCGTGCAGC
GATTCACAGATGTCTGCCTGTTCATCCGCGTCCAGCTCGTTGAGTTTCTC
CAGAAGCGTTAATGTCTGGCTTCTGATAAAGCGGGCCATGTTAAGGGCG
GTTTTTTCCTGTTTGGTCACTTGATGCCTCCGTGTAAGGGGGAATTTCTG
TTCATGGGGGTAATGATACCGATGAAACGAGAGAGGATGCTCACGATAC
GGGTTACTGATGATGAACATGCCCGGTTACTGGAACGTTGTGAGGGTAA
ACAACTGGCGGTATGGATGCGGCGGGACCAGAGAAAAATCACTCAGGG
TCAATGCCAGCGCTTCGTTAATACAGATGTAGGTGTTCCACAGGGTAGC
CAGCAGCATCCTGCGATGCAGATCCGGAACATAATGGTGCAGGGCGCT
GACTTCCGCGTTTCCAGACTTTACGAAACACGGAAACCGAAGACCATTC
ATGTTGTTGCTCAGGTCGCAGACGTTTTGCAGCAGCAGTCGCTTCACGT
TCGCTCGCGTATCGGTGATTCATTCTGCTAACCAGTAAGGCAACCCCGC
CAGCCTAGCCGGGTCCTCAACGACAGGAGCACGATCATGCGCACCCGT
GGCCAGGACCCAACGCTGCCCGAAATT

pRC2102

GGGTGCTTTGCCAAGGGTACCAATGTTTTAATGGCGGATGGGTCTATTG
AATGTATTGAAAACATTGAGGTTGGTAATAAGGTCATGGGTAAAGATGGC
AGACCTCGTGAGGTAATTAAATTGCCCAGAGGAAGAGAAACTATGTACA
GCGTCGTGCAGAAAAGTCAGCACAGAGCCCACAAAAGTGACTCAAGTCG
TGAAGTGCCAGAATTACTCAAGTTTACGTGTAATGCGACCCATGAGTTGG
TTGTTAGAACACCTCGTAGTGTCCGCCGTTTGTCTCGTACCATTAAGGGT
GTCGAATATTTTGAAGTTATTACTTTTGAGATGGGCCAAAAGAAAGCCCC
CGACGGTAGAATTGTTGAGCTTGTCAAGGAAGTTTCAAAGAGCTACCCA
ATATCTGAGGGGCCTGAGAGAGCCAACGAATTAGTAGAATCCTATAGAA
AGGCTTCAAATAAAGCTTATTTTGAGTGGACTATTGAGGCCAGAGATCTT
TCTCTGTTGGGTTCCCATGTTCGTAAAGCTACCTACCAGACTTACGCTCC
AATTCTTTATGAGAATGACCACTTTTTCGACTACATGCAAAAAAGTAAGTT
TCATCTCACCATTGAAGGTCCAAAAGTACTTGCTTATTTACTTGGTTTATG
GATTGGTGATGGATTGTCTGACAGGGCAACTTTTTCGGTTGATTCCAGAG
ATACTTCTTTGATGGAACGTGTTACTGAATATGCTGAAAAGTTGAATTTGT
GCGCCGAGTATAAGGACAGAAAAGAACCACAAGTTGCCAAAACTGTTAA
TTTGTACTCTAAAGTTGTCAGAGGTAATGGTATTCGCAATAATCTTAATAC
TGAGAATCCATTATGGGACGCTATTGTTGGCTTAGGATTCTTGAAGGACG
GTGTCAAAAATATTCCTTCTTTCTTGTCTACGGACAATATCGGTACTCGTG
AAACATTTCTTGCTGGTCTAATTGATTCTGATGGCTATGTTACTGATGAGC
ATGGTATTAAAGCAACAATAAAGACAATTCATACTTCTGTCAGAGATGGTT
TGGTTTCCCTTGCTCGTTCTTTAGGCTTAGTAGTCTCGGTTAACGCAGAA
CCTGCTAAGGTTGACATGAATGTCACCAAACATAAAATTAGTTATGCTATT
TATATGTCTGGTGGAGATGTTTTGCTTAACGTTCTTTCGAAGTGTGCCGG
CTCTAAAAAATTCAGGCCTGCTCCCGCCGCTGCTTTTGCACGTGAGTGC
CGCGGATTTTATTTCGAGTTACAAGAATTGAAGGAAGACGATTATTATGG
GATTACTTTATCTGATGATTCTGATCATCAGTTTTTGCTTGGATCCCAGGT
TGTCGTCCATGCATGCGGTGGCCTGACCGGTCTGAACTCAGGCCTCAC
GACAAATCCTGGTGTATCCGCTTGGCAGGTCAACACAGCTTATACTGCG
GGACAATTGGTCACATATAACGGCAAGACGTATAAATGTTTGCAGCCCCA
CACCTCCTTGGCAGGATGGGAACCATCCAACGTTCCTGCCTTGTGGCAG
CTTCAATGACTGCAGGAAGGGGATCCGGCTGCTAACAAAGCCCGAAAG
GAAGCTGAGTTGGCTGCTGCCACCGCTGAGCAATAACTAGCATAACCCC
TTGGGGCCTCTAAACGGGTCTTGAGGGGTTTTTTGCTGAAAGGAGGAAC
TATATCCGGATAACTACGTCAGGTGGCACTTTTCGGGGAAATGTGCGCG
GAACCCCTATTTGTTTATTTTTCTAAATACATTCAAATATGTATCCGCTCAT
GAGACAATAACCCTGATAAATGCTTCAATAATATTGAAAAAGGAAGAGTA
TGAGTATTCAACATTTCCGTGTCGCCCTTATTCCCTTTTTTGCGGCATTTT
GCCTTCCTGTTTTTGCTCACCCAGAAACGCTGGTGAAAGTAAAAGATGCT
GAAGATCAGTTGGGTGCACGAGTGGGTTACATCGAACTGGATCTCAACA
GCGGTAAGATCCTTGAGAGTTTTCGCCCCGAAGAACGTTCTCCAATGAT
GAGCACTTTTAAAGTTCTGCTATGTGGCGCGGTATTATCCCGTGTTGACG
CCGGGCAAGAGCAACTCGGTCGCCGCATACACTATTCTCAGAATGACTT
GGTTGAGTACTCACCAGTCACAGAAAAGCATCTTACGGATGGCATGACA
GTAAGAGAATTATGCAGTGCTGCCATAACCATGAGTGATAACACTGCGG
CCAACTTACTTCTGACAACGATCGGAGGACCGAAGGAGCTAACCGCTTT
TTTGCACAACATGGGGGATCATGTAACTCGCCTTGATCGTTGGGAACCG
GAGCTGAATGAAGCCATACCAAACGACGAGCGTGACACCACGATGCCTG
TAGCAATGGCAACAACGTTGCGCAAACTATTAACTGGCGAACTACTTACT
CTAGCTTCCCGGCAACAATTAATAGACTGGATGGAGGCGGATAAAGTTG

CAGGACCACTTCTGCGCTCGGCCCTTCCGGCTGGCTGGTTTATTGCTGA
TAAATCTGGAGCCGGTGAGCGTGGGTCTCGCGGTATCATTGCAGCACTG
GGGCCAGATGGTAAGCCCTCCCGTATCGTAGTTATCTACACGACGGGGA
GTCAGGCAACTATGGATGAACGAAATAGACAGATCGCTGAGATAGGTGC
CTCACTGATTAAGCATTGGTAACTGTCAGACCAAGTTTACTCATATATACT
TTAGATTGATTTACCCCGGTTGATAATCAGAAAAGCCCCAAAAACAGGAA
GATTGTATAAGCAAATATTTAAATTGTAAACGTTAATATTTTGTTAAAATTC
GCGTTAAATTTTTGTTAAATCAGCTCATTTTTTAACCAATAGGCCGAAATC
GGCAAAATCCCTTATAAATCAAAAGAATAGCCCGAGATAGGGTTGAGTGT
TGTTCCAGTTTGGAACAAGAGTCCACTATTAAAGAACGTGGACTCCAACG
TCAAAGGGCGAAAAACCGTCTATCAGGGCGATGGCCCACTACGTGAACC
ATCACCCAAATCAAGTTTTTTGGGGTCGAGGTGCCGTAAAGCACTAAATC
GGAACCCTAAAGGGAGCCCCCGATTTAGAGCTTGACGGGGAAAGCCGG
CGAACGTGGCGAGAAAGGAAGGGAAGAAAGCGAAAGGAGCGGGCGCT
AGGGCGCTGGCAAGTGTAGCGGTCACGCTGCGCGTAACCACCACACCC
GCCGCGCTTAATGCGCCGCTACAGGGCGCGTAAAAGGATCTAGGTGAA
GATCCTTTTTGATAATCTCATGACCAAAATCCCTTAACGTGAGTTTTCGTT
CCACTGAGCGTCAGACCCCGTAGAAAAGATCAAAGGATCTTCTTGAGAT
CCTTTTTTTCTGCGCGTAATCTGCTGCTTGCAAACAAAAAAACCACCGCT
ACCAGCGGTGGTTTGTTTGCCGGATCAAGAGCTACCAACTCTTTTTCCGA
AGGTAACTGGCTTCAGCAGAGCGCAGATACCAAATACTGTCCTTCTAGT
GTAGCCGTAGTTAGGCCACCACTTCAAGAACTCTGTAGCACCGCCTACA
TACCTCGCTCTGCTAATCCTGTTACCAGTGGCTGCTGCCAGTGGCGATA
AGTCGTGTCTTACCGGGTTGGACTCAAGACGATAGTTACCGGATAAGGC
GCAGCGGTCGGGCTGAACGGGGGGTTCGTGCACACAGCCCAGCTTGGA
GCGAACGACCTACACCGAACTGAGATACCTACAGCGTGAGCTATGAGAA
AGCGCCACGCTTCCCGAAGGGAGAAAGGCGGACAGGTATCCGGTAAGC
GGCAGGGTCGGAACAGGAGAGCGCACGAGGGAGCTTCCAGGGGGAAA
CGCCTGGTATCTTTATAGTCCTGTCGGGTTTCGCCACCTCTGACTTGAGC
GTCGATTTTTGTGATGCTCGTCAGGGGGGCGGAGCCTATGGAAAAACGC
CAGCAACGCGGCCTTTTTACGGTTCCTGGCCTTTTGCTGGCCTTTTGCTC
ACATGTTCTTTCCTGCGTTATCCCCTGATTCTGTGGATAACCGTATTACC
GCCTTTGAGTGAGCTGATACCGCTCGCCGCAGCCGAACGACCGAGCGC
AGCGAGTCAGTGAGCGAGGAAGCTATGGTGCACTCTCAGTACAATCTGC
TCTGATGCCGCATAGTTAAGCCAGTATACACTCCGCTATCGCTACGTGAC
TGGGTCATGGCTGCGCCCCGACACCCGCCAACACCCGCTGACGCGCCC
TGACGGGCTTGTCTGCTCCCGGCATCCGCTTACAGACAAGCTGTGACCG
TCTCCGGGAGCTGCATGTGTCAGAGGTTTTCACCGTCATCACCGAAACG
CGCGAGGCAGCTGCGGTAAAGCTCATCAGCGTGGTCGTGCAGCGATTC
ACAGATGTCTGCCTGTTCATCCGCGTCCAGCTCGTTGAGTTTCTCCAGAA
GCGTTAATGTCTGGCTTCTGATAAAGCGGGCCATGTTAAGGGCGGTTTT
TTCCTGTTTGGTCACTGATGCCTCCGTGTAAGGGGGATTTCTGTTCATGG
GGGTAATGATACCGATGAAACGAGAGAGGATGCTCACGATACGGGTTAC
TGATGATGAACATGCCCGGTTACTGGAACGTTGTGAGGGTAAACAACTG
GCGGTATGGATGCGGCGGGACCAGAGAAAAATCACTCAGGGTCAATGC
CAGCCGAACGCCAGCAAGACGTAGCCCAGCGCGTCGGCCGCCATGCC
GGCGATAATGGCCTGCTTCTCGCCGAAACGTTTGGTGGCGGGACCAGT
GACGAAGGCTTGAGCGAGGGCGTGCAAGATTCCGAATACCGCAAGCGA
CAGGCCGATCATCGTCGCGCTCCAGCGAAAGCGGTCCTCGCCGAAAAT
GACCCAGAGCGCTGCCGGCACCTGTCCTACGAGTTGCATGATAAAGAAG

ACAGTCATAAGTGCGGCGACGATAGTCATGCCCCGCGCCCACCGGAAG
GAGCTGACTGGGTTGAAGGCTCTCAAGGGCATCGGTCGAGATCCCGGT
GCCTAATGAGTGAGCTAACTTACATTAATTGCGTTGCGCTCACTGCCCGC
TTTCCAGTCGGGAAACCTGTCGTGCCAGCTGCATTAATGAATCGGCCAA
CGCGCGGGGAGAGGCGGTTTGCGTATTGGGCGCCAGGGTGGTTTTTCT
TTTCACCAGTGAGACGGGCAACAGCTGATTGCCCTTCACCGCCTGGCCC
TGAGAGAGTTGCAGCAAGCGGTCCACGCTGGTTTGCCCCAGCAGGCGA
AAATCCTGTTTGATGGTGGTTAACGGCGGGATATAACATGAGCTGTCTTC
GGTATCGTCGTATCCCACTACCGAGATATCCGCACCAACGCGCAGCCCG
GACTCGGTAATGGCGCGCATTGCGCCCAGCGCCATCTGATCGTTGGCA
ACCAGCATCGCAGTGGGAACGATGCCCTCATTCAGCATTTGCATGGTTT
GTTGAAAACCGGACATGGCACTCCAGTCGCCTTCCCGTTCCGCTATCGG
CTGAATTTGATTGCGAGTGAGATATTTATGCCAGCCAGCCAGACGCAGA
CGCGCCGAGACAGAACTTAATGGGCCCGCTAACAGCGCGATTTGCTGG
TGACCCAATGCGACCAGATGCTCCACGCCCAGTCGCGTACCGTCTTCAT
GGGAGAAAATAATACTGTTGATGGGTGTCTGGTCAGAGACATCAAGAAA
TAACGCCGGAACATTAGTGCAGGCAGCTTCCACAGCAATGGCATCCTGG
TCATCCAGCGGATAGTTAATGATCAGCCCACTGACGCGTTGCGCGAGAA
GATTGTGCACCGCCGCTTTACAGGCTTCGACGCCGCTTCGTTCTACCAT
CGACACCACCACGCTGGCACCCAGTTGATCGGCGCGAGATTTAATCGCC
GCGACAATTTGCGACGGCGCGTGCAGGGCCAGACTGGAGGTGGCAACG
CCAATCAGCAACGACTGTTTGCCCGCCAGTTGTTGTGCCACGCGGTTGG
GAATGTAATTCAGCTCCGCCATCGCCGCTTCCACTTTTTCCCGCGTTTTC
GCAGAAACGTGGCTGGCCTGGTTCACCACGCGGGAAACGGTCTGATAA
GAGACACCGGCATACTCTGCGACATCGTATAACGTTACTGGTTTCACATT
CACCACCCTGAATTGACTCTCTTCCGGGCGCTATCATGCCATACCGCGA
AAGGTTTTGCGCCATTCGATGGTGTCCGGGATCTCGACGCTCTCCCTTA
TGCGACTCCTGCATTAGGAAGCAGCCCAGTAGTAGGTTGAGGCCGTTGA
GCACCGCCGCCGCAAGGAATGGTGCATGCCGGCATGCCGCCCTTTCGT
CTTCAAGAATTAATTCCCAATTCCCCAGGCATCAAATAAAACGAAAGGCT
CAGTCGAAAGACTGGGCCTTTCGTTTTATCTGTTGTTTGTCGGTGAACGC
TCTCCTGAGTAGGACAAATCCGCCGGGAGCGGATTTGAACGTTGCGAAG
CAACGGCCCGGAGGGTGGCGGGCAGGACGCCCGCCATAAACTGCCAG
GAATTAATTCCCCAGGCATCAAATAAAACGAAAGGCTCAGTCGAAAGACT
GGGCCTTTCGTTTTATCTGTTGTTTGTCGGTGAACGCTCTCCTGAGTAGG
ACAAATCCGCCGGGAGCGGATTTGAACGTTGCGAAGCAACGGCCCGGA
GGGTGGCGGGCAGGACGCCCGCCATAAACTGCCAGGAATTAATTCCCC
AGGCATCAAATAAAACGAAAGGCTCAGTCGAAAGACTGGGCCTTTCGTT
TTATCTGTTGTTTGTCGGTGAACGCTCTCCTGAGTAGGACAAATCCGCCG
GGAGCGGATTTGAACGTTGCGAAGCAACGGCCCGGAGGGTGGCGGGC
AGGACGCCCGCCATAAACTGCCAGGAATTAATTCCCCAGGCATCAAATA
AAACGAAAGGCTCAGTCGAAAGACTGGGCCTTTCGTTTTATCTGTTGTTT
GTCGGTGAACGCTCTCCTGAGTAGGACAAATCCGCCGGGAGCGGATTT
GAACGTTGCGAAGCAACGGCCCGGAGGGTGGCGGGCAGGACGCCCGC
CATAAACTGCCAGGAATTAATTCCCCAGGCATCAAATAAAACGAAAGGCT
CAGTCGAAAGACTGGGCCTTTCGTTTTATCTGTTGTTTGTCGGTGAACGC
TCTCCTGAGTAGGACAAATCCGCCGGGAGCGGATTTGAACGTTGCGAAG
CAACGGCCCGGAGGGTGGCGGGCAGGACGCCCGCCATAAACTGCCAG
GAATTGGGGATCGGAATTAATTCCCGGTTTAAACCGGGGATCTCGATCC
CGCGAAATTAATACGACTCACTATAGGGGAATTGTGAGCGGATAACAATT

CCCCTCTAGAAATAATTTTGTTTAACTTTAAGAAGGAGATATACATATGGC
TAGCTCGCGAATGATAACTTCTGCTCTTCATCGTGCGGCCGACTGGGCT
AAATCTGTGTTCTCTTCGGCGGCGCTGGGTGATCCTCGCCGTACTGCCC
GCTTGGTTAACGTCGCCGCCCAATTGGCAAAATATTCTGGTAAATCAATA
ACCATCTCATCAGAGGGTAGTAAGGCCGCACAGGAAGGCGCTTACCGAT
TTATCCGCAATCCCAACGTTTCTGCCGAGGCGATCAGAAAGGCTGGCGC
CATGCAAACAGTCAAGTTGGCTCAGGAGTTTCCCGAACTGCTGGCCATT
GAGGACACCACCTCTTTGAGTTATCGCCACCAGGTCGCCGAAGAGCTTG
GCAAGCTGGGCTCTATTCAGGATAAATCCCGCGGATGGTGGGTTCACTC
CGTTCTCTTGCTCGAGGCCACCACATTCCGCACCGTAGGATTACTGCAT
CAGGAGTGGTGGATGCGCCCGGATGACCCTGCCGATGCGGATGAAAAG
GAGAGTGGCAAATGGCTGGCAGCGGCCGCAACTAGCCGGTTACGCATG
GGCAGCATGATGAGCAACGTGATTGCGGTCTGTGACCGCGAAGCCGAT
ATTCATGCTTATCTGCAGGACAAACTGGCGCATAACGAGCGCTTCGTGG
TGCGCTCCAAGCACCCACGCAAGGACGTAGAGTCTGGGTTGTATCTGTA
CGACCATCTGAAGAACCAACCGGAGTTGGGTGGCTATCAGATCAGCATT
CCGCAAAAGGGCGTGGTGGATAAACGCGGTAAACGTAAAAATCGACCAG
CCCGCAAGGCGAGCTTGAGCCTGCGCAGTGGGCGCATCACGCTAAAAC
AGGGGAATATCACGCTCAACGCGGTGCTGGCCGAGGAGATTAACCCGC
CCAAGGGTGAGACCCCGTTGAAATGGTTGTTGCTGACCAGCGAACCGGT
CGAGTCGCTAGCCCAAGCCTTGCGCGTCATCGACATTTATACCCATCGC
TGGCGGATCGAGGAGTTCCATAAGGCATGGAAAACCGGAGCAGGAGCC
GAGAGGCAACGCATGGAGGAGCCGGATAATCTGGAGCGGATGGTCTCG
ATCCTCTCGTTTGTTGCGGTCAGGCTGTTACAGCTCAGAGAAAGCTTCAC
GCCGCCGCAAGCACTCAGGGCGCAAGGGCTGCTAAAGGAAGCGGAACA
CGTAGAAAGCCAGTCCGCAGAAACGGTGCTGACCCCGGATGAATGTCA
GCTACTGGGCTATCTGGACAAGGGAAAACGCAAGCGCAAAGAGAAAGC
AGGTAGCTTGCAGTGGGCTTACATGGCGATAGCTAGACTGGGCGGTTTT
ATGGACAGCAAGCGAACCGGAATTGCCAGCTGGGGCGCCCTCTGGGAA
GGTTGGGAAGCCCTGCAAAGTAAACTGGATGGCTTTCTTGCCGCCAAGG
ATCTGATGGCGCAGGGGATCAAGATCGGATCCTCTAGAGGAGGTGGCT
CAGAAGGTGGCGGATCTGAAGGTGGCTCTGGAACTAGTGAATTCATGAT
AACTTCTGCTCTTCATCGTGCGGCCGACTGGGCTAAATCTGTGTTCTCTT
CGGCGGCGCTGGGTGATCCTCGCCGTACTGCCCGCTTGGTTAACGTCG
CCGCCCAATTGGCAAAATATTCTGGTAAATCAATAACCATCTCATCAGAG
GGTAGTAAGGCCGCACAGGAAGGCGCTTACCGATTTATCCGCAATCCCA
ACGTTTCTGCCGAGGCGATCAGAAAGGCTGGCGCCATGCAAACAGTCAA
GTTGGCTCAGGAGTTTCCCGAACTGCTGGCCATTGAGGACACCACCTCT
TTGAGTTATCGCCACCAGGTCGCCGAAGAGCTTGGCAAGCTGGGCTCTA
TTCAGGATAAATCCCGCGGATGGTGGGTTCACTCCGTTCTCTTGCTCGA
GGCCACCACATTCCGCACCGTAGGATTACTGCATCAGGAGTGGTGGATG
CGCCCGGATGACCCTGCCGATGCGGATGAAAAGGAGAGTGGCAAATGG
CTGGCAGCGGCCGCAACTAGCCGGTTACGCATGGGCAGCATGATGAGC
AACGTGATTGCGGTCTGTGACCGCGAAGCCGATATTCATGCTTATCTGC
AGGACAAACTGGCGCATAACGAGCGCTTCGTGGTGCGCTCCAAGCACC
CACGCAAGGACGTAGAGTCTGGGTTGTATCTGTACGACCATCTGAAGAA
CCAACCGGAGTTGGGTGGCTATCAGATCAGCATTCCGCAAAAGGGCGT
GGTGGATAAACGCGGTAAACGTAAAAATCGACCAGCCCGCAAGGCGAG
CTTGAGCCTGCGCAGTGGGCGCATCACGCTAAAACAGGGGAATATCAC
GCTCAACGCGGTGCTGGCCGAGGAGATTAACCCGCCCAAGGGTGAGAC

CCCGTTGAAATGGTTGTTGCTGACCAGCGAACCGGTCGAGTCGCTAGCC
CAAGCCTTGCGCGTCATCGACATTTATACCCATCGCTGGCGGATCGAGG
AGTTCCATAAGGCATGGAAAACCGGAGCAGGAGCCGAGAGGCAACGCA
TGGAGGAGCCGGATAATCTGGAGCGGATGGTCTCGATCCTCTCGTTTGT
TGCGGTCAGGCTGTTACAGCTCAGAGAAAGCTTCACGCCGCCGCAAGC
ACTCAGGGCGCAAGGGCTGCTAAAGGAAGCGGAACACGTAGAAAGCCA
GTCCGCAGAAACGGTGCTGACCCCGGATGAATGTCAGCTACTGGGCTAT
CTGGACAAGGGAAAACGCAAGCGCAAAGAGAAAGCAGGTAGCTTGCAG
TGGGCTTACATGGCGATAGCTAGACTGGGCGGTTTTATGGACAGCAAGC
GAACCGGAATTGCCAGCTGGGGCGCCCTCTGGGAAGGTTGGGAAGCCC
TGCAAAGTAAACTGGATGGCTTTCTTGCCGCCAAGGATCTGATGGCGCA
GGGGATCAAGATCCCC

pRC2106
TCGCGCGTTTCGGTGATGACGGTGAAAACCTCTGACACATGCAGCTCCC
GGAGACGGTCACAGCTTGTCTGTAAGCGGATGCCGGGAGCAGACAAGC
CCGTCAGGGCGCGTCAGCGGGTGTTGGCGGGTGTCGGGGCTGGCTTA
ACTATGCGGCATCAGAGCAGATTGTACTGAGAGTGCACCATATGCGGTG
TGAAATACCGCACAGATGCGTAAGGAGAAAATACCGCATCAGGCGCCAT
TCGCCATTCAGGCTGCGCAACTGTTGGGAAGGGCGATCGGTGCGGGCC
TCTTCGCTATTACGCCAGCTGGCGAAAGGGGGATGTGCTGCAAGGCGAT
TAAGTTGGGTAACGCCAGGGTTTTCCCAGTCACGACGTTGTAAAACGAC
GGCCAGTGAATTCCTGTCTCTTATACACATCTTGCATTGTTGGAATTTGC
CGTTTGATATTGGAATACATTCTTAAATAAATGTGGTTATGTTATACATCA
TTTTAATGCGCATTTCTCGCTTTACGTTTTTTTGCTAATGACTTATTACTTG
CTGTTTATTTTATGTTTATTTTAGACTGGTACCCGATCCAGACATGATAAG
ATACATTGATGAGTTTGGACAAACCACAACTAGAATGCAGTGAAAAAAAT
GCTTTATTTGTGAAATTTGTGATGCTATTGCTTTATTTGTAACCATTATAAG
CTGCAATAAACAAGTTGGGGTGGGCGAAGAACTCCAGCATGAGATCCCC
GCGCTGGAGGATCATCCAGCCGGCGTCCCGGAAAACGATTCCGAAGCC
CAACCTTTCATAGAAGGCGGCGGTGGAATCGAAATCTCGTGATGGCAGG
TTGGGCGTCGCTTGGTCGGTCATTTCGAACCCCAGAGTCCCGCTCAGAA
GAACTCGTCAAGAAGGCGATAGAAGGCGATGCGCTGCGAATCGGGAGC
GGCGATACCGTAAAGCACGAGGAAGCGGTCAGCCCATTCGCCGCCAAG
CTCTTCAGCAATATCACGGGTAGCCAACGCTATGTCCTGATAGCGGTCC
GCCACACCCAGCCGGCCACAGTCGATGAATCCAGAAAAGCGGCCATTTT
CCACCATGATATTCGGCAAGCAGGCATCGCCATGGGTCACGACGAGATC
CTCGCCGTCGGGCATGCGCGCCTTGAGCCTGGCGAACAGTTCGGCTGG
CGCGAGCCCCTGATGCTCTTCGTCCAGATCATCCTGATCGACAAGACCG
GCTTCCATCCGAGTACGTGCTCGCTCGATGCGATGTTTCGCTTGGTGGT
CGAATGGGCAGGTAGCCGGATCAAGCGTATGCAGCCGCCGCATTGCAT
CAGCCATGATGGATACTTTCTCGGCAGGAGCAAGGTGAGATGACAGGAG
ATCCTGCCCCGGCACTTCGCCCAATAGCAGCCAGTCCCTTCCCGCTTCA
GTGACAACGTCGAGCACAGCTGCGCAAGGAACGCCCGTCGTGGCCAGC
CACGATAGCCGCGCTGCCTCGTCCTGCAGTTCATTCAGGGCACCGGAC
AGGTCGGTCTTGACAAAAAGAACCGGGCGCCCCTGCGCTGACAGCCGG
AACACGGCGGCATCAGAGCAGCCGATTGTCTGTTGTGCCCAGTCATAGC
CGAATAGCCTCTCCACCCAAGCGGCCGGAGAACCTGCGTGCAATCCATC
TTGTTCAATCATGCGAAACGATCCTCATCCTGTCTCTTGATCAGATCCGA
AAATGGATATCCAAGCTCCCGGGAGCTTTTTGCAAAAGCCTAGGCCTCC

AAAAAAGCCTCCTCACTACTTCTGGAATAGCTCAGAGGCCGAGGCGGCC
TCGGCCTCTGCATAAATAAAAAAAATTAGTCAGCCATGGGGCGGAGAAT
GGGCGGAACTGGGCGGAGTTAGGGGCGGGATGGGCGGAGTTAGGGGC
GGGACTATGGTTGCTGACTAATTGAGATGCATGCTTTGCATACTTCTGCC
TGCTGGGGAGCCTGGGGACTTTCCACACCTGGTTGCTGACTAATTGAGA
TGCATGCTTTGCATACTTCTGCCTGCCTGGGGAGCCTGGGGACTTTCCA
CACCCTAACTGACACACATTCCACAGAATTGGGGATCCTCTAGATAAAGA
TGTGTTTGAGCCTAGTTATAATGATTTAAAATTCACGGTCCAGATGTGTAT
AAGAGACAGAAGCTTGGCGTAATCATGGTCATAGCTGTTTCCTGTGTGAA
ATTGTTATCCGCTCACAATTCCACACAACATACGAGCCGGAAGCATAAAG
TGTAAAGCCTGGGGTGCCTAATGAGTGAGCTAACTCACATTAATTGCGTT
GCGCTCACTGCCCGCTTTCCAGTCGGGAAACCTGTCGTGCCAGCTGCAT
TAATGAATCGGCCAACGCGCGGGGAGAGGCGGTTTGCGTATTGGGCGC
TCTTCCGCTTCCTCGCTCACTGACTCGCTGCGCTCGGTCGTTCGGCTGC
GGCGAGCGGTATCAGCTCACTCAAAGGCGGTAATACGGTTATCCACAGA
ATCAGGGGATAACGCAGGAAAGAACATGTGAGCAAAAGGCCAGCAAAAG
GCCAGGAACCGTAAAAAGGCCGCGTTGCTGGCGTTTTTCCATAGGCTCC
GCCCCCCTGACGAGCATCACAAAAATCGACGCTCAAGTCAGAGGTGGC
GAAACCCGACAGGACTATAAAGATACCAGGCGTTTCCCCCTGGAAGCTC
CCTCGTGCGCTCTCCTGTTCCGACCCTGCCGCTTACCGGATACCTGTCC
GCCTTTCTCCCTTCGGGAAGCGTGGCGCTTTCTCATAGCTCACGCTGTA
GGTATCTCAGTTCGGTGTAGGTCGTTCGCTCCAAGCTGGGCTGTGTGCA
CGAACCCCCCGTTCAGCCCGACCGCTGCGCCTTATCCGGTAACTATCGT
CTTGAGTCCAACCCGGTAAGACACGACTTATCGCCACTGGCAGCAGCCA
CTGGTAACAGGATTAGCAGAGCGAGGTATGTAGGCGGTGCTACAGAGTT
CTTGAAGTGGTGGCCTAACTACGGCTACACTAGAAGGACAGTATTTGGT
ATCTGCGCTCTGCTGAAGCCAGTTACCTTCGGAAAAAGAGTTGGTAGCT
CTTGATCCGGCAAACAAACCACCGCTGGTAGCGGTGGTTTTTTTGTTTGC
AAGCAGCAGATTACGCGCAGAAAAAAAGGATCTCAAGAAGATCCTTTGAT
CTTTTCTACGGGGTCTGACGCTCAGTGGAACGAAAACTCACGTTAAGGG
ATTTTGGTCATGAGATTATCAAAAAGGATCTTCACCTAGATCCTTTTAAAT
TAAAAATGAAGTTTTAAATCAATCTAAAGTATATATGAGTAAACTTGGTCT
GACAGTTACCAATGCTTAATCAGTGAGGCACCTATCTCAGCGATCTGTCT
ATTTCGTTCATCCATAGTTGCCTGACTCCCCGTCGTGTAGATAACTACGA
TACGGGAGGGCTTACCATCTGGCCCCAGTGCTGCAATGATACCGCGAG
ACCCACGCTCACCGGCTCCAGATTTATCAGCAATAAACCAGCCAGCCGG
AAGGGCCGAGCGCAGAAGTGGTCCTGCAACTTTATCCGCCTCCATCCAG
TCTATTAATTGTTGCCGGGAAGCTAGAGTAAGTAGTTCGCCAGTTAATAG
TTTGCGCAACGTTGTTGCCATTGCTACAGGCATCGTGGTGTCACGCTCG
TCGTTTGGTATGGCTTCATTCAGCTCCGGTTCCCAACGATCAAGGCGAG
TTACATGATCCCCCATGTTGTGCAAAAAAGCGGTTAGCTCCTTCGGTCCT
CCGATCGTTGTCAGAAGTAAGTTGGCCGCAGTGTTATCACTCATGGTTAT
GGCAGCACTGCATAATTCTCTTACTGTCATGCCATCCGTAAGATGCTTTT
CTGTGACTGGTGAGTACTCAACCAAGTCATTCTGAGAATAGTGTATGCG
GCGACCGAGTTGCTCTTGCCCGGCGTCAATACGGGATAATACCGCGCC
ACATAGCAGAACTTTAAAAGTGCTCATCATTGGAAAACGTTCTTCGGGGC
GAAAACTCTCAAGGATCTTACCGCTGTTGAGATCCAGTTCGATGTAACCC
ACTCGTGCACCCAACTGATCTTCAGCATCTTTTACTTTCACCAGCGTTTC
TGGGTGAGCAAAAACAGGAAGGCAAAATGCCGCAAAAAAGGGAATAAGG
GCGACACGGAAATGTTGAATACTCATACTCTTCCTTTTTCAATATTATTGA

AGCATTTATCAGGGTTATTGTCTCATGAGCGGATACATATTTGAATGTATT
TAGAAAAATAAACAAATAGGGGTTCCGCGCACATTTCCCCGAAAAGTGC
CACCTGACGTCTAAGAAACCATTATTATCATGACATTAACCTATAAAAATA
GGCGTATCACGAGGCCCTTTCGTC

pRC2107
GGGTGCTTTGCCAAGGGTACCAATGTTTTAATGGCGGATGGGTCTATTG
AATGTATTGAAAACATTGAGGTTGGTAATAAGGTCATGGGTAAAGATGGC
AGACCTCGTGAGGTAATTAAATTGCCCAGAGGAAGAGAAACTATGTACA
GCGTCGTGCAGAAAAGTCAGCACAGAGCCCACAAAAGTGACTCAAGTCG
TGAAGTGCCAGAATTACTCAAGTTTACGTGTAATGCGACCCATGAGTTGG
TTGTTAGAACACCTCGTAGTGTCCGCCGTTTGTCTCGTACCATTAAGGGT
GTCGAATATTTTGAAGTTATTACTTTTGAGATGGGCCAAAAGAAAGCCCC
CGACGGTAGAATTGTTGAGCTTGTCAAGGAAGTTTCAAAGAGCTACCCA
ATATCTGAGGGGCCTGAGAGAGCCAACGAATTAGTAGAATCCTATAGAA
AGGCTTCAAATAAAGCTTATTTTGAGTGGACTATTGAGGCCAGAGATCTT
TCTCTGTTGGGTTCCCATGTTCGTAAAGCTACCTACCAGACTTACGCTCC
AATTCTTTATGAGAATGACCACTTTTTCGACTACATGCAAAAAAGTAAGTT
TCATCTCACCATTGAAGGTCCAAAAGTACTTGCTTATTTACTTGGTTTATG
GATTGGTGATGGATTGTCTGACAGGGCAACTTTTTCGGTTGATTCCAGAG
ATACTTCTTTGATGGAACGTGTTACTGAATATGCTGAAAAGTTGAATTTGT
GCGCCGAGTATAAGGACAGAAAAGAACCACAAGTTGCCAAAACTGTTAA
TTTGTACTCTAAAGTTGTCAGAGGTAATGGTATTCGCAATAATCTTAATAC
TGAGAATCCATTATGGGACGCTATTGTTGGCTTAGGATTCTTGAAGGACG
GTGTCAAAAATATTCCTTCTTTCTTGTCTACGGACAATATCGGTACTCGTG
AAACATTTCTTGCTGGTCTAATTGATTCTGATGGCTATGTTACTGATGAGC
ATGGTATTAAAGCAACAATAAAGACAATTCATACTTCTGTCAGAGATGGTT
TGGTTTCCCTTGCTCGTTCTTTAGGCTTAGTAGTCTCGGTTAACGCAGAA
CCTGCTAAGGTTGACATGAATGTCACCAAACATAAAATTAGTTATGCTATT
TATATGTCTGGTGGAGATGTTTTGCTTAACGTTCTTTCGAAGTGTGCCGG
CTCTAAAAAATTCAGGCCTGCTCCCGCCGCTGCTTTTGCACGTGAGTGC
CGCGGATTTTATTTCGAGTTACAAGAATTGAAGGAAGACGATTATTATGG
GATTACTTTATCTGATGATTCTGATCATCAGTTTTTGCTTGGATCCCAGGT
TGTCGTCCATGCATGCGGTGGCCTGACCGGTCTGAACTCAGGCCTCAC
GACAAATCCTGGTGTATCCGCTTGGCAGGTCAACACAGCTTATACTGCG
GGACAATTGGTCACATATAACGGCAAGACGTATAAATGTTTGCAGCCCCA
CACCTCCTTGGCAGGATGGGAACCATCCAACGTTCCTGCCTTGTGGCAG
CTTCAATGACTGCAGGAAGGGGATCCGGCTGCTAACAAAGCCCGAAAG
GAAGCTGAGTTGGCTGCTGCCACCGCTGAGCAATAACTAGCATAACCCC
TTGGGGCCTCTAAACGGGTCTTGAGGGGTTTTTTGCTGAAAGGAGGAAC
TATATCCGGATAACTACGTCAGGTGGCACTTTTCGGGGAAATGTGCGCG
GAACCCCTATTTGTTTATTTTTCTAAATACATTCAAATATGTATCCGCTCAT
GAGACAATAACCCTGATAAATGCTTCAATAATATTGAAAAAGGAAGAGTA
TGAGTATTCAACATTTCCGTGTCGCCCTTATTCCCTTTTTTGCGGCATTTT
GCCTTCCTGTTTTTGCTCACCCAGAAACGCTGGTGAAAGTAAAAGATGCT
GAAGATCAGTTGGGTGCACGAGTGGGTTACATCGAACTGGATCTCAACA
GCGGTAAGATCCTTGAGAGTTTTCGCCCCGAAGAACGTTCTCCAATGAT
GAGCACTTTTAAAGTTCTGCTATGTGGCGCGGTATTATCCCGTGTTGACG
CCGGGCAAGAGCAACTCGGTCGCCGCATACACTATTCTCAGAATGACTT
GGTTGAGTACTCACCAGTCACAGAAAAGCATCTTACGGATGGCATGACA

GTAAGAGAATTATGCAGTGCTGCCATAACCATGAGTGATAACACTGCGG
CCAACTTACTTCTGACAACGATCGGAGGACCGAAGGAGCTAACCGCTTT
TTTGCACAACATGGGGGATCATGTAACTCGCCTTGATCGTTGGGAACCG
GAGCTGAATGAAGCCATACCAAACGACGAGCGTGACACCACGATGCCTG
TAGCAATGGCAACAACGTTGCGCAAACTATTAACTGGCGAACTACTTACT
CTAGCTTCCCGGCAACAATTAATAGACTGGATGGAGGCGGATAAAGTTG
CAGGACCACTTCTGCGCTCGGCCCTTCCGGCTGGCTGGTTTATTGCTGA
TAAATCTGGAGCCGGTGAGCGTGGGTCTCGCGGTATCATTGCAGCACTG
GGGCCAGATGGTAAGCCCTCCCGTATCGTAGTTATCTACACGACGGGGA
GTCAGGCAACTATGGATGAACGAAATAGACAGATCGCTGAGATAGGTGC
CTCACTGATTAAGCATTGGTAACTGTCAGACCAAGTTTACTCATATATACT
TTAGATTGATTTACCCCGGTTGATAATCAGAAAAGCCCCAAAAACAGGAA
GATTGTATAAGCAAATATTTAAATTGTAAACGTTAATATTTTGTTAAAATTC
GCGTTAAATTTTTGTTAAATCAGCTCATTTTTTAACCAATAGGCCGAAATC
GGCAAAATCCCTTATAAATCAAAAGAATAGCCCGAGATAGGGTTGAGTGT
TGTTCCAGTTTGGAACAAGAGTCCACTATTAAAGAACGTGGACTCCAACG
TCAAAGGGCGAAAAACCGTCTATCAGGGCGATGGCCCACTACGTGAACC
ATCACCCAAATCAAGTTTTTTGGGGTCGAGGTGCCGTAAAGCACTAAATC
GGAACCCTAAAGGGAGCCCCCGATTTAGAGCTTGACGGGGAAAGCCGG
CGAACGTGGCGAGAAAGGAAGGGAAGAAAGCGAAAGGAGCGGGCGCT
AGGGCGCTGGCAAGTGTAGCGGTCACGCTGCGCGTAACCACCACACCC
GCCGCGCTTAATGCGCCGCTACAGGGCGCGTAAAAGGATCTAGGTGAA
GATCCTTTTTGATAATCTCATGACCAAAATCCCTTAACGTGAGTTTTCGTT
CCACTGAGCGTCAGACCCCGTAGAAAAGATCAAAGGATCTTCTTGAGAT
CCTTTTTTTCTGCGCGTAATCTGCTGCTTGCAAACAAAAAAACCACCGCT
ACCAGCGGTGGTTTGTTTGCCGGATCAAGAGCTACCAACTCTTTTTCCGA
AGGTAACTGGCTTCAGCAGAGCGCAGATACCAAATACTGTCCTTCTAGT
GTAGCCGTAGTTAGGCCACCACTTCAAGAACTCTGTAGCACCGCCTACA
TACCTCGCTCTGCTAATCCTGTTACCAGTGGCTGCTGCCAGTGGCGATA
AGTCGTGTCTTACCGGGTTGGACTCAAGACGATAGTTACCGGATAAGGC
GCAGCGGTCGGGCTGAACGGGGGGTTCGTGCACACAGCCCAGCTTGGA
GCGAACGACCTACACCGAACTGAGATACCTACAGCGTGAGCTATGAGAA
AGCGCCACGCTTCCCGAAGGGAGAAAGGCGGACAGGTATCCGGTAAGC
GGCAGGGTCGGAACAGGAGAGCGCACGAGGGAGCTTCCAGGGGGAAA
CGCCTGGTATCTTTATAGTCCTGTCGGGTTTCGCCACCTCTGACTTGAGC
GTCGATTTTTGTGATGCTCGTCAGGGGGGCGGAGCCTATGGAAAAACGC
CAGCAACGCGGCCTTTTTACGGTTCCTGGCCTTTTGCTGGCCTTTTGCTC
ACATGTTCTTTCCTGCGTTATCCCCTGATTCTGTGGATAACCGTATTACC
GCCTTTGAGTGAGCTGATACCGCTCGCCGCAGCCGAACGACCGAGCGC
AGCGAGTCAGTGAGCGAGGAAGCTATGGTGCACTCTCAGTACAATCTGC
TCTGATGCCGCATAGTTAAGCCAGTATACACTCCGCTATCGCTACGTGAC
TGGGTCATGGCTGCGCCCCGACACCCGCCAACACCCGCTGACGCGCCC
TGACGGGCTTGTCTGCTCCCGGCATCCGCTTACAGACAAGCTGTGACCG
TCTCCGGGAGCTGCATGTGTCAGAGGTTTTCACCGTCATCACCGAAACG
CGCGAGGCAGCTGCGGTAAAGCTCATCAGCGTGGTCGTGCAGCGATTC
ACAGATGTCTGCCTGTTCATCCGCGTCCAGCTCGTTGAGTTTCTCCAGAA
GCGTTAATGTCTGGCTTCTGATAAAGCGGGCCATGTTAAGGGCGGTTTT
TTCCTGTTTGGTCACTGATGCCTCCGTGTAAGGGGGATTTCTGTTCATGG
GGGTAATGATACCGATGAAACGAGAGAGGATGCTCACGATACGGGTTAC
TGATGATGAACATGCCCGGTTACTGGAACGTTGTGAGGGTAAACAACTG

GCGGTATGGATGCGGCGGGACCAGAGAAAAATCACTCAGGGTCAATGC
CAGCCGAACGCCAGCAAGACGTAGCCCAGCGCGTCGGCCGCCATGCC
GGCGATAATGGCCTGCTTCTCGCCGAAACGTTTGGTGGCGGGACCAGT
GACGAAGGCTTGAGCGAGGGCGTGCAAGATTCCGAATACCGCAAGCGA
CAGGCCGATCATCGTCGCGCTCCAGCGAAAGCGGTCCTCGCCGAAAAT
GACCCAGAGCGCTGCCGGCACCTGTCCTACGAGTTGCATGATAAAGAAG
ACAGTCATAAGTGCGGCGACGATAGTCATGCCCCGCGCCCACCGGAAG
GAGCTGACTGGGTTGAAGGCTCTCAAGGGCATCGGTCGAGATCCCGGT
GCCTAATGAGTGAGCTAACTTACATTAATTGCGTTGCGCTCACTGCCCGC
TTTCCAGTCGGGAAACCTGTCGTGCCAGCTGCATTAATGAATCGGCCAA
CGCGCGGGGAGAGGCGGTTTGCGTATTGGGCGCCAGGGTGGTTTTTCT
TTTCACCAGTGAGACGGGCAACAGCTGATTGCCCTTCACCGCCTGGCCC
TGAGAGAGTTGCAGCAAGCGGTCCACGCTGGTTTGCCCCAGCAGGCGA
AAATCCTGTTTGATGGTGGTTAACGGCGGGATATAACATGAGCTGTCTTC
GGTATCGTCGTATCCCACTACCGAGATATCCGCACCAACGCGCAGCCCG
GACTCGGTAATGGCGCGCATTGCGCCCAGCGCCATCTGATCGTTGGCA
ACCAGCATCGCAGTGGGAACGATGCCCTCATTCAGCATTTGCATGGTTT
GTTGAAAACCGGACATGGCACTCCAGTCGCCTTCCCGTTCCGCTATCGG
CTGAATTTGATTGCGAGTGAGATATTTATGCCAGCCAGCCAGACGCAGA
CGCGCCGAGACAGAACTTAATGGGCCCGCTAACAGCGCGATTTGCTGG
TGACCCAATGCGACCAGATGCTCCACGCCCAGTCGCGTACCGTCTTCAT
GGGAGAAAATAATACTGTTGATGGGTGTCTGGTCAGAGACATCAAGAAA
TAACGCCGGAACATTAGTGCAGGCAGCTTCCACAGCAATGGCATCCTGG
TCATCCAGCGGATAGTTAATGATCAGCCCACTGACGCGTTGCGCGAGAA
GATTGTGCACCGCCGCTTTACAGGCTTCGACGCCGCTTCGTTCTACCAT
CGACACCACCACGCTGGCACCCAGTTGATCGGCGCGAGATTTAATCGCC
GCGACAATTTGCGACGGCGCGTGCAGGGCCAGACTGGAGGTGGCAACG
CCAATCAGCAACGACTGTTTGCCCGCCAGTTGTTGTGCCACGCGGTTGG
GAATGTAATTCAGCTCCGCCATCGCCGCTTCCACTTTTTCCCGCGTTTTC
GCAGAAACGTGGCTGGCCTGGTTCACCACGCGGGAAACGGTCTGATAA
GAGACACCGGCATACTCTGCGACATCGTATAACGTTACTGGTTTCACATT
CACCACCCTGAATTGACTCTCTTCCGGGCGCTATCATGCCATACCGCGA
AAGGTTTTGCGCCATTCGATGGTGTCCGGGATCTCGACGCTCTCCCTTA
TGCGACTCCTGCATTAGGAAGCAGCCCAGTAGTAGGTTGAGGCCGTTGA
GCACCGCCGCCGCAAGGAATGGTGCATGCCGGCATGCCGCCCTTTCGT
CTTCAAGAATTAATTCCCAATTCCCCAGGCATCAAATAAAACGAAAGGCT
CAGTCGAAAGACTGGGCCTTTCGTTTTATCTGTTGTTTGTCGGTGAACGC
TCTCCTGAGTAGGACAAATCCGCCGGGAGCGGATTTGAACGTTGCGAAG
CAACGGCCCGGAGGGTGGCGGGCAGGACGCCCGCCATAAACTGCCAG
GAATTAATTCCCCAGGCATCAAATAAAACGAAAGGCTCAGTCGAAAGACT
GGGCCTTTCGTTTTATCTGTTGTTTGTCGGTGAACGCTCTCCTGAGTAGG
ACAAATCCGCCGGGAGCGGATTTGAACGTTGCGAAGCAACGGCCCGGA
GGGTGGCGGGCAGGACGCCCGCCATAAACTGCCAGGAATTAATTCCCC
AGGCATCAAATAAAACGAAAGGCTCAGTCGAAAGACTGGGCCTTTCGTT
TTATCTGTTGTTTGTCGGTGAACGCTCTCCTGAGTAGGACAAATCCGCCG
GGAGCGGATTTGAACGTTGCGAAGCAACGGCCCGGAGGGTGGCGGGC
AGGACGCCCGCCATAAACTGCCAGGAATTAATTCCCCAGGCATCAAATA
AAACGAAAGGCTCAGTCGAAAGACTGGGCCTTTCGTTTTATCTGTTGTTT
GTCGGTGAACGCTCTCCTGAGTAGGACAAATCCGCCGGGAGCGGATTT
GAACGTTGCGAAGCAACGGCCCGGAGGGTGGCGGGCAGGACGCCCGC

CATAAACTGCCAGGAATTAATTCCCCAGGCATCAAATAAAACGAAAGGCT
CAGTCGAAAGACTGGGCCTTTCGTTTTATCTGTTGTTTGTCGGTGAACGC
TCTCCTGAGTAGGACAAATCCGCCGGGAGCGGATTTGAACGTTGCGAAG
CAACGGCCCGGAGGGTGGCGGGCAGGACGCCCGCCATAAACTGCCAG
GAATTGGGGATCGGAATTAATTCCCGGTTTAAACCGGGGATCTCGATCC
CGCGAAATTAATACGACTCACTATAGGGGAATTGTGAGCGGATAACAATT
CCCCTCTAGAAATAATTTTGTTTAACTTTAAGAAGGAGATATACATATGGC
TAGCTCGCGAATGATAACTTCTGCTCTTCATCGTGCGGCCGACTGGGCT
AAATCTGTGTTCTCTTCGGCGGCGCTGGGTGATCCTCGCCGTACTGCCC
GCTTGGTTAACGTCGCCGCCCAATTGGCAAAATATTCTGGTAAATCAATA
ACCATCTCATCAGAGGGTAGTAAGGCCGCACAGGAAGGCGCTTACCGAT
TTATCCGCAATCCCAACGTTTCTGCCGAGGCGATCAGAAAGGCTGGCGC
CATGCAAACAGTCAAGTTGGCTCAGGAGTTTCCCGAACTGCTGGCCATT
GAGGACACCACCTCTTTGAGTTATCGCCACCAGGTCGCCGAAGAGCTTG
GCAAGCTGGGCTCTATTCAGGATAAATCCCGCGGATGGTGGGTTCACTC
CGTTCTCTTGCTCGAGGCCACCACATTCCGCACCGTAGGATTACTGCAT
CAGGAGTGGTGGATGCGCCCGGATGACCCTGCCGATGCGGATGAAAAG
GAGAGTGGCAAATGGCTGGCAGCGGCCGCAACTAGCCGGTTACGCATG
GGCAGCATGATGAGCAACGTGATTGCGGTCTGTGACCGCGAAGCCGAT
ATTCATGCTTATCTGCAGGACAAACTGGCGCATAACGAGCGCTTCGTGG
TGCGCTCCAAGCACCCACGCAAGGACGTAGAGTCTGGGTTGTATCTGTA
CGACCATCTGAAGAACCAACCGGAGTTGGGTGGCTATCAGATCAGCATT
CCGCAAAAGGGCGTGGTGGATAAACGCGGTAAACGTAAAAATCGACCAG
CCCGCAAGGCGAGCTTGAGCCTGCGCAGTGGGCGCATCACGCTAAAAC
AGGGGAATATCACGCTCAACGCGGTGCTGGCCGAGGAGATTAACCCGC
CCAAGGGTGAGACCCCGTTGAAATGGTTGTTGCTGACCAGCGAACCGGT
CGAGTCGCTAGCCCAAGCCTTGCGCGTCATCGACATTTATACCCATCGC
TGGCGGATCGAGGAGTTCCATAAGGCATGGAAAACCGGAGCAGGAGCC
GAGAGGCAACGCATGGAGGAGCCGGATAATCTGGAGCGGATGGTCTCG
ATCCTCTCGTTTGTTGCGGTCAGGCTGTTACAGCTCAGAGAAAGCTTCAC
GCCGCCGCAAGCACTCAGGGCGCAAGGGCTGCTAAAGGAAGCGGAACA
CGTAGAAAGCCAGTCCGCAGAAACGGTGCTGACCCCGGATGAATGTCA
GCTACTGGGCTATCTGGACAAGGGAAAACGCAAGCGCAAAGAGAAAGC
AGGTAGCTTGCAGTGGGCTTACATGGCGATAGCTAGACTGGGCGGTTTT
ATGGACAGCAAGCGAACCGGAATTGCCAGCTGGGGCGCCCTCTGGGAA
GGTTGGGAAGCCCTGCAAAGTAAACTGGATGGCTTTCTTGCCGCCAAGG
ATCTGATGGCGCAGGGGATCAAGATCGGATCCTCTAGAGGAGGTGGCT
CAGAAGGTGGCGGATCTGAAGGTGGCTCTGGAACTAGTGAATTCATGAT
AACTTCTGCTCTTCATCGTGCGGCCGACTGGGCTAAATCTGTGTTCTCTT
CGGCGGCGCTGGGTGATCCTCGCCGTACTGCCCGCTTGGTTAACGTCG
CCGCCCAATTGGCAAAATATTCTGGTAAATCAATAACCATCTCATCAGAG
GGTAGTAAGGCCGCACAGGAAGGCGCTTACCGATTTATCCGCAATCCCA
ACGTTTCTGCCGAGGCGATCAGAAAGGCTGGCGCCATGCAAACAGTCAA
GTTGGCTCAGGAGTTTCCCGAACTGCTGGCCATTGAGGCCACCACCTCT
TTGAGTTATCGCCACCAGGTCGCCGAAGAGCTTGGCAAGCTGGGCTCTA
TTCAGGATAAATCCCGCGGATGGTGGGTTCACTCCGTTCTCTTGCTCGA
GGCCACCACATTCCGCACCGTAGGATTACTGCATCAGGAGTGGTGGATG
CGCCCGGATGACCCTGCCGATGCGGATGAAAAGGAGAGTGGCAAATGG
CTGGCAGCGGCCGCAACTAGCCGGTTACGCATGGGCAGCATGATGAGC
AACGTGATTGCGGTCTGTGACCGCGAAGCCGATATTCATGCTTATCTGC

AGGACAAACTGGCGCATAACGAGCGCTTCGTGGTGCGCTCCAAGCACC
CACGCAAGGACGTAGAGTCTGGGTTGTATCTGTACGACCATCTGAAGAA
CCAACCGGAGTTGGGTGGCTATCAGATCAGCATTCCGCAAAAGGGCGT
GGTGGATAAACGCGGTAAACGTAAAAATCGACCAGCCCGCAAGGCGAG
CTTGAGCCTGCGCAGTGGGCGCATCACGCTAAAACAGGGGAATATCAC
GCTCAACGCGGTGCTGGCCGAGGAGATTAACCCGCCCAAGGGTGAGAC
CCCGTTGAAATGGTTGTTGCTGACCAGCGAACCGGTCGAGTCGCTAGCC
CAAGCCTTGCGCGTCATCGACATTTATACCCATCGCTGGCGGATCGAGG
AGTTCCATAAGGCATGGAAAACCGGAGCAGGAGCCGAGAGGCAACGCA
TGGAGGAGCCGGATAATCTGGAGCGGATGGTCTCGATCCTCTCGTTTGT
TGCGGTCAGGCTGTTACAGCTCAGAGAAAGCTTCACGCCGCCGCAAGC
ACTCAGGGCGCAAGGGCTGCTAAAGGAAGCGGAACACGTAGAAAGCCA
GTCCGCAGAAACGGTGCTGACCCCGGATGAATGTCAGCTACTGGGCTAT
CTGGACAAGGGAAAACGCAAGCGCAAAGAGAAAGCAGGTAGCTTGCAG
TGGGCTTACATGGCGATAGCTAGACTGGGCGGTTTTATGGACAGCAAGC
GAACCGGAATTGCCAGCTGGGGCGCCCTCTGGGAAGGTTGGGAAGCCC
TGCAAAGTAAACTGGATGGCTTTCTTGCCGCCAAGGATCTGATGGCGCA
GGGGATCAAGATCCCC

pRC2109
CCGACACCATCGAATGGTGCAAAACCTTTCGCGGTATGGCATGATAGCG
CCCGGAAGAGAGTCAATTCAGGGTGGTGAATGTGAAACCAGTAACGTTA
TACGATGTCGCAGAGTATGCCGGTGTCTCTTATCAGACCGTTTCCCGCG
TGGTGAACCAGGCCAGCCACGTTTCTGCGAAAACGCGGGAAAAAGTGG
AAGCGGCGATGGCGGAGCTGAATTACATTCCCAACCGCGTGGCACAAC
AACTGGCGGGCAAACAGTCGTTGCTGATTGGCGTTGCCACCTCCAGTCT
GGCCCTGCACGCGCCGTCGCAAATTGTCGCGGCGATTAAATCTCGCGC
CGATCAACTGGGTGCCAGCGTGGTGGTGTCGATGGTAGAACGAAGCGG
CGTCGAAGCCTGTAAAGCGGCGGTGCACAATCTTCTCGCGCAACGCGT
CAGTGGGCTGATCATTAACTATCCGCTGGATGACCAGGATGCCATTGCT
GTGGAAGCTGCCTGCACTAATGTTCCGGCGTTATTTCTTGATGTCTCTGA
CCAGACACCCATCAACAGTATTATTTTCTCCCATGAAGACGGTACGCGAC
TGGGCGTGGAGCATCTGGTCGCATTGGGTCACCAGCAAATCGCGCTGTT
AGCGGGCCCATTAAGTTCTGTCTCGGCGCGTCTGCGTCTGGCTGGCTG
GCATAAATATCTCACTCGCAATCAAATTCAGCCGATAGCGGAACGGGAA
GGCGACTGGAGTGCCATGTCCGGTTTTCAACAAACCATGCAAATGCTGA
ATGAGGGCATCGTTCCCACTGCGATGCTGGTTGCCAACGATCAGATGGC
GCTGGGCGCAATGCGCGCCATTACCGAGTCCGGGCTGCGCGTTGGTGC
GGATATCTCGGTAGTGGGATACGACGATACCGAAGACAGCTCATGTTAT
ATCCCGCCGTTAACCACCATCAAACAGGATTTTCGCCTGCTGGGGCAAA
CCAGCGTGGACCGCTTGCTGCAACTCTCTCAGGGCCAGGCGGTGAAGG
GCAATCAGCTGTTGCCCGTCTCACTGGTGAAAAGAAAAACCACCCTGGC
GCCCAATACGCAAACCGCCTCTCCCCGCGCGTTGGCCGATTCATTAATG
CAGCTGGCACGACAGGTTTCCCGACTGGAAAGCGGGCAGTGAGCGCAA
CGCAATTAATGTGAGTTAGCTCACTCATTAGGCACAATTCTCATGTTTGA
CAGCTTATCATCGACTGCACGGTGCACCAATGCTTCTGGCGTCAGGCAG
CCATCGGAAGCTGTGGTATGGCTGTGCAGGTCGTAAATCACTGCATAAT
TCGTGTCGCTCAAGGCGCACTCCCGTTCTGGATAATGTTTTTTGCGCCG
ACATCATAACGGTTCTGGCAAATATTCTGAAATGAGCTGTTGACAATTAAT

CATCGGCTCGTATAATGTGTGGAATTGTGAGCGGATAACAATTTCACACA
GGAAACAGCCAGTCCGTTTAGGTGTTTTCACGAGCACTTCACCAACAAG
GACCATAGCATATGGCTAGCTCGCGAATGATAACTTCTGCTCTTCATCGT
GCGGCCGACTGGGCTAAATCTGTGTTCTCTTCGGCGGCGCTGGGTGAT
CCTCGCCGTACTGCCCGCTTGGTTAACGTCGCCGCCCAATTGGCAAAAT
ATTCTGGTAAATCAATAACCATCTCATCAGAGGGTAGTAAGGCCGCACAG
GAAGGCGCTTACCGATTTATCCGCAATCCCAACGTTTCTGCCGAGGCGA
TCAGAAAGGCTGGCGCCATGCAAACAGTCAAGTTGGCTCAGGAGTTTCC
CGAACTGCTGGCCATTGAGGACACCACCTCTTTGAGTTATCGCCACCAG
GTCGCCGAAGAGCTTGGCAAGCTGGGCTCTATTCAGGATAAATCCCGCG
GATGGTGGGTTCACTCCGTTCTCTTGCTCGAGGCCACCACATTCCGCAC
CGTAGGATTACTGCATCAGGAGTGGTGGATGCGCCCGGATGACCCTGC
CGATGCGGATGAAAAGGAGAGTGGCAAATGGCTGGCAGCGGCCGCAAC
TAGCCGGTTACGCATGGGCAGCATGATGAGCAACGTGATTGCGGTCTGT
GACCGCGAAGCCGATATTCATGCTTATCTGCAGGACAAACTGGCGCATA
ACGAGCGCTTCGTGGTGCGCTCCAAGCACCCACGCAAGGACGTAGAGT
CTGGGTTGTATCTGTACGACCATCTGAAGAACCAACCGGAGTTGGGTGG
CTATCAGATCAGCATTCCGCAAAAGGGCGTGGTGGATAAACGCGGTAAA
CGTAAAAATCGACCAGCCCGCAAGGCGAGCTTGAGCCTGCGCAGTGGG
CGCATCACGCTAAAACAGGGGAATATCACGCTCAACGCGGTGCTGGCC
GAGGAGATTAACCCGCCCAAGGGTGAGACCCCGTTGAAATGGTTGTTGC
TGACCAGCGAACCGGTCGAGTCGCTAGCCCAAGCCTTGCGCGTCATCG
ACATTTATACCCATCGCTGGCGGATCGAGGAGTTCCATAAGGCATGGAA
AACCGGAGCAGGAGCCGAGAGGCAACGCATGGAGGAGCCGGATAATCT
GGAGCGGATGGTCTCGATCCTCTCGTTTGTTGCGGTCAGGCTGTTACAG
CTCAGAGAAAGCTTCACGCCGCCGCAAGCACTCAGGGCGCAAGGGCTG
CTAAAGGAAGCGGAACACGTAGAAAGCCAGTCCGCAGAAACGGTGCTG
ACCCCGGATGAATGTCAGCTACTGGGCTATCTGGACAAGGGAAAACGCA
AGCGCAAAGAGAAAGCAGGTAGCTTGCAGTGGGCTTACATGGCGATAG
CTAGACTGGGCGGTTTTATGGACAGCAAGCGAACCGGAATTGCCAGCTG
GGGCGCCCTCTGGGAAGGTTGGGAAGCCCTGCAAAGTAAACTGGATGG
CTTTCTTGCCGCCAAGGATCTGATGGCGCAGGGGATCAAGATCGGATCC
TCTAGAGGAGGTGGCTCAGAAGGTGGCGGATCTGAAGGTGGCTCTGGA
ACTAGTGAATTCATGATAACTTCTGCTCTTCATCGTGCGGCCGACTGGGC
TAAATCTGTGTTCTCTTCGGCGGCGCTGGGTGATCCTCGCCGTACTGCC
CGCTTGGTTAACGTCGCCGCCCAATTGGCAAAATATTCTGGTAAATCAAT
AACCATCTCATCAGAGGGTAGTAAGGCCGCACAGGAAGGCGCTTACCGA
TTTATCCGCAATCCCAACGTTTCTGCCGAGGCGATCAGAAAGGCTGGCG
CCATGCAAACAGTCAAGTTGGCTCAGGAGTTTCCCGAACTGCTGGCCAT
TGAGGACACCACCTCTTTGAGTTATCGCCACCAGGTCGCCGAAGAGCTT
GGCAAGCTGGGCTCTATTCAGGATAAATCCCGCGGATGGTGGGTTCACT
CCGTTCTCTTGCTCGAGGCCACCACATTCCGCACCGTAGGATTACTGCA
TCAGGAGTGGTGGATGCGCCCGGATGACCCTGCCGATGCGGATGAAAA
GGAGAGTGGCAAATGGCTGGCAGCGGCCGCAACTAGCCGGTTACGCAT
GGGCAGCATGATGAGCAACGTGATTGCGGTCTGTGACCGCGAAGCCGA
TATTCATGCTTATCTGCAGGACAAACTGGCGCATAACGAGCGCTTCGTG
GTGCGCTCCAAGCACCCACGCAAGGACGTAGAGTCTGGGTTGTATCTGT
ACGACCATCTGAAGAACCAACCGGAGTTGGGTGGCTATCAGATCAGCAT
TCCGCAAAAGGGCGTGGTGGATAAACGCGGTAAACGTAAAAATCGACCA
GCCCGCAAGGCGAGCTTGAGCCTGCGCAGTGGGCGCATCACGCTAAAA

CAGGGGAATATCACGCTCAACGCGGTGCTGGCCGAGGAGATTAACCCG
CCCAAGGGTGAGACCCCGTTGAAATGGTTGTTGCTGACCAGCGAACCG
GTCGAGTCGCTAGCCCAAGCCTTGCGCGTCATCGACATTTATACCCATC
GCTGGCGGATCGAGGAGTTCCATAAGGCATGGAAAACCGGAGCAGGAG
CCGAGAGGCAACGCATGGAGGAGCCGGATAATCTGGAGCGGATGGTCT
CGATCCTCTCGTTTGTTGCGGTCAGGCTGTTACAGCTCAGAGAAAGCTT
CACGCCGCCGCAAGCACTCAGGGCGCAAGGGCTGCTAAAGGAAGCGGA
ACACGTAGAAAGCCAGTCCGCAGAAACGGTGCTGACCCCGGATGAATGT
CAGCTACTGGGCTATCTGGACAAGGGAAAACGCAAGCGCAAAGAGAAA
GCAGGTAGCTTGCAGTGGGCTTACATGGCGATAGCTAGACTGGGCGGT
TTTATGGACAGCAAGCGAACCGGAATTGCCAGCTGGGGCGCCCTCTGG
GAAGGTTGGGAAGCCCTGCAAAGTAAACTGGATGGCTTTCTTGCCGCCA
AGGATCTGATGGCGCAGGGGATCAAGATCCCCTGACCTGCAGGCAAGC
TTGGCACTGGCCGTCGTTTTACAACGTCGTGACTGGGAAAACCCTGGCG
TTACCCAACTTAATCGCCTTGCAGCACATCCCCCTTTCGCCAGCTGGCG
TAATAGCGAAGAGGCCCGCACCGATCGCCCTTCCCAACAGTTGCGCAG
CCTGAATGGCGAATGGCAGCTTGGCTGTTTTGGCGGATGAGATAAGATT
TTCAGCCTGATACAGATTAAATCAGAACGCAGAAGCGGTCTGATAAAACA
GAATTTGCCTGGCGGCAGTAGCGCGGTGGTCCCACCTGACCCCATGCC
GAACTCAGAAGTGAAACGCCGTAGCGCCGATGGTAGTGTGGGGTCTCC
CCATGCGAGAGTAGGGAACTGCCAGGCATCAAATAAAACGAAAGGCTCA
GTCGAAAGACTGGGCCTTTCGTTTTATCTGTTGTTTGTCGGTGAACGCTC
TCCTGAGTAGGACAAATCCGCCGGGAGCGGATTTGAACGTTGCGAAGCA
ACGGCCCGGAGGGTGGCGGGCAGGACGCCCGCCATAAACTGCCAGGC
ATCAAATTAAGCAGAAGGCCATCCTGACGGATGGCCTTTTTGCGTTTCTA
CAAACTCTTTTTGTTTATTTTTCTAAATACATTCAAATATGTATCCGCTCAT
GAGACAATAACCCTGATAAATGCTTCAATAATATTGAAAAAGGAAGAGTA
TGAGTATTCAACATTTCCGTGTCGCCCTTATTCCCTTTTTTGCGGCATTTT
GCCTTCCTGTTTTTGCTCACCCAGAAACGCTGGTGAAAGTAAAAGATGCT
GAAGATCAGTTGGGTGCACGAGTGGGTTACATCGAACTGGATCTCAACA
GCGGTAAGATCCTTGAGAGTTTTCGCCCCGAAGAACGTTCTCCAATGAT
GAGCACTTTTAAAGTTCTGCTATGTGGCGCGGTATTATCCCGTGTTGACG
CCGGGCAAGAGCAACTCGGTCGCCGCATACACTATTCTCAGAATGACTT
GGTTGAGTACTCACCAGTCACAGAAAAGCATCTTACGGATGGCATGACA
GTAAGAGAATTATGCAGTGCTGCCATAACCATGAGTGATAACACTGCGG
CCAACTTACTTCTGACAACGATCGGAGGACCGAAGGAGCTAACCGCTTT
TTTGCACAACATGGGGGATCATGTAACTCGCCTTGATCGTTGGGAACCG
GAGCTGAATGAAGCCATACCAAACGACGAGCGTGACACCACGATGCCTG
TAGCAATGGCAACAACGTTGCGCAAACTATTAACTGGCGAACTACTTACT
CTAGCTTCCCGGCAACAATTAATAGACTGGATGGAGGCGGATAAAGTTG
CAGGACCACTTCTGCGCTCGGCCCTTCCGGCTGGCTGGTTTATTGCTGA
TAAATCTGGAGCCGGTGAGCGTGGGTCTCGCGGTATCATTGCAGCACTG
GGGCCAGATGGTAAGCCCTCCCGTATCGTAGTTATCTACACGACGGGGA
GTCAGGCAACTATGGATGAACGAAATAGACAGATCGCTGAGATAGGTGC
CTCACTGATTAAGCATTGGTAACTGTCAGACCAAGTTTACTCATATATACT
TTAGATTGATTTACCCCGGTTGATAATCAGAAAAGCCCCAAAAACAGGAA
GATTGTATAAGCAAATATTTAAATTGTAAACGTTAATATTTTGTTAAAATTC
GCGTTAAATTTTTGTTAAATCAGCTCATTTTTTAACCAATAGGCCGAAATC
GGCAAAATCCCTTATAAATCAAAAGAATAGACCGAGATAGGGTTGAGTGT
TGTTCCAGTTTGGAACAAGAGTCCACTATTAAAGAACGTGGACTCCAACG

TCAAAGGGCGAAAAACCGTCTATCAGGGCGATGGCCCACTACGTGAACC
ATCACCCAAATCAAGTTTTTTGGGGTCGAGGTGCCGTAAAGCACTAAATC
GGAACCCTAAAGGGAGCCCCCGATTTAGAGCTTGACGGGGAAAGCCGG
CGAACGTGGCGAGAAAGGAAGGGAAGAAAGCGAAAGGAGCGGGCGCT
AGGGCGCTGGCAAGTGTAGCGGTCACGCTGCGCGTAACCACCACACCC
GCCGCGCTTAATGCGCCGCTACAGGGCGCGTAAAAGGATCTAGGTGAA
GATCCTTTTTGATAATCTCATGACCAAAATCCCTTAACGTGAGTTTTCGTT
CCACTGAGCGTCAGACCCCGTAGAAAAGATCAAAGGATCTTCTTGAGAT
CCTTTTTTTCTGCGCGTAATCTGCTGCTTGCAAACAAAAAAACCACCGCT
ACCAGCGGTGGTTTGTTTGCCGGATCAAGAGCTACCAACTCTTTTTCCGA
AGGTAACTGGCTTCAGCAGAGCGCAGATACCAAATACTGTCCTTCTAGT
GTAGCCGTAGTTAGGCCACCACTTCAAGAACTCTGTAGCACCGCCTACA
TACCTCGCTCTGCTAATCCTGTTACCAGTGGCTGCTGCCAGTGGCGATA
AGTCGTGTCTTACCGGGTTGGACTCAAGACGATAGTTACCGGATAAGGC
GCAGCGGTCGGGCTGAACGGGGGGTTCGTGCACACAGCCCAGCTTGGA
GCGAACGACCTACACCGAACTGAGATACCTACAGCGTGAGCTATGAGAA
AGCGCCACGCTTCCCGAAGGGAGAAAGGCGGACAGGTATCCGGTAAGC
GGCAGGGTCGGAACAGGAGAGCGCACGAGGGAGCTTCCAGGGGGAAA
CGCCTGGTATCTTTATAGTCCTGTCGGGTTTCGCCACCTCTGACTTGAGC
GTCGATTTTTGTGATGCTCGTCAGGGGGGCGGAGCCTATGGAAAAACGC
CAGCAACGCGGCCTTTTTACGGTTCCTGGCCTTTTGCTGGCCTTTTGCTC
ACATGTTCTTTCCTGCGTTATCCCCTGATTCTGTGGATAACCGTATTACC
GCCTTTGAGTGAGCTGATACCGCTCGCCGCAGCCGAACGACCGAGCGC
AGCGAGTCAGTGAGCGAGGAAGCGGAAGAGCGCCTGATGCGGTATTTT
CTCCTTACGCATCTGTGCGGTATTTCACACCGCATATATGGTGCACTCTC
AGTACAATCTGCTCTGATGCCGCATAGTTAAGCCAGTATACACTCCGCTA
TCGCTACGTGACTGGGTCATGGCTGCGCCCCGACACCCGCCAACACCC
GCTGACGCGCCCTGACGGGCTTGTCTGCTCCCGGCATCCGCTTACAGA
CAAGCTGTGACCGTCTCCGGGAGCTGCATGTGTCAGAGGTTTTCACCGT
CATCACCGAAACGCGCGAGGCAGCTGCGGTAAAGCTCATCAGCGTGGT
CGTGCAGCGATTCACAGATGTCTGCCTGTTCATCCGCGTCCAGCTCGTT
GAGTTTCTCCAGAAGCGTTAATGTCTGGCTTCTGATAAAGCGGGCCATG
TTAAGGGCGGTTTTTTCCTGTTTGGTCACTTGATGCCTCCGTGTAAGGGG
GAATTTCTGTTCATGGGGGTAATGATACCGATGAAACGAGAGAGGATGC
TCACGATACGGGTTACTGATGATGAACATGCCCGGTTACTGGAACGTTG
TGAGGGTAAACAACTGGCGGTATGGATGCGGCGGGACCAGAGAAAAAT
CACTCAGGGTCAATGCCAGCGCTTCGTTAATACAGATGTAGGTGTTCCA
CAGGGTAGCCAGCAGCATCCTGCGATGCAGATCCGGAACATAATGGTG
CAGGGCGCTGACTTCCGCGTTTCCAGACTTTACGAAACACGGAAACCGA
AGACCATTCATGTTGTTGCTCAGGTCGCAGACGTTTTGCAGCAGCAGTC
GCTTCACGTTCGCTCGCGTATCGGTGATTCATTCTGCTAACCAGTAAGG
CAACCCCGCCAGCCTAGCCGGGTCCTCAACGACAGGAGCACGATCATG
CGCACCCGTGGCCAGGACCCAACGCTGCCCGAAATT

pRC2113
CCGACACCATCGAATGGTGCAAAACCTTTCGCGGTATGGCATGATAGCG
CCCGGAAGAGAGTCAATTCAGGGTGGTGAATGTGAAACCAGTAACGTTA
TACGATGTCGCAGAGTATGCCGGTGTCTCTTATCAGACCGTTTCCCGCG
TGGTGAACCAGGCCAGCCACGTTTCTGCGAAAACGCGGGAAAAAGTGG
AAGCGGCGATGGCGGAGCTGAATTACATTCCCAACCGCGTGGCACAAC

AACTGGCGGGCAAACAGTCGTTGCTGATTGGCGTTGCCACCTCCAGTCT
GGCCCTGCACGCGCCGTCGCAAATTGTCGCGGCGATTAAATCTCGCGC
CGATCAACTGGGTGCCAGCGTGGTGGTGTCGATGGTAGAACGAAGCGG
CGTCGAAGCCTGTAAAGCGGCGGTGCACAATCTTCTCGCGCAACGCGT
CAGTGGGCTGATCATTAACTATCCGCTGGATGACCAGGATGCCATTGCT
GTGGAAGCTGCCTGCACTAATGTTCCGGCGTTATTTCTTGATGTCTCTGA
CCAGACACCCATCAACAGTATTATTTTCTCCCATGAAGACGGTACGCGAC
TGGGCGTGGAGCATCTGGTCGCATTGGGTCACCAGCAAATCGCGCTGTT
AGCGGGCCCATTAAGTTCTGTCTCGGCGCGTCTGCGTCTGGCTGGCTG
GCATAAATATCTCACTCGCAATCAAATTCAGCCGATAGCGGAACGGGAA
GGCGACTGGAGTGCCATGTCCGGTTTTCAACAAACCATGCAAATGCTGA
ATGAGGGCATCGTTCCCACTGCGATGCTGGTTGCCAACGATCAGATGGC
GCTGGGCGCAATGCGCGCCATTACCGAGTCCGGGCTGCGCGTTGGTGC
GGATATCTCGGTAGTGGGATACGACGATACCGAAGACAGCTCATGTTAT
ATCCCGCCGTTAACCACCATCAAACAGGATTTTCGCCTGCTGGGGCAAA
CCAGCGTGGACCGCTTGCTGCAACTCTCTCAGGGCCAGGCGGTGAAGG
GCAATCAGCTGTTGCCCGTCTCACTGGTGAAAAGAAAAACCACCCTGGC
GCCCAATACGCAAACCGCCTCTCCCCGCGCGTTGGCCGATTCATTAATG
CAGCTGGCACGACAGGTTTCCCGACTGGAAAGCGGGCAGTGAGCGCAA
CGCAATTAATGTGAGTTAGCTCACTCATTAGGCACAATTCTCATGTTTGA
CAGCTTATCATCGACTGCACGGTGCACCAATGCTTCTGGCGTCAGGCAG
CCATCGGAAGCTGTGGTATGGCTGTGCAGGTCGTAAATCACTGCATAAT
TCGTGTCGCTCAAGGCGCACTCCCGTTCTGGATAATGTTTTTTGCGCCG
ACATCATAACGGTTCTGGCAAATATTCTGAAATGAGCTGTTGACAATTAAT
CATCGGCTCGTATAATGTGTGGAATTGTGAGCGGATAACAATTTCACACA
GGAAACAGCCAGTCCGTTTAGGTGTTTTCACGAGCACTTCACCAACAAG
GACCATAGCAtaTGGCTAGCTCGCgaATGATAACTTCTGCTCTTCATCgtgc
ggccgactgggctaaatctgtgttctcttcggcggcgctgggtgatcctcgccgtactgcccgcttggttaacg
tcgccgcccaattggcaaaatattctggtaaatcaataaccatctcatcagagggtagtaaggccgcacag
gaaggcgcttaccgatttatccgcaatcccaacgtttctgccgaggcgatcagaaaggctggcgccatgca
aacagtcaagttggctcaggagtttcccgaactgctggccattgaggacaccacctctttgagttatcgccac
caggtcgccgaagagcttggcaagctgggctctattcaggataaatcccgcggatggtgggttcactccgtt
ctcttgctcgaggccaccacattccgcaccgtaggattactgcatcaggagtggtggatgcgcccggatga
ccctgccgatgcggatgaaaaggagagtggcaaatggctggcagcggccgcaactagccggttacgcat
gggcagcatgatgagcaacgtgattgcggtctgtgaccgcgaagccgatattcatgcttatctgcaggaca
aactggcgcataacgagcgcttcgtggtgcgctccaagcacccacgcaaggacgtagagtctgggttgtat
ctgtacgaccatctgaagaaccaaccggagttgggtggctatcagatcagcattccgcaaaagggcgtgg
tggataaacgcggtaaacgtaaaaatcgaccagcccgcaaggcgagcttgagcctgcgcagtgggcgc
atcacgctaaaacaggggaatatcacgctcaacgcggtgctggccgaggagattaacccgcccaagggt
gagaccccgttgaaatggttgttgctgaccagcgaaccggtcgagtcgctagcccaagccttgcgcgtcat
cgacatttatacccatcgctggcggatcgaggagttccataaggcatggaaaaccggagcaggagccga
gaggcaacgcatggaggagccggataatctggagcggatggtctcgatcctctcgtttgttgcggtcaggct
gttacagctcagagaaagcttcacgccgccgcaagcactcagggcgcaagggctgctaaaggaagcgg
aacacgtagaaagccagtccgcagaaacggtgctgaccccggatgaatgtcagctactgggctatctgga
caagggaaaacgcaagcgcaaagagaaagcaggtagcttgcagtgggcttacatggcgatagctaga
ctgggcggttttatggacagcaagcgaaccggaattgccagctggggcgccctctgggaaggttgggaag
ccctgcaaagtaaactggatggctttcttgccgccaaggatctgatggcgcaggggatcaagatcGgatcc
TCTAGAGGAGGTGGCTCAGAAGGTGGCGGATCTGAAGGTGGCTCTGGA
actagtgaattCatgataacttctgctcttcatcgtgcggccgactgggctaaatctgtgttctcttcggcggcg
ctgggtgatcctcgccgtactgcccgcttggttaacgtcgccgcccaattggcaaaatattctggtaaatcaat

aaccatctcatcagagggtagtaaggccgcacaggaaggcgcttaccgatttatccgcaatcccaacgttt
ctgccgaggcgatcagaaaggctggcgccatgcaaacagtcaagttggctcaggagtttcccgaactgct
ggccattgaggacaccacctctttgagttatcgccaccaggtcgccgaagagcttggcaagctgggctctat
tcaggataaatcccgcggatggtgggttcactccgttctcttgctcgaggccaccacattccgcaccgtagga
ttactgcatcaggagtggtggatgcgcccggatgaccctgccgatgcggatgaaaaggagagtggcaaat
ggctggcagcggccgcaactagccggttacgcatgggcagcatgatgagcaacgtgattgcggtctgtga
ccgcgaagccgatattcatgcttatctgcaggacaaactggcgcataacgagcgcttcgtggtgcgctcca
agcacccacgcaaggacgtagagtctgggttgtatctgtacgaccatctgaagaaccaaccggagttggg
tggctatcagatcagcattccgcaaaagggcgtggtggataaacgcggtaaacgtaaaaatcgaccagc
ccgcaaggcgagcttgagcctgcgcagtgggcgcatcacgctaaaacaggggaatatcacgctcaacg
cggtgctggccgaggagattaacccgcccaagggtgagaccccgttgaaatggttgttgctgaccagcga
accggtcgagtcgctagcccaagccttgcgcgtcatcgacatttatacccatcgctggcggatcgaggagtt
ccataaggcatggaaaaccggagcaggagccgagaggcaacgcatggaggagccggataatctgga
gcggatggtctcgatcctctcgtttgttgcggtcaggctgttacagctcagagaaagcttcacgccgccgcaa
gcactcagggcgcaagggctgctaaaggaagcggaacacgtagaaagccagtccgcagaaacggtg
ctgaccccggatgaatgtcagctactgggctatctggacaagggaaaacgcaagcgcaaagagaaagc
aggtagcttgcagtgggcttacatggcgatagctagactgggcggttttatggacagcaagcgaaccggaa
ttgccagctggggcgccctctgggaaggttgggaagccctgcaaagtaaactggatggctttcttgccgcca
aggatctgatggcgcaGGGGATCAAGATCCCCtgacctgcaGGCAAGCTTGGCACT
GGCCGTCGTTTTACAACGTCGTGACTGGGAAAACCCTGGCGTTACCCAA
CTTAATCGCCTTGCAGCACATCCCCCTTTCGCCAGCTGGCGTAATAGCG
AAGAGGCCCGCACCGATCGCCCTTCCCAACAGTTGCGCAGCCTGAATG
GCGAATGGCAGCTTGGCTGTTTTGGCGGATGAGATAAGATTTTCAGCCT
GATACAGATTAAATCAGAACGCAGAAGCGGTCTGATAAAACAGAATTTGC
CTGGCGGCAGTAGCGCGGTGGTCCCACCTGACCCCATGCCGAACTCAG
AAGTGAAACGCCGTAGCGCCGATGGTAGTGTGGGGTCTCCCCATGCGA
GAGTAGGGAACTGCCAGGCATCAAATAAAACGAAAGGCTCAGTCGAAAG
ACTGGGCCTTTCGTTTTATCTGTTGTTTGTCGGTGAACGCTCTCCTGAGT
AGGACAAATCCGCCGGGAGCGGATTTGAACGTTGCGAAGCAACGGCCC
GGAGGGTGGCGGGCAGGACGCCCGCCATAAACTGCCAGGCATCAAATT
AAGCAGAAGGCCATCCTGACGGATGGCCTTTTTGCGTTTCTACAAACTCT
TTTTGTTTATTTTTCTAAATACATTCAAATATGTATCCGCTCATGAGACAAT
AACCCTGATAAATGCTTCAATAATATTGAAAAAGGAAGAGTATGAGTATTC
AACATTTCCGTGTCGCCCTTATTCCCTTTTTTGCGGCATTTTGCCTTCCTG
TTTTTGCTCACCCAGAAACGCTGGTGAAAGTAAAAGATGCTGAAGATCAG
TTGGGTGCACGAGTGGGTTACATCGAACTGGATCTCAACAGCGGTAAGA
TCCTTGAGAGTTTTCGCCCCGAAGAACGTTCTCCAATGATGAGCACTTTT
AAAGTTCTGCTATGTGGCGCGGTATTATCCCGTGTTGACGCCGGGCAAG
AGCAACTCGGTCGCCGCATACACTATTCTCAGAATGACTTGGTTGAGTAC
TCACCAGTCACAGAAAAGCATCTTACGGATGGCATGACAGTAAGAGAATT
ATGCAGTGCTGCCATAACCATGAGTGATAACACTGCGGCCAACTTACTTC
TGACAACGATCGGAGGACCGAAGGAGCTAACCGCTTTTTTGCACAACAT
GGGGGATCATGTAACTCGCCTTGATCGTTGGGAACCGGAGCTGAATGAA
GCCATACCAAACGACGAGCGTGACACCACGATGCCTGTAGCAATGGCAA
CAACGTTGCGCAAACTATTAACTGGCGAACTACTTACTCTAGCTTCCCGG
CAACAATTAATAGACTGGATGGAGGCGGATAAAGTTGCAGGACCACTTC
TGCGCTCGGCCCTTCCGGCTGGCTGGTTTATTGCTGATAAATCTGGAGC
CGGTGAGCGTGGGTCTCGCGGTATCATTGCAGCACTGGGGCCAGATGG
TAAGCCCTCCCGTATCGTAGTTATCTACACGACGGGGAGTCAGGCAACT
ATGGATGAACGAAATAGACAGATCGCTGAGATAGGTGCCTCACTGATTA

AGCATTGGTAACTGTCAGACCAAGTTTACTCATATATACTTTAGATTGATT
TACCCCGGTTGATAATCAGAAAAGCCCCAAAAACAGGAAGATTGTATAAG
CAAATATTTATCAGATCTCTGATGATCAAACTTTTGGCGAAAATGAGACGT
TGATCGGCACGTAAGAGGTTCCAACTTTCACCATAATGAAATAAGATCAC
TACCGGGCGTATTTTTTGAGTTGTCGAGATTTTCAGGAGCTAAGGAAGCT
CAATGGAGAAAAAAATCACTGGATATACCACCGTTGATATATCCCAATGG
CATCGTAAAGAACATTTTGAGGCATTTCAGTCAGTTGCTCAATGTACCTA
TAACCAGACCGTTCAGCTGGATATTACGGCCTTTTTAAAGACCGTAAAGA
AAAATAAGCACAAGTTTTATCCGGCCTTTATTCACATTCTTGCCCGCCTG
ATGAATGCTCATCCGGAATTACGTATGGCAATGAAAGACGGTGAGCTGG
TGATATGGGATAGTGTTCACCCTTGTTACACCGTTTTCCATGAGCAAACT
GAAACGTTTTCATCGCTCTGGAGTGAATACCACGACGATTTCCGGCAGTT
TCTACACATATATTCGCAAGATGTGGCGTGTTACGGTGAAAACCTGGCCT
ATTTCCCTAAAGGGTTTATTGAGAATATGTTTTTCGTCTCAGCCAATCCCT
GGGTGAGTTTCACCAGTTTTGATTTAAACGTGGCCAATATGGACAACTTC
TTCGCCCCCGTTTTCACCATGGGCAAATATTATACGCAAGGCGACAAGG
TGCTGATGCCGCTGGCGATTCAGGTTCATCATGCCGTTTGTGATGGCTT
CCATGTCGGCAGAATGCTTAATGAATTACAACAGTACTGCGATGAGTGG
CAGGGCGGGGCGTAATTTTTTTAAGGCAGTTATTGGTGCCCTTAAACGC
CTGGTTGCTACGCCTGGTTCTTCTCAGGCCTGCTGGTAATCGCAGGCCT
TTTTATTTCTGAAGATCTCTGATGATCAATTTAAATTGTAAACGTTAATATT
TTGTTAAAATTCGCGTTAAATTTTTGTTAAATCAGCTCATTTTTTAACCAAT
AGGCCGAAATCGGCAAAATCCCTTATAAATCAAAAGAATAGACCGAGATA
GGGTTGAGTGTTGTTCCAGTTTGGAACAAGAGTCCACTATTAAAGAACGT
GGACTCCAACGTCAAAGGGCGAAAAACCGTCTATCAGGGCGATGGCCC
ACTACGTGAACCATCACCCAAATCAAGTTTTTTGGGGTCGAGGTGCCGT
AAAGCACTAAATCGGAACCCTAAAGGGAGCCCCCGATTTAGAGCTTGAC
GGGGAAAGCCGGCGAACGTGGCGAGAAAGGAAGGGAAGAAAGCGAAA
GGAGCGGGCGCTAGGGCGCTGGCAAGTGTAGCGGTCACGCTGCGCGT
AACCACCACACCCGCCGCGCTTAATGCGCCGCTACAGGGCGCGTAAAA
GGATCTAGGTGAAGATCCTTTTTGATAATCTCATGACCAAAATCCCTTAA
CGTGAGTTTTCGTTCCACTGAGCGTCAGACCCCGTAGAAAAGATCAAAG
GATCTTCTTGAGATCCTTTTTTTCTGCGCGTAATCTGCTGCTTGCAAACAA
AAAAACCACCGCTACCAGCGGTGGTTTGTTTGCCGGATCAAGAGCTACC
AACTCTTTTTCCGAAGGTAACTGGCTTCAGCAGAGCGCAGATACCAAATA
CTGTCCTTCTAGTGTAGCCGTAGTTAGGCCACCACTTCAAGAACTCTGTA
GCACCGCCTACATACCTCGCTCTGCTAATCCTGTTACCAGTGGCTGCTG
CCAGTGGCGATAAGTCGTGTCTTACCGGGTTGGACTCAAGACGATAGTT
ACCGGATAAGGCGCAGCGGTCGGGCTGAACGGGGGGTTCGTGCACACA
GCCCAGCTTGGAGCGAACGACCTACACCGAACTGAGATACCTACAGCGT
GAGCTATGAGAAAGCGCCACGCTTCCCGAAGGGAGAAAGGCGGACAGG
TATCCGGTAAGCGGCAGGGTCGGAACAGGAGAGCGCACGAGGGAGCTT
CCAGGGGGAAACGCCTGGTATCTTTATAGTCCTGTCGGGTTTCGCCACC
TCTGACTTGAGCGTCGATTTTTGTGATGCTCGTCAGGGGGGCGGAGCCT
ATGGAAAAACGCCAGCAACGCGGCCTTTTTACGGTTCCTGGCCTTTTGC
TGGCCTTTTGCTCACATGTTCTTTCCTGCGTTATCCCCTGATTCTGTGGA
TAACCGTATTACCGCCTTTGAGTGAGCTGATACCGCTCGCCGCAGCCGA
ACGACCGAGCGCAGCGAGTCAGTGAGCGAGGAAGCGGAAGAGCGCCT
GATGCGGTATTTTCTCCTTACGCATCTGTGCGGTATTTCACACCGCATAT
ATGGTGCACTCTCAGTACAATCTGCTCTGATGCCGCATAGTTAAGCCAGT

ATACACTCCGCTATCGCTACGTGACTGGGTCATGGCTGCGCCCCGACAC
CCGCCAACACCCGCTGACGCGCCCTGACGGGCTTGTCTGCTCCCGGCA
TCCGCTTACAGACAAGCTGTGACCGTCTCCGGGAGCTGCATGTGTCAGA
GGTTTTCACCGTCATCACCGAAACGCGCGAGGCAGCTGCGGTAAAGCTC
ATCAGCGTGGTCGTGCAGCGATTCACAGATGTCTGCCTGTTCATCCGCG
TCCAGCTCGTTGAGTTTCTCCAGAAGCGTTAATGTCTGGCTTCTGATAAA
GCGGGCCATGTTAAGGGCGGTTTTTTCCTGTTTGGTCACTTGATGCCTC
CGTGTAAGGGGGAATTTCTGTTCATGGGGGTAATGATACCGATGAAACG
AGAGAGGATGCTCACGATACGGGTTACTGATGATGAACATGCCCGGTTA
CTGGAACGTTGTGAGGGTAAACAACTGGCGGTATGGATGCGGCGGGAC
CAGAGAAAAATCACTCAGGGTCAATGCCAGCGCTTCGTTAATACAGATGT
AGGTGTTCCACAGGGTAGCCAGCAGCATCCTGCGATGCAGATCCGGAA
CATAATGGTGCAGGGCGCTGACTTCCGCGTTTCCAGACTTTACGAAACA
CGGAAACCGAAGACCATTCATGTTGTTGCTCAGGTCGCAGACGTTTTGC
AGCAGCAGTCGCTTCACGTTCGCTCGCGTATCGGTGATTCATTCTGCTA
ACCAGTAAGGCAACCCCGCCAGCCTAGCCGGGTCCTCAACGACAGGAG
CACGATCATGCGCACCCGTGGCCAGGACCCAACGCTGCCCGAAATT

pRC2114
CCGACACCATCGAATGGTGCAAAACCTTTCGCGGTATGGCATGATAGCG
CCCGGAAGAGAGTCAATTCAGGGTGGTGAATGTGAAACCAGTAACGTTA
TACGATGTCGCAGAGTATGCCGGTGTCTCTTATCAGACCGTTTCCCGCG
TGGTGAACCAGGCCAGCCACGTTTCTGCGAAAACGCGGGAAAAAGTGG
AAGCGGCGATGGCGGAGCTGAATTACATTCCCAACCGCGTGGCACAAC
AACTGGCGGGCAAACAGTCGTTGCTGATTGGCGTTGCCACCTCCAGTCT
GGCCCTGCACGCGCCGTCGCAAATTGTCGCGGCGATTAAATCTCGCGC
CGATCAACTGGGTGCCAGCGTGGTGGTGTCGATGGTAGAACGAAGCGG
CGTCGAAGCCTGTAAAGCGGCGGTGCACAATCTTCTCGCGCAACGCGT
CAGTGGGCTGATCATTAACTATCCGCTGGATGACCAGGATGCCATTGCT
GTGGAAGCTGCCTGCACTAATGTTCCGGCGTTATTTCTTGATGTCTCTGA
CCAGACACCCATCAACAGTATTATTTTCTCCCATGAAGACGGTACGCGAC
TGGGCGTGGAGCATCTGGTCGCATTGGGTCACCAGCAAATCGCGCTGTT
AGCGGGCCCATTAAGTTCTGTCTCGGCGCGTCTGCGTCTGGCTGGCTG
GCATAAATATCTCACTCGCAATCAAATTCAGCCGATAGCGGAACGGGAA
GGCGACTGGAGTGCCATGTCCGGTTTTCAACAAACCATGCAAATGCTGA
ATGAGGGCATCGTTCCCACTGCGATGCTGGTTGCCAACGATCAGATGGC
GCTGGGCGCAATGCGCGCCATTACCGAGTCCGGGCTGCGCGTTGGTGC
GGATATCTCGGTAGTGGGATACGACGATACCGAAGACAGCTCATGTTAT
ATCCCGCCGTTAACCACCATCAAACAGGATTTTCGCCTGCTGGGGCAAA
CCAGCGTGGACCGCTTGCTGCAACTCTCTCAGGGCCAGGCGGTGAAGG
GCAATCAGCTGTTGCCCGTCTCACTGGTGAAAAGAAAAACCACCCTGGC
GCCCAATACGCAAACCGCCTCTCCCCGCGCGTTGGCCGATTCATTAATG
CAGCTGGCACGACAGGTTTCCCGACTGGAAAGCGGGCAGTGAGCGCAA
CGCAATTAATGTGAGTTAGCTCACTCATTAGGCACAATTCTCATGTTTGA
CAGCTTATCATCGACTGCACGGTGCACCAATGCTTCTGGCGTCAGGCAG
CCATCGGAAGCTGTGGTATGGCTGTGCAGGTCGTAAATCACTGCATAAT
TCGTGTCGCTCAAGGCGCACTCCCGTTCTGGATAATGTTTTTTGCGCCG
ACATCATAACGGTTCTGGCAAATATTCTGAAATGAGCTGTTGACAATTAAT
CATCGGCTCGTATAATGTGTGGAATTGTGAGCGGATAACAATTTCACACA
GGAAACAGCCAGTCCGTTTAGGTGTTTTCACGAGCACTTCACCAACAAG

GACCATAGCAtaTGGCTAGCTCGCGAGTCGACGGCGGCCGCGaattCatgat
aacttctgctcttcatcgtgcggccgactgggctaaatctgtgttctcttcggcggcgctgggtgatcctcgccgt
actgcccgcttggttaacgtcgccgcccaattggcaaaatattctggtaaatcaataaccatctcatcagagg
gtagtaaggccgcacaggaaggcgcttaccgatttatccgcaatcccaacgtttctgccgaggcgatcaga
aaggctggcgccatgcaaacagtcaagttggctcaggagtttcccgaactgctggccattgaggacacca
cctctttgagttatcgccaccaggtcgccgaagagcttggcaagctgggctctattcaggataaatcccgcg
gatggtgggttcactccgttctcttgctcgaggccaccacattccgcaccgtaggattactgcatcaggagtg
gtggatgcgcccggatgaccctgccgatgcggatgaaaaggagagtggcaaatggctggcagcggccg
caactagccggttacgcatgggcagcatgatgagcaacgtgattgcggtctgtgaccgcgaagccgatatt
catgcttatctgcaggacaaactggcgcataacgagcgcttcgtggtgcgctccaagcacccacgcaagg
acgtagagtctgggttgtatctgtacgaccatctgaagaaccaaccggagttgggtggctatcagatcagca
ttccgcaaaagggcgtggtggataaacgcggtaaacgtaaaaatcgaccagcccgcaaggcgagcttg
agcctgcgcagtgggcgcatcacgctaaaacaggggaatatcacgctcaacgcggtgctggccgagga
gattaacccgcccaagggtgagaccccgttgaaatggttgttgctgaccagcgaaccggtcgagtcgctag
cccaagccttgcgcgtcatcgacatttatacccatcgctggcggatcgaggagttccataaggcatggaaa
accggagcaggagccgagaggcaacgcatggaggagccggataatctggagcggatggtctcgatcct
ctcgtttgttgcggtcaggctgttacagctcagagaaagcttcacgccgccgcaagcactcagggcgcaag
ggctgctaaaggaagcggaacacgtagaaagccagtccgcagaaacggtgctgaccccggatgaatgt
cagctactgggctatctggacaagggaaaacgcaagcgcaaagagaaagcaggtagcttgcagtgggc
ttacatggcgatagctagactgggcggttttatggacagcaagcgaaccggaattgccagctggggcgcc
ctctgggaaggttgggaagccctgcaaagtaaactggatggctttcttgccgccaaggatctgatggcgca
GGGGATCAAGATCCCCtgacctgcaGGCAAGCTTGGCACTGGCCGTCGTTT
TACAACGTCGTGACTGGGAAAACCCTGGCGTTACCCAACTTAATCGCCT
TGCAGCACATCCCCCTTTCGCCAGCTGGCGTAATAGCGAAGAGGCCCG
CACCGATCGCCCTTCCCAACAGTTGCGCAGCCTGAATGGCGAATGGCA
GCTTGGCTGTTTTGGCGGATGAGATAAGATTTTCAGCCTGATACAGATTA
AATCAGAACGCAGAAGCGGTCTGATAAAACAGAATTTGCCTGGCGGCAG
TAGCGCGGTGGTCCCACCTGACCCCATGCCGAACTCAGAAGTGAAACG
CCGTAGCGCCGATGGTAGTGTGGGGTCTCCCCATGCGAGAGTAGGGAA
CTGCCAGGCATCAAATAAAACGAAAGGCTCAGTCGAAAGACTGGGCCTT
TCGTTTTATCTGTTGTTTGTCGGTGAACGCTCTCCTGAGTAGGACAAATC
CGCCGGGAGCGGATTTGAACGTTGCGAAGCAACGGCCCGGAGGGTGG
CGGGCAGGACGCCCGCCATAAACTGCCAGGCATCAAATTAAGCAGAAG
GCCATCCTGACGGATGGCCTTTTTGCGTTTCTACAAACTCTTTTTGTTTAT
TTTTCTAAATACATTCAAATATGTATCCGCTCATGAGACAATAACCCTGAT
AAATGCTTCAATAATATTGAAAAAGGAAGAGTATGAGTATTCAACATTTCC
GTGTCGCCCTTATTCCCTTTTTTGCGGCATTTTGCCTTCCTGTTTTTGCTC
ACCCAGAAACGCTGGTGAAAGTAAAAGATGCTGAAGATCAGTTGGGTGC
ACGAGTGGGTTACATCGAACTGGATCTCAACAGCGGTAAGATCCTTGAG
AGTTTTCGCCCCGAAGAACGTTCTCCAATGATGAGCACTTTTAAAGTTCT
GCTATGTGGCGCGGTATTATCCCGTGTTGACGCCGGGCAAGAGCAACTC
GGTCGCCGCATACACTATTCTCAGAATGACTTGGTTGAGTACTCACCAGT
CACAGAAAAGCATCTTACGGATGGCATGACAGTAAGAGAATTATGCAGT
GCTGCCATAACCATGAGTGATAACACTGCGGCCAACTTACTTCTGACAAC
GATCGGAGGACCGAAGGAGCTAACCGCTTTTTTGCACAACATGGGGGAT
CATGTAACTCGCCTTGATCGTTGGGAACCGGAGCTGAATGAAGCCATAC
CAAACGACGAGCGTGACACCACGATGCCTGTAGCAATGGCAACAACGTT
GCGCAAACTATTAACTGGCGAACTACTTACTCTAGCTTCCCGGCAACAAT
TAATAGACTGGATGGAGGCGGATAAAGTTGCAGGACCACTTCTGCGCTC
GGCCCTTCCGGCTGGCTGGTTTATTGCTGATAAATCTGGAGCCGGTGAG

CGTGGGTCTCGCGGTATCATTGCAGCACTGGGGCCAGATGGTAAGCCC
TCCCGTATCGTAGTTATCTACACGACGGGGAGTCAGGCAACTATGGATG
AACGAAATAGACAGATCGCTGAGATAGGTGCCTCACTGATTAAGCATTG
GTAACTGTCAGACCAAGTTTACTCATATATACTTTAGATTGATTTACCCCG
GTTGATAATCAGAAAAGCCCCAAAAACAGGAAGATTGTATAAGCAAATAT
TTATCAGATCTCTGATGATCAAACTTTTGGCGAAAATGAGACGTTGATCG
GCACGTAAGAGGTTCCAACTTTCACCATAATGAAATAAGATCACTACCGG
GCGTATTTTTTGAGTTGTCGAGATTTTCAGGAGCTAAGGAAGCTCAATGG
AGAAAAAAATCACTGGATATACCACCGTTGATATATCCCAATGGCATCGT
AAAGAACATTTTGAGGCATTTCAGTCAGTTGCTCAATGTACCTATAACCA
GACCGTTCAGCTGGATATTACGGCCTTTTTAAAGACCGTAAAGAAAAATA
AGCACAAGTTTTATCCGGCCTTTATTCACATTCTTGCCCGCCTGATGAAT
GCTCATCCGGAATTACGTATGGCAATGAAAGACGGTGAGCTGGTGATAT
GGGATAGTGTTCACCCTTGTTACACCGTTTTCCATGAGCAAACTGAAACG
TTTTCATCGCTCTGGAGTGAATACCACGACGATTTCCGGCAGTTTCTACA
CATATATTCGCAAGATGTGGCGTGTTACGGTGAAAACCTGGCCTATTTCC
CTAAAGGGTTTATTGAGAATATGTTTTTCGTCTCAGCCAATCCCTGGGTG
AGTTTCACCAGTTTTGATTTAAACGTGGCCAATATGGACAACTTCTTCGC
CCCCGTTTTCACCATGGGCAAATATTATACGCAAGGCGACAAGGTGCTG
ATGCCGCTGGCGATTCAGGTTCATCATGCCGTTTGTGATGGCTTCCATG
TCGGCAGAATGCTTAATGAATTACAACAGTACTGCGATGAGTGGCAGGG
CGGGGCGTAATTTTTTTAAGGCAGTTATTGGTGCCCTTAAACGCCTGGTT
GCTACGCCTGGTTCTTCTCAGGCCTGCTGGTAATCGCAGGCCTTTTTATT
TCTGAAGATCTCTGATGATCAATTTAAATTGTAAACGTTAATATTTTGTTAA
AATTCGCGTTAAATTTTTGTTAAATCAGCTCATTTTTTAACCAATAGGCCG
AAATCGGCAAAATCCCTTATAAATCAAAAGAATAGACCGAGATAGGGTTG
AGTGTTGTTCCAGTTTGGAACAAGAGTCCACTATTAAAGAACGTGGACTC
CAACGTCAAAGGGCGAAAAACCGTCTATCAGGGCGATGGCCCACTACGT
GAACCATCACCCAAATCAAGTTTTTTGGGGTCGAGGTGCCGTAAAGCAC
TAAATCGGAACCCTAAAGGGAGCCCCCGATTTAGAGCTTGACGGGGAAA
GCCGGCGAACGTGGCGAGAAAGGAAGGGAAGAAAGCGAAAGGAGCGG
GCGCTAGGGCGCTGGCAAGTGTAGCGGTCACGCTGCGCGTAACCACCA
CACCCGCCGCGCTTAATGCGCCGCTACAGGGCGCGTAAAAGGATCTAG
GTGAAGATCCTTTTTGATAATCTCATGACCAAAATCCCTTAACGTGAGTTT
TCGTTCCACTGAGCGTCAGACCCCGTAGAAAAGATCAAAGGATCTTCTT
GAGATCCTTTTTTTCTGCGCGTAATCTGCTGCTTGCAAACAAAAAAACCA
CCGCTACCAGCGGTGGTTTGTTTGCCGGATCAAGAGCTACCAACTCTTT
TTCCGAAGGTAACTGGCTTCAGCAGAGCGCAGATACCAAATACTGTCCT
TCTAGTGTAGCCGTAGTTAGGCCACCACTTCAAGAACTCTGTAGCACCG
CCTACATACCTCGCTCTGCTAATCCTGTTACCAGTGGCTGCTGCCAGTG
GCGATAAGTCGTGTCTTACCGGGTTGGACTCAAGACGATAGTTACCGGA
TAAGGCGCAGCGGTCGGGCTGAACGGGGGGTTCGTGCACACAGCCCA
GCTTGGAGCGAACGACCTACACCGAACTGAGATACCTACAGCGTGAGCT
ATGAGAAAGCGCCACGCTTCCCGAAGGGAGAAAGGCGGACAGGTATCC
GGTAAGCGGCAGGGTCGGAACAGGAGAGCGCACGAGGGAGCTTCCAG
GGGGAAACGCCTGGTATCTTTATAGTCCTGTCGGGTTTCGCCACCTCTG
ACTTGAGCGTCGATTTTTGTGATGCTCGTCAGGGGGGCGGAGCCTATGG
AAAAACGCCAGCAACGCGGCCTTTTTACGGTTCCTGGCCTTTTGCTGGC
CTTTTGCTCACATGTTCTTTCCTGCGTTATCCCCTGATTCTGTGGATAACC
GTATTACCGCCTTTGAGTGAGCTGATACCGCTCGCCGCAGCCGAACGAC

CGAGCGCAGCGAGTCAGTGAGCGAGGAAGCGGAAGAGCGCCTGATGC
GGTATTTTCTCCTTACGCATCTGTGCGGTATTTCACACCGCATATATGGT
GCACTCTCAGTACAATCTGCTCTGATGCCGCATAGTTAAGCCAGTATACA
CTCCGCTATCGCTACGTGACTGGGTCATGGCTGCGCCCCGACACCCGC
CAACACCCGCTGACGCGCCCTGACGGGCTTGTCTGCTCCCGGCATCCG
CTTACAGACAAGCTGTGACCGTCTCCGGGAGCTGCATGTGTCAGAGGTT
TTCACCGTCATCACCGAAACGCGCGAGGCAGCTGCGGTAAAGCTCATCA
GCGTGGTCGTGCAGCGATTCACAGATGTCTGCCTGTTCATCCGCGTCCA
GCTCGTTGAGTTTCTCCAGAAGCGTTAATGTCTGGCTTCTGATAAAGCGG
GCCATGTTAAGGGCGGTTTTTTCCTGTTTGGTCACTTGATGCCTCCGTGT
AAGGGGGAATTTCTGTTCATGGGGGTAATGATACCGATGAAACGAGAGA
GGATGCTCACGATACGGGTTACTGATGATGAACATGCCCGGTTACTGGA
ACGTTGTGAGGGTAAACAACTGGCGGTATGGATGCGGCGGGACCAGAG
AAAAATCACTCAGGGTCAATGCCAGCGCTTCGTTAATACAGATGTAGGTG
TTCCACAGGGTAGCCAGCAGCATCCTGCGATGCAGATCCGGAACATAAT
GGTGCAGGGCGCTGACTTCCGCGTTTCCAGACTTTACGAAACACGGAAA
CCGAAGACCATTCATGTTGTTGCTCAGGTCGCAGACGTTTTGCAGCAGC
AGTCGCTTCACGTTCGCTCGCGTATCGGTGATTCATTCTGCTAACCAGTA
AGGCAACCCCGCCAGCCTAGCCGGGTCCTCAACGACAGGAGCACGATC
ATGCGCACCCGTGGCCAGGACCCAACGCTGCCCGAAATT

pRC2117
CCGACACCATCGAATGGTGCAAAACCTTTCGCGGTATGGCATGATAGCG
CCCGGAAGAGAGTCAATTCAGGGTGGTGAATGTGAAACCAGTAACGTTA
TACGATGTCGCAGAGTATGCCGGTGTCTCTTATCAGACCGTTTCCCGCG
TGGTGAACCAGGCCAGCCACGTTTCTGCGAAAACGCGGGAAAAAGTGG
AAGCGGCGATGGCGGAGCTGAATTACATTCCCAACCGCGTGGCACAAC
AACTGGCGGGCAAACAGTCGTTGCTGATTGGCGTTGCCACCTCCAGTCT
GGCCCTGCACGCGCCGTCGCAAATTGTCGCGGCGATTAAATCTCGCGC
CGATCAACTGGGTGCCAGCGTGGTGGTGTCGATGGTAGAACGAAGCGG
CGTCGAAGCCTGTAAAGCGGCGGTGCACAATCTTCTCGCGCAACGCGT
CAGTGGGCTGATCATTAACTATCCGCTGGATGACCAGGATGCCATTGCT
GTGGAAGCTGCCTGCACTAATGTTCCGGCGTTATTTCTTGATGTCTCTGA
CCAGACACCCATCAACAGTATTATTTTCTCCCATGAAGACGGTACGCGAC
TGGGCGTGGAGCATCTGGTCGCATTGGGTCACCAGCAAATCGCGCTGTT
AGCGGGCCCATTAAGTTCTGTCTCGGCGCGTCTGCGTCTGGCTGGCTG
GCATAAATATCTCACTCGCAATCAAATTCAGCCGATAGCGGAACGGGAA
GGCGACTGGAGTGCCATGTCCGGTTTTCAACAAACCATGCAAATGCTGA
ATGAGGGCATCGTTCCCACTGCGATGCTGGTTGCCAACGATCAGATGGC
GCTGGGCGCAATGCGCGCCATTACCGAGTCCGGGCTGCGCGTTGGTGC
GGATATCTCGGTAGTGGGATACGACGATACCGAAGACAGCTCATGTTAT
ATCCCGCCGTTAACCACCATCAAACAGGATTTTCGCCTGCTGGGGCAAA
CCAGCGTGGACCGCTTGCTGCAACTCTCTCAGGGCCAGGCGGTGAAGG
GCAATCAGCTGTTGCCCGTCTCACTGGTGAAAAGAAAAACCACCCTGGC
GCCCAATACGCAAACCGCCTCTCCCCGCGCGTTGGCCGATTCATTAATG
CAGCTGGCACGACAGGTTTCCCGACTGGAAAGCGGGCAGTGAGCGCAA
CGCAATTAATGTAAGTTAGCTCACTCATTAGGCACAATTCTCATGTTTGAC
AGCTTATCATCGACTGCACGGTGCACCAATGCTTCTGGCGTCAGGCAGC
CATCGGAAGCTGTGGTATGGCTGTGCAGGTCGTAAATCACTGCATAATT
CGTGTCGCTCAAGGCGCACTCCCGTTCTGGATAATGTTTTTTGCGCCGA

CATCATAACGGTTCTGGCAAATATTCTGAAATGAGCTGTTGACAATTAAT
CATCGGCTCGTATAATGTGTGGAATTGTGAGCGGATAACAATTTCACACA
GGAAACAGCCAGTCCGTTTAGGTGTTTTCACGAGCACTTCACCAACAAG
GACCATAGCATATGGCTAGCTCGCGAGTCGACGGCGGCCGCGAATTCA
TGATAACTTCTGCTCTTCATCGTGCGGCCGACTGGGCTAAATCTGTGTTC
TCTTCGGCGGCGCTGGGTGATCCTCGCCGTACTGCCCGCTTGGTTAAC
GTCGCCGCCCAATTGGCAAAATATTCTGGTAAATCAATAACCATCTCATC
AGAGGGTAGTAAGGCCGCACAGGAAGGCGCTTACCGATTTATCCGCAAT
CCCAACGTTTCTGCCGAGGCGATCAGAAAGGCTGGCGCCATGCAAACA
GTCAAGTTGGCTCAGGAGTTTCCCGAACTGCTGGCCATTGAGGACACCA
CCTCTTTGAGTTATCGCCACCAGGTCGCCGAAGAGCTTGGCAAGCTGGG
CTCTATTCAGGATAAATCCCGCGGATGGTGGGTTCACTCCGTTCTCTTGC
TCGAGGCCACCACATTCCGCACCGTAGGATTACTGCATCAGGAGTGGTG
GATGCGCCCGGATGACCCTGCCGATGCGGATGAAAAGGAGAGTGGCAA
ATGGCTGGCAGCGGCCGCAACTAGCCGGTTACGCATGGGCAGCATGAT
GAGCAACGTGATTGCGGTCTGTGACCGCGAAGCCGATATTCATGCTTAT
CTGCAGGACAAACTGGCGCATAACGAGCGCTTCGTGGTGCGCTCCAAG
CACCCACGCAAGGACGTAGAGTCTGGGTTGTATCTGTACGACCATCTGA
AGAACCAACCGGAGTTGGGTGGCTATCAGATCAGCATTCCGCAAAAGGG
CGTGGTGGATAAACGCGGTAAACGTAAAAATCGACCAGCCCGCAAGGC
GAGCTTGAGCCTGCGCAGTGGGCGCATCACGCTAAAACAGGGGAATAT
CACGCTCAACGCGGTGCTGGCCGAGGAGATTAACCCGCCCAAGGGTGA
GACCCCGTTGAAATGGTTGTTGCTGACCAGCGAACCGGTCGAGTCGCTA
GCCCAAGCCTTGCGCGTCATCGACATTTATACCCATCGCTGGCGGATCG
AGGAGTTCCATAAGGCATGGAAAACCGGAGCAGGAGCCGAGAGGCAAC
GCATGGAGGAGCCGGATAATCTGGAGCGGATGGTCTCGATCCTCTCGTT
TGTTGCGGTCAGGCTGTTACAGCTCAGAGAAAGCTTCACGCCGCCGCAA
GCACTCAGGGCGCAAGGGCTGCTAAAGGAAGCGGAACACGTAGAAAGC
CAGTCCGCAGAAACGGTGCTGACCCCGGATGAATGTCAGCTACTGGGC
TATCTGGACAAGGGAAAACGCAAGCGCAAAGAGAAAGCAGGTAGCTTGC
AGTGGGCTTACATGGCGATAGCTAGACTGGGCGGTTTTATGGACAGCAA
GCGAACCGGAATTGCCAGCTGGGGCGCCCTCTGGGAAGGTTGGGAAGC
CCTGCAAAGTAAACTGGATGGCTTTCTTGCCGCCAAGGATCTGATGGCG
CAGGGGATCAAGATCCCCTGACCTGCAGGCAAGCTTGGCACTGGCCGT
CGTTTTACAACGTCGTGACTGGGAAAACCCTGGCGTTACCCAACTTAATC
GCCTTGCAGCACATCCCCCTTTCGCCAGCTGGCGTAATAGCGAAGAGGC
CCGCACCGATCGCCCTTCCCAACAGTTGCGCAGCCTGAATGGCGAATG
GCAGCTTGGCTGTTTTGGCGGATGAGATAAGATTTTCAGCCTGATACAG
ATTAAATCAGAACGCAGAAGCGGTCTGATAAAACAGAATTTGCCTGGCG
GCAGTAGCGCGGTGGTCCCACCTGACCCCATGCCGAACTCAGAAGTGA
AACGCCGTAGCGCCGATGGTAGTGTGGGGTCTCCCCATGCGAGAGTAG
GGAACTGCCAGGCATCAAATAAAACGAAAGGCTCAGTCGAAAGACTGGG
CCTTTCGTTTTATCTGTTGTTTGTCGGTGAACGCTCTCCTGAGTAGGACA
AATCCGCCGGGAGCGGATTTGAACGTTGCGAAGCAACGGCCCGGAGGG
TGGCGGGCAGGACGCCCGCCATAAACTGCCAGGCATCAAATTAAGCAG
AAGGCCATCCTGACGGATGGCCTTTTTGCGTTTCTACAAACTCTTTTTGT
TTATTTTTCTAAATACATTCAAATATGTATCCGCTCATGAGACAATAACCC
TGATAAATGCTTCAATAATATTGAAAAAGGAAGAGTATGAGTATTCAACAT
TTCCGTGTCGCCCTTATTCCCTTTTTTGCGGCATTTTGCCTTCCTGTTTTT
GCTCACCCAGAAACGCTGGTGAAAGTAAAAGATGCTGAAGATCAGTTGG

GTGCACGAGTGGGTTACATCGAACTGGATCTCAACAGCGGTAAGATCCT
TGAGAGTTTTCGCCCCGAAGAACGTTCTCCAATGATGAGCACTTTTAAAG
TTCTGCTATGTGGCGCGGTATTATCCCGTGTTGACGCCGGGCAAGAGCA
ACTCGGTCGCCGCATACACTATTCTCAGAATGACTTGGTTGAGTACTCAC
CAGTCACAGAAAAGCATCTTACGGATGGCATGACAGTAAGAGAATTATG
CAGTGCTGCCATAACCATGAGTGATAACACTGCGGCCAACTTACTTCTGA
CAACGATCGGAGGACCGAAGGAGCTAACCGCTTTTTTGCACAACATGGG
GGATCATGTAACTCGCCTTGATCGTTGGGAACCGGAGCTGAATGAAGCC
ATACCAAACGACGAGCGTGACACCACGATGCCTGTAGCAATGGCAACAA
CGTTGCGCAAACTATTAACTGGCGAACTACTTACTCTAGCTTCCCGGCAA
CAATTAATAGACTGGATGGAGGCGGATAAAGTTGCAGGACCACTTCTGC
GCTCGGCCCTTCCGGCTGGCTGGTTTATTGCTGATAAATCTGGAGCCGG
TGAGCGTGGGTCTCGCGGTATCATTGCAGCACTGGGGCCAGATGGTAA
GCCCTCCCGTATCGTAGTTATCTACACGACGGGGAGTCAGGCAACTATG
GATGAACGAAATAGACAGATCGCTGAGATAGGTGCCTCACTGATTAAGC
ATTGGTAACTGTCAGACCAAGTTTACTCATATATACTTTAGATTGATTTAC
CCCGGTTGATAATCAGAAAAGCCCCAAAAACAGGAAGATTGTATAAGCAA
ATATTTAAATTGTAAACGTTAATATTTTGTTAAAATTCGCGTTAAATTTTTG
TTAAATCAGCTCATTTTTTAACCAATAGGCCGAAATCGGCAAAATCCCTTA
TAAATCAAAAGAATAGACCGAGATAGGGTTGAGTGTTGTTCCAGTTTGGA
ACAAGAGTCCACTATTAAAGAACGTGGACTCCAACGTCAAAGGGCGAAA
AACCGTCTATCAGGGCGATGGCCCACTACGTGAACCATCACCCAAATCA
AGTTTTTTGGGGTCGAGGTGCCGTAAAGCACTAAATCGGAACCCTAAAG
GGAGCCCCCGATTTAGAGCTTGACGGGGAAAGCCGGCGAACGTGGCGA
GAAAGGAAGGGAAGAAAGCGAAAGGAGCGGGCGCTAGGGCGCTGGCA
AGTGTAGCGGTCACGCTGCGCGTAACCACCACACCCGCCGCGCTTAAT
GCGCCGCTACAGGGCGCGTAAAAGGATCTAGGTGAAGATCCTTTTTGAT
AATCTCATGACCAAAATCCCTTAACGTGAGTTTTCGTTCCACTGAGCGTC
AGACCCCGTAGAAAAGATCAAAGGATCTTCTTGAGATCCTTTTTTTCTGC
GCGTAATCTGCTGCTTGCAAACAAAAAAACCACCGCTACCAGCGGTGGT
TTGTTTGCCGGATCAAGAGCTACCAACTCTTTTTCCGAAGGTAACTGGCT
TCAGCAGAGCGCAGATACCAAATACTGTCCTTCTAGTGTAGCCGTAGTTA
GGCCACCACTTCAAGAACTCTGTAGCACCGCCTACATACCTCGCTCTGC
TAATCCTGTTACCAGTGGCTGCTGCCAGTGGCGATAAGTCGTGTCTTAC
CGGGTTGGACTCAAGACGATAGTTACCGGATAAGGCGCAGCGGTCGGG
CTGAACGGGGGGTTCGTGCACACAGCCCAGCTTGGAGCGAACGACCTA
CACCGAACTGAGATACCTACAGCGTGAGCTATGAGAAAGCGCCACGCTT
CCCGAAGGGAGAAAGGCGGACAGGTATCCGGTAAGCGGCAGGGTCGG
AACAGGAGAGCGCACGAGGGAGCTTCCAGGGGGAAACGCCTGGTATCT
TTATAGTCCTGTCGGGTTTCGCCACCTCTGACTTGAGCGTCGATTTTTGT
GATGCTCGTCAGGGGGGCGGAGCCTATGGAAAAACGCCAGCAACGCGG
CCTTTTTACGGTTCCTGGCCTTTTGCTGGCCTTTTGCTCACATGTTCTTTC
CTGCGTTATCCCCTGATTCTGTGGATAACCGTATTACCGCCTTTGAGTGA
GCTGATACCGCTCGCCGCAGCCGAACGACCGAGCGCAGCGAGTCAGTG
AGCGAGGAAGCGGAAGAGCGCCTGATGCGGTATTTTCTCCTTACGCATC
TGTGCGGTATTTCACACCGCATATATGGTGCACTCTCAGTACAATCTGCT
CTGATGCCGCATAGTTAAGCCAGTATACACTCCGCTATCGCTACGTGACT
GGGTCATGGCTGCGCCCCGACACCCGCCAACACCCGCTGACGCGCCCT
GACGGGCTTGTCTGCTCCCGGCATCCGCTTACAGACAAGCTGTGACCGT
CTCCGGGAGCTGCATGTGTCAGAGGTTTTCACCGTCATCACCGAAACGC

GCGAGGCAGCTGCGGTAAAGCTCATCAGCGTGGTCGTGCAGCGATTCA
CAGATGTCTGCCTGTTCATCCGCGTCCAGCTCGTTGAGTTTCTCCAGAA
GCGTTAATGTCTGGCTTCTGATAAAGCGGGCCATGTTAAGGGCGGTTTT
TTCCTGTTTGGTCACTGATGCCTCCGTGTAAGGGGGATTTCTGTTCATGG
GGGTAATGATACCGATGAAACGAGAGAGGATGCTCACGATACGGGTTAC
TGATGATGAACATGCCCGGTTACTGGAACGTTGTGAGGGTAAACAACTG
GCGGTATGGATGCGGCGGGACCAGAGAAAAATCACTCAGGGTCAATGC
CAGCGCTTCGTTAATACAGATGTAGGTGTTCCACAGGGTAGCCAGCAGC
ATCCTGCGATGCAGATCCGGAACATAATGGTGCAGGGCGCTGACTTCCG
CGTTTCCAGACTTTACGAAACACGGAAACCGAAGACCATTCATGTTGTTG
CTCAGGTCGCAGACGTTTTGCAGCAGCAGTCGCTTCACGTTCGCTCGCG
TATCGGTGATTCATTCTGCTAACCAGTAAGGCAACCCCGCCAGCCTAGC
CGGGTCCTCAACGACAGGAGCACGATCATGCGCACCCGTGGCCAGGAC
CCAACGCTGCCCGAAATT

pRC2128
AAATCCGCCGGGAGCGGATTTGAACGTTGCGAAGCAACGGCCCGGAGG
GTGGCGGGCAGGACGCCCGCCATAAACTGCCAGGAATTAATTCCCCAG
GCATCAAATAAAACGAAAGGCTCAGTCGAAAGACTGGGCCTTTCGTTTTA
TCTGTTGTTTGTCGGTGAACGCTCTCCTGAGTAGGACAAATCCGCCGGG
AGCGGATTTGAACGTTGCGAAGCAACGGCCCGGAGGGTGGCGGGCAG
GACGCCCGCCATAAACTGCCAGGAATTAATTCCCCAGGCATCAAATAAA
ACGAAAGGCTCAGTCGAAAGACTGGGCCTTTCGTTTTATCTGTTGTTTGT
CGGTGAACGCTCTCCTGAGTAGGACAAATCCGCCGGGAGCGGATTTGA
ACGTTGCGAAGCAACGGCCCGGAGGGTGGCGGGCAGGACGCCCGCCA
TAAACTGCCAGGAATTGGGGATCGGAATTAATTCCCGGTTTAAACCGGG
GATCTCGATCCCGCGAAATTAATACGACTCACTATAGGGGAATTGTGAG
CGGATAACAATTCCCCTCTAGAAATAATTTTGTTTAACTTTAAGAAGGAGA
TATACATATGATAACTTCTGCTCTTCATCGTGCGGCCGACTGGGCTAAAT
CTGTGTTCTCTTCGGCGGCGCTGGGTGATCCTCGCCGTACTGCCCGCTT
GGTTAACGTCGCCGCCCAATTGGCAAAATATTCTGGTAAATCAATAACCA
TCTCATCAGAGGGTAGTAAGGCCGCACAGGAAGGCGCTTACCGATTTAT
CCGCAATCCCAACGTTTCTGCCGAGGCGATCAGAAAGGCTGGCGCCAT
GCAAACAGTCAAGTTGGCTCAGGAGTTTCCCGAACTGCTGGCCATTGAG
GACACCACCTCTTTGAGTTATCGCCACCAGGTCGCCGAAGAGCTTGGCA
AGCTGGGCTCTATTCAGGATAAATCCCGCGGATGGTGGGTTCACTCCGT
TCTCTTGCTCGAGGCCACCACATTCCGCACCGTAGGATTACTGCATCAG
GAGTGGTGGATGCGCCCGGATGACCCTGCCGATGCGGATGAAAAGGAG
AGTGGCAAATGGCTGGCAGCGGCCGCAACTAGCCGGTTACGCATGGGC
AGCATGATGAGCAACGTGATTGCGGTCTGTGACCGCGAAGCCGATATTC
ATGCTTATCTGCAGGACAAACTGGCGCATAACGAGCGCTTCGTGGTGCG
CTCCAAGCACCCACGCAAGGACGTAGAGTCTGGGTTGTATCTGTACGAC
CATCTGAAGAACCAACCGGAGTTGGGTGGCTATCAGATCAGCATTCCGC
AAAAGGGCGTGGTGGATAAACGCGGTAAACGTAAAAATCGACCAGCCCG
CAAGGCGAGCTTGAGCCTGCGCAGTGGGCGCATCACGCTAAAACAGGG
GAATATCACGCTCAACGCGGTGCTGGCCGAGGAGATTAACCCGCCCAA
GGGTGAGACCCCGTTGAAATGGTTGTTGCTGACCAGCGAACCGGTCGA
GTCGCTAGCCCAAGCCTTGCGCGTCATCGACATTTATACCCATCGCTGG
CGGATCGAGGAGTTCCATAAGGCATGGAAAACCGGAGCAGGAGCCGAG
AGGCAACGCATGGAGGAGCCGGATAATCTGGAGCGGATGGTCTCGATC

CTCTCGTTTGTTGCGGTCAGGCTGTTACAGCTCAGAGAAAGCTTCACGC
CGCCGCAAGCACTCAGGGCGCAAGGGCTGCTAAAGGAAGCGGAACACG
TAGAAAGCCAGTCCGCAGAAACGGTGCTGACCCCGGATGAATGTCAGCT
ACTGGGCTATCTGGACAAGGGAAAACGCAAGCGCAAAGAGAAAGCAGG
TAGCTTGCAGTGGGCTTACATGGCGATAGCTAGACTGGGCGGTTTTATG
GACAGCAAGCGAACCGGAATTGCCAGCTGGGGCGCCCTCTGGGAAGGT
TGGGAAGCCCTGCAAAGTAAACTGGATGGCTTTCTTGCCGCCAAGGATC
TGATGGCGCAGGGGATCAAGATCGGGGAATTCCTCGAGCCCGGGTGCT
TTGCCAAGGGTACCAATGTTTTAATGGCGGATGGGTCTATTGAATGTATT
GAAAACATTGAGGTTGGTAATAAGGTCATGGGTAAAGATGGCAGACCTC
GTGAGGTAATTAAATTGCCCAGAGGAAGAGAAACTATGTACAGCGTCGT
GCAGAAAAGTCAGCACAGAGCCCACAAAAGTGACTCAAGTCGTGAAGTG
CCAGAATTACTCAAGTTTACGTGTAATGCGACCCATGAGTTGGTTGTTAG
AACACCTCGTAGTGTCCGCCGTTTGTCTCGTACCATTAAGGGTGTCGAAT
ATTTTGAAGTTATTACTTTTGAGATGGGCCAAAAGAAAGCCCCCGACGGT
AGAATTGTTGAGCTTGTCAAGGAAGTTTCAAAGAGCTACCCAATATCTGA
GGGGCCTGAGAGAGCCAACGAATTAGTAGAATCCTATAGAAAGGCTTCA
AATAAAGCTTATTTTGAGTGGACTATTGAGGCCAGAGATCTTTCTCTGTT
GGGTTCCCATGTTCGTAAAGCTACCTACCAGACTTACGCTCCAATTCTTT
ATGAGAATGACCACTTTTTCGACTACATGCAAAAAAGTAAGTTTCATCTCA
CCATTGAAGGTCCAAAAGTACTTGCTTATTTACTTGGTTTATGGATTGGT
GATGGATTGTCTGACAGGGCAACTTTTTCGGTTGATTCCAGAGATACTTC
TTTGATGGAACGTGTTACTGAATATGCTGAAAAGTTGAATTTGTGCGCCG
AGTATAAGGACAGAAAAGAACCACAAGTTGCCAAAACTGTTAATTTGTAC
TCTAAAGTTGTCAGAGGTAATGGTATTCGCAATAATCTTAATACTGAGAAT
CCATTATGGGACGCTATTGTTGGCTTAGGATTCTTGAAGGACGGTGTCAA
AAATATTCCTTCTTTCTTGTCTACGGACAATATCGGTACTCGTGAAACATT
TCTTGCTGGTCTAATTGATTCTGATGGCTATGTTACTGATGAGCATGGTA
TTAAAGCAACAATAAAGACAATTCATACTTCTGTCAGAGATGGTTTGGTTT
CCCTTGCTCGTTCTTTAGGCTTAGTAGTCTCGGTTAACGCAGAACCTGCT
AAGGTTGACATGAATGTCACCAAACATAAAATTAGTTATGCTATTTATATG
TCTGGTGGAGATGTTTTGCTTAACGTTCTTTCGAAGTGTGCCGGCTCTAA
AAAATTCAGGCCTGCTCCCGCCGCTGCTTTTGCACGTGAGTGCCGCGGA
TTTTATTTCGAGTTACAAGAATTGAAGGAAGACGATTATTATGGGATTACT
TTATCTGATGATTCTGATCATCAGTTTTTGCTTGGATCCCAGGTTGTCGTC
CATGCATGCGGTGGCCTGACCGGTCTGAACTCAGGCCTCACGACAAATC
CTGGTGTATCCGCTTGGCAGGTCAACACAGCTTATACTGCGGGACAATT
GGTCACATATAACGGCAAGACGTATAAATGTTTGCAGCCCCACACCTCCT
TGGCAGGATGGGAACCATCCAACGTTCCTGCCTTGTGGCAGCTTCAATG
ACTGCAGGAAGGGGATCCGGCTGCTAACAAAGCCCGAAAGGAAGCTGA
GTTGGCTGCTGCCACCGCTGAGCAATAACTAGCATAACCCCTTGGGGCC
TCTAAACGGGTCTTGAGGGGTTTTTTGCTGAAAGGAGGAACTATATCCG
GATAACTACGTCAGGTGGCACTTTTCGGGGAAATGTGCGCGGAACCCCT
ATTTGTTTATTTTTCTAAATACATTCAAATATGTATCCGCTCATGAGACAAT
AACCCTGATAAATGCTTCAATAATATTGAAAAAGGAAGAGTATGAGTATTC
AACATTTCCGTGTCGCCCTTATTCCCTTTTTTGCGGCATTTTGCCTTCCTG
TTTTTGCTCACCCAGAAACGCTGGTGAAAGTAAAAGATGCTGAAGATCAG
TTGGGTGCACGAGTGGGTTACATCGAACTGGATCTCAACAGCGGTAAGA
TCCTTGAGAGTTTTCGCCCCGAAGAACGTTCTCCAATGATGAGCACTTTT
AAAGTTCTGCTATGTGGCGCGGTATTATCCCGTGTTGACGCCGGGCAAG

AGCAACTCGGTCGCCGCATACACTATTCTCAGAATGACTTGGTTGAGTAC
TCACCAGTCACAGAAAAGCATCTTACGGATGGCATGACAGTAAGAGAATT
ATGCAGTGCTGCCATAACCATGAGTGATAACACTGCGGCCAACTTACTTC
TGACAACGATCGGAGGACCGAAGGAGCTAACCGCTTTTTTGCACAACAT
GGGGGATCATGTAACTCGCCTTGATCGTTGGGAACCGGAGCTGAATGAA
GCCATACCAAACGACGAGCGTGACACCACGATGCCTGTAGCAATGGCAA
CAACGTTGCGCAAACTATTAACTGGCGAACTACTTACTCTAGCTTCCCGG
CAACAATTAATAGACTGGATGGAGGCGGATAAAGTTGCAGGACCACTTC
TGCGCTCGGCCCTTCCGGCTGGCTGGTTTATTGCTGATAAATCTGGAGC
CGGTGAGCGTGGGTCTCGCGGTATCATTGCAGCACTGGGGCCAGATGG
TAAGCCCTCCCGTATCGTAGTTATCTACACGACGGGGAGTCAGGCAACT
ATGGATGAACGAAATAGACAGATCGCTGAGATAGGTGCCTCACTGATTA
AGCATTGGTAACTGTCAGACCAAGTTTACTCATATATACTTTAGATTGATT
TACCCCGGTTGATAATCAGAAAAGCCCCAAAAACAGGAAGATTGTATAAG
CAAATATTTAAATTGTAAACGTTAATATTTTGTTAAAATTCGCGTTAAATTT
TTGTTAAATCAGCTCATTTTTTAACCAATAGGCCGAAATCGGCAAAATCC
CTTATAAATCAAAAGAATAGCCCGAGATAGGGTTGAGTGTTGTTCCAGTT
TGGAACAAGAGTCCACTATTAAAGAACGTGGACTCCAACGTCAAAGGGC
GAAAAACCGTCTATCAGGGCGATGGCCCACTACGTGAACCATCACCCAA
ATCAAGTTTTTTGGGGTCGAGGTGCCGTAAAGCACTAAATCGGAACCCT
AAAGGGAGCCCCCGATTTAGAGCTTGACGGGGAAAGCCGGCGAACGTG
GCGAGAAAGGAAGGGAAGAAAGCGAAAGGAGCGGGCGCTAGGGCGCT
GGCAAGTGTAGCGGTCACGCTGCGCGTAACCACCACACCCGCCGCGCT
TAATGCGCCGCTACAGGGCGCGTAAAAGGATCTAGGTGAAGATCCTTTT
TGATAATCTCATGACCAAAATCCCTTAACGTGAGTTTTCGTTCCACTGAG
CGTCAGACCCCGTAGAAAAGATCAAAGGATCTTCTTGAGATCCTTTTTTT
CTGCGCGTAATCTGCTGCTTGCAAACAAAAAAACCACCGCTACCAGCGG
TGGTTTGTTTGCCGGATCAAGAGCTACCAACTCTTTTTCCGAAGGTAACT
GGCTTCAGCAGAGCGCAGATACCAAATACTGTCCTTCTAGTGTAGCCGT
AGTTAGGCCACCACTTCAAGAACTCTGTAGCACCGCCTACATACCTCGC
TCTGCTAATCCTGTTACCAGTGGCTGCTGCCAGTGGCGATAAGTCGTGT
CTTACCGGGTTGGACTCAAGACGATAGTTACCGGATAAGGCGCAGCGGT
CGGGCTGAACGGGGGGTTCGTGCACACAGCCCAGCTTGGAGCGAACGA
CCTACACCGAACTGAGATACCTACAGCGTGAGCTATGAGAAAGCGCCAC
GCTTCCCGAAGGGAGAAAGGCGGACAGGTATCCGGTAAGCGGCAGGGT
CGGAACAGGAGAGCGCACGAGGGAGCTTCCAGGGGGAAACGCCTGGT
ATCTTTATAGTCCTGTCGGGTTTCGCCACCTCTGACTTGAGCGTCGATTT
TTGTGATGCTCGTCAGGGGGGCGGAGCCTATGGAAAAACGCCAGCAAC
GCGGCCTTTTTACGGTTCCTGGCCTTTTGCTGGCCTTTTGCTCACATGTT
CTTTCCTGCGTTATCCCCTGATTCTGTGGATAACCGTATTACCGCCTTTG
AGTGAGCTGATACCGCTCGCCGCAGCCGAACGACCGAGCGCAGCGAGT
CAGTGAGCGAGGAAGCTATGGTGCACTCTCAGTACAATCTGCTCTGATG
CCGCATAGTTAAGCCAGTATACACTCCGCTATCGCTACGTGACTGGGTC
ATGGCTGCGCCCCGACACCCGCCAACACCCGCTGACGCGCCCTGACGG
GCTTGTCTGCTCCCGGCATCCGCTTACAGACAAGCTGTGACCGTCTCCG
GGAGCTGCATGTGTCAGAGGTTTTCACCGTCATCACCGAAACGCGCGAG
GCAGCTGCGGTAAAGCTCATCAGCGTGGTCGTGCAGCGATTCACAGATG
TCTGCCTGTTCATCCGCGTCCAGCTCGTTGAGTTTCTCCAGAAGCGTTAA
TGTCTGGCTTCTGATAAAGCGGGCCATGTTAAGGGCGGTTTTTTCCTGTT
TGGTCACTGATGCCTCCGTGTAAGGGGGATTTCTGTTCATGGGGGTAAT

GATACCGATGAAACGAGAGAGGATGCTCACGATACGGGTTACTGATGAT
GAACATGCCCGGTTACTGGAACGTTGTGAGGGTAAACAACTGGCGGTAT
GGATGCGGCGGGACCAGAGAAAAATCACTCAGGGTCAATGCCAGCCGA
ACGCCAGCAAGACGTAGCCCAGCGCGTCGGCCGCCATGCCGGCGATAA
TGGCCTGCTTCTCGCCGAAACGTTTGGTGGCGGGACCAGTGACGAAGG
CTTGAGCGAGGGCGTGCAAGATTCCGAATACCGCAAGCGACAGGCCGA
TCATCGTCGCGCTCCAGCGAAAGCGGTCCTCGCCGAAAATGACCCAGA
GCGCTGCCGGCACCTGTCCTACGAGTTGCATGATAAAGAAGACAGTCAT
AAGTGCGGCGACGATAGTCATGCCCCGCGCCCACCGGAAGGAGCTGAC
TGGGTTGAAGGCTCTCAAGGGCATCGGTCGAGATCCCGGTGCCTAATGA
GTGAGCTAACTTACATTAATTGCGTTGCGCTCACTGCCCGCTTTCCAGTC
GGGAAACCTGTCGTGCCAGCTGCATTAATGAATCGGCCAACGCGCGGG
GAGAGGCGGTTTGCGTATTGGGCGCCAGGGTGGTTTTTCTTTTCACCAG
TGAGACGGGCAACAGCTGATTGCCCTTCACCGCCTGGCCCTGAGAGAG
TTGCAGCAAGCGGTCCACGCTGGTTTGCCCCAGCAGGCGAAAATCCTGT
TTGATGGTGGTTAACGGCGGGATATAACATGAGCTGTCTTCGGTATCGT
CGTATCCCACTACCGAGATATCCGCACCAACGCGCAGCCCGGACTCGG
TAATGGCGCGCATTGCGCCCAGCGCCATCTGATCGTTGGCAACCAGCAT
CGCAGTGGGAACGATGCCCTCATTCAGCATTTGCATGGTTTGTTGAAAA
CCGGACATGGCACTCCAGTCGCCTTCCCGTTCCGCTATCGGCTGAATTT
GATTGCGAGTGAGATATTTATGCCAGCCAGCCAGACGCAGACGCGCCG
AGACAGAACTTAATGGGCCCGCTAACAGCGCGATTTGCTGGTGACCCAA
TGCGACCAGATGCTCCACGCCCAGTCGCGTACCGTCTTCATGGGAGAAA
ATAATACTGTTGATGGGTGTCTGGTCAGAGACATCAAGAAATAACGCCG
GAACATTAGTGCAGGCAGCTTCCACAGCAATGGCATCCTGGTCATCCAG
CGGATAGTTAATGATCAGCCCACTGACGCGTTGCGCGAGAAGATTGTGC
ACCGCCGCTTTACAGGCTTCGACGCCGCTTCGTTCTACCATCGACACCA
CCACGCTGGCACCCAGTTGATCGGCGCGAGATTTAATCGCCGCGACAAT
TTGCGACGGCGCGTGCAGGGCCAGACTGGAGGTGGCAACGCCAATCAG
CAACGACTGTTTGCCCGCCAGTTGTTGTGCCACGCGGTTGGGAATGTAA
TTCAGCTCCGCCATCGCCGCTTCCACTTTTTCCCGCGTTTTCGCAGAAAC
GTGGCTGGCCTGGTTCACCACGCGGGAAACGGTCTGATAAGAGACACC
GGCATACTCTGCGACATCGTATAACGTTACTGGTTTCACATTCACCACCC
TGAATTGACTCTCTTCCGGGCGCTATCATGCCATACCGCGAAAGGTTTTG
CGCCATTCGATGGTGTCCGGGATCTCGACGCTCTCCCTTATGCGACTCC
TGCATTAGGAAGCAGCCCAGTAGTAGGTTGAGGCCGTTGAGCACCGCC
GCCGCAAGGAATGGTGCATGCCGGCATGCCGCCCTTTCGTCTTCAAGAA
TTAATTCCCAATTCCCCAGGCATCAAATAAAACGAAAGGCTCAGTCGAAA
GACTGGGCCTTTCGTTTTATCTGTTGTTTGTCGGTGAACGCTCTCCTGAG
TAGGACAAATCCGCCGGGAGCGGATTTGAACGTTGCGAAGCAACGGCC
CGGAGGGTGGCGGGCAGGACGCCCGCCATAAACTGCCAGGAATTAATT
CCCCAGGCATCAAATAAAACGAAAGGCTCAGTCGAAAGACTGGGCCTTT
CGTTTTATCTGTTGTTTGTCGGTGAACGCTCTCCTGAGTAGGACAAATCC
GCCGGGAGCGGATTTGAACGTTGCGAAGCAACGGCCCGGAGGGTGGC
GGGCAGGACGCCCGCCATAAACTGCCAGGAATTAATTCCCCAGGCATCA
AATAAAACGAAAGGCTCAGTCGAAAGACTGGGCCTTTCGTTTTATCTGTT
GTTTGTCGGTGAACGCTCTCCTGAGTAGGAC

pRC2143
GGGTGCTTTGCCAAGGGTACCAATGTTTTAATGGCGGATGGGTCTATTG
AATGTATTGAAAACATTGAGGTTGGTAATAAGGTCATGGGTAAAGATGGC
AGACCTCGTGAGGTAATTAAATTGCCCAGAGGAAGAGAAACTATGTACA
GCGTCGTGCAGAAAAGTCAGCACAGAGCCCACAAAAGTGACTCAAGTCG
TGAAGTGCCAGAATTACTCAAGTTTACGTGTAATGCGACCCATGAGTTGG
TTGTTAGAACACCTCGTAGTGTCCGCCGTTTGTCTCGTACCATTAAGGGT
GTCGAATATTTTGAAGTTATTACTTTTGAGATGGGCCAAAAGAAAGCCCC
CGACGGTAGAATTGTTGAGCTTGTCAAGGAAGTTTCAAAGAGCTACCCA
ATATCTGAGGGGCCTGAGAGAGCCAACGAATTAGTAGAATCCTATAGAA
AGGCTTCAAATAAAGCTTATTTTGAGTGGACTATTGAGGCCAGAGATCTT
TCTCTGTTGGGTTCCCATGTTCGTAAAGCTACCTACCAGACTTACGCTCC
AATTCTTTATGAGAATGACCACTTTTTCGACTACATGCAAAAAAGTAAGTT
TCATCTCACCATTGAAGGTCCAAAAGTACTTGCTTATTTACTTGGTTTATG
GATTGGTGATGGATTGTCTGACAGGGCAACTTTTTCGGTTGATTCCAGAG
ATACTTCTTTGATGGAACGTGTTACTGAATATGCTGAAAAGTTGAATTTGT
GCGCCGAGTATAAGGACAGAAAAGAACCACAAGTTGCCAAAACTGTTAA
TTTGTACTCTAAAGTTGTCAGAGGTAATGGTATTCGCAATAATCTTAATAC
TGAGAATCCATTATGGGACGCTATTGTTGGCTTAGGATTCTTGAAGGACG
GTGTCAAAAATATTCCTTCTTTCTTGTCTACGGACAATATCGGTACTCGTG
AAACATTTCTTGCTGGTCTAATTGATTCTGATGGCTATGTTACTGATGAGC
ATGGTATTAAAGCAACAATAAAGACAATTCATACTTCTGTCAGAGATGGTT
TGGTTTCCCTTGCTCGTTCTTTAGGCTTAGTAGTCTCGGTTAACGCAGAA
CCTGCTAAGGTTGACATGAATGTCACCAAACATAAAATTAGTTATGCTATT
TATATGTCTGGTGGAGATGTTTTGCTTAACGTTCTTTCGAAGTGTGCCGG
CTCTAAAAAATTCAGGCCTGCTCCCGCCGCTGCTTTTGCACGTGAGTGC
CGCGGATTTTATTTCGAGTTACAAGAATTGAAGGAAGACGATTATTATGG
GATTACTTTATCTGATGATTCTGATCATCAGTTTTTGCTTGGATCCCAGGT
TGTCGTCCATGCATGCGGTGGCCTGACCGGTCTGAACTCAGGCCTCAC
GACAAATCCTGGTGTATCCGCTTGGCAGGTCAACACAGCTTATACTGCG
GGACAATTGGTCACATATAACGGCAAGACGTATAAATGTTTGCAGCCCCA
CACCTCCTTGGCAGGATGGGAACCATCCAACGTTCCTGCCTTGTGGCAG
CTTCAATGACTGCAGGAAGGGGATCCGGCTGCTAACAAAGCCCGAAAG
GAAGCTGAGTTGGCTGCTGCCACCGCTGAGCAATAACTAGCATAACCCC
TTGGGGCCTCTAAACGGGTCTTGAGGGGTTTTTTGCTGAAAGGAGGAAC
TATATCCGGATAACTACGTCAGGTGGCACTTTTCGGGGAAATGTGCGCG
GAACCCCTATTTGTTTATTTTTCTAAATACATTCAAATATGTATCCGCTCAT
GAGACAATAACCCTGATAAATGCTTCAATAATATTGAAAAAGGAAGAGTA
TGAGTATTCAACATTTCCGTGTCGCCCTTATTCCCTTTTTTGCGGCATTTT
GCCTTCCTGTTTTTGCTCACCCAGAAACGCTGGTGAAAGTAAAAGATGCT
GAAGATCAGTTGGGTGCACGAGTGGGTTACATCGAACTGGATCTCAACA
GCGGTAAGATCCTTGAGAGTTTTCGCCCCGAAGAACGTTCTCCAATGAT
GAGCACTTTTAAAGTTCTGCTATGTGGCGCGGTATTATCCCGTGTTGACG
CCGGGCAAGAGCAACTCGGTCGCCGCATACACTATTCTCAGAATGACTT
GGTTGAGTACTCACCAGTCACAGAAAAGCATCTTACGGATGGCATGACA
GTAAGAGAATTATGCAGTGCTGCCATAACCATGAGTGATAACACTGCGG
CCAACTTACTTCTGACAACGATCGGAGGACCGAAGGAGCTAACCGCTTT
TTTGCACAACATGGGGGATCATGTAACTCGCCTTGATCGTTGGGAACCG
GAGCTGAATGAAGCCATACCAAACGACGAGCGTGACACCACGATGCCTG
TAGCAATGGCAACAACGTTGCGCAAACTATTAACTGGCGAACTACTTACT

CTAGCTTCCCGGCAACAATTAATAGACTGGATGGAGGCGGATAAAGTTG
CAGGACCACTTCTGCGCTCGGCCCTTCCGGCTGGCTGGTTTATTGCTGA
TAAATCTGGAGCCGGTGAGCGTGGGTCTCGCGGTATCATTGCAGCACTG
GGGCCAGATGGTAAGCCCTCCCGTATCGTAGTTATCTACACGACGGGGA
GTCAGGCAACTATGGATGAACGAAATAGACAGATCGCTGAGATAGGTGC
CTCACTGATTAAGCATTGGTAACTGTCAGACCAAGTTTACTCATATATACT
TTAGATTGATTTACCCCGGTTGATAATCAGAAAAGCCCCAAAAACAGGAA
GATTGTATAAGCAAATATTTAAATTGTAAACGTTAATATTTTGTTAAAATTC
GCGTTAAATTTTTGTTAAATCAGCTCATTTTTTAACCAATAGGCCGAAATC
GGCAAAATCCCTTATAAATCAAAAGAATAGCCCGAGATAGGGTTGAGTGT
TGTTCCAGTTTGGAACAAGAGTCCACTATTAAAGAACGTGGACTCCAACG
TCAAAGGGCGAAAAACCGTCTATCAGGGCGATGGCCCACTACGTGAACC
ATCACCCAAATCAAGTTTTTTGGGGTCGAGGTGCCGTAAAGCACTAAATC
GGAACCCTAAAGGGAGCCCCCGATTTAGAGCTTGACGGGGAAAGCCGG
CGAACGTGGCGAGAAAGGAAGGGAAGAAAGCGAAAGGAGCGGGCGCT
AGGGCGCTGGCAAGTGTAGCGGTCACGCTGCGCGTAACCACCACACCC
GCCGCGCTTAATGCGCCGCTACAGGGCGCGTAAAAGGATCTAGGTGAA
GATCCTTTTTGATAATCTCATGACCAAAATCCCTTAACGTGAGTTTTCGTT
CCACTGAGCGTCAGACCCCGTAGAAAAGATCAAAGGATCTTCTTGAGAT
CCTTTTTTTCTGCGCGTAATCTGCTGCTTGCAAACAAAAAAACCACCGCT
ACCAGCGGTGGTTTGTTTGCCGGATCAAGAGCTACCAACTCTTTTTCCGA
AGGTAACTGGCTTCAGCAGAGCGCAGATACCAAATACTGTCCTTCTAGT
GTAGCCGTAGTTAGGCCACCACTTCAAGAACTCTGTAGCACCGCCTACA
TACCTCGCTCTGCTAATCCTGTTACCAGTGGCTGCTGCCAGTGGCGATA
AGTCGTGTCTTACCGGGTTGGACTCAAGACGATAGTTACCGGATAAGGC
GCAGCGGTCGGGCTGAACGGGGGGTTCGTGCACACAGCCCAGCTTGGA
GCGAACGACCTACACCGAACTGAGATACCTACAGCGTGAGCTATGAGAA
AGCGCCACGCTTCCCGAAGGGAGAAAGGCGGACAGGTATCCGGTAAGC
GGCAGGGTCGGAACAGGAGAGCGCACGAGGGAGCTTCCAGGGGGAAA
CGCCTGGTATCTTTATAGTCCTGTCGGGTTTCGCCACCTCTGACTTGAGC
GTCGATTTTTGTGATGCTCGTCAGGGGGGCGGAGCCTATGGAAAAACGC
CAGCAACGCGGCCTTTTTACGGTTCCTGGCCTTTTGCTGGCCTTTTGCTC
ACATGTTCTTTCCTGCGTTATCCCCTGATTCTGTGGATAACCGTATTACC
GCCTTTGAGTGAGCTGATACCGCTCGCCGCAGCCGAACGACCGAGCGC
AGCGAGTCAGTGAGCGAGGAAGCTATGGTGCACTCTCAGTACAATCTGC
TCTGATGCCGCATAGTTAAGCCAGTATACACTCCGCTATCGCTACGTGAC
TGGGTCATGGCTGCGCCCCGACACCCGCCAACACCCGCTGACGCGCCC
TGACGGGCTTGTCTGCTCCCGGCATCCGCTTACAGACAAGCTGTGACCG
TCTCCGGGAGCTGCATGTGTCAGAGGTTTTCACCGTCATCACCGAAACG
CGCGAGGCAGCTGCGGTAAAGCTCATCAGCGTGGTCGTGCAGCGATTC
ACAGATGTCTGCCTGTTCATCCGCGTCCAGCTCGTTGAGTTTCTCCAGAA
GCGTTAATGTCTGGCTTCTGATAAAGCGGGCCATGTTAAGGGCGGTTTT
TTCCTGTTTGGTCACTGATGCCTCCGTGTAAGGGGGATTTCTGTTCATGG
GGGTAATGATACCGATGAAACGAGAGAGGATGCTCACGATACGGGTTAC
TGATGATGAACATGCCCGGTTACTGGAACGTTGTGAGGGTAAACAACTG
GCGGTATGGATGCGGCGGGACCAGAGAAAAATCACTCAGGGTCAATGC
CAGCCGAACGCCAGCAAGACGTAGCCCAGCGCGTCGGCCGCCATGCC
GGCGATAATGGCCTGCTTCTCGCCGAAACGTTTGGTGGCGGGACCAGT
GACGAAGGCTTGAGCGAGGGCGTGCAAGATTCCGAATACCGCAAGCGA
CAGGCCGATCATCGTCGCGCTCCAGCGAAAGCGGTCCTCGCCGAAAAT

GACCCAGAGCGCTGCCGGCACCTGTCCTACGAGTTGCATGATAAAGAAG
ACAGTCATAAGTGCGGCGACGATAGTCATGCCCCGCGCCCACCGGAAG
GAGCTGACTGGGTTGAAGGCTCTCAAGGGCATCGGTCGAGATCCCGGT
GCCTAATGAGTGAGCTAACTTACATTAATTGCGTTGCGCTCACTGCCCGC
TTTCCAGTCGGGAAACCTGTCGTGCCAGCTGCATTAATGAATCGGCCAA
CGCGCGGGGAGAGGCGGTTTGCGTATTGGGCGCCAGGGTGGTTTTTCT
TTTCACCAGTGAGACGGGCAACAGCTGATTGCCCTTCACCGCCTGGCCC
TGAGAGAGTTGCAGCAAGCGGTCCACGCTGGTTTGCCCCAGCAGGCGA
AAATCCTGTTTGATGGTGGTTAACGGCGGGATATAACATGAGCTGTCTTC
GGTATCGTCGTATCCCACTACCGAGATATCCGCACCAACGCGCAGCCCG
GACTCGGTAATGGCGCGCATTGCGCCCAGCGCCATCTGATCGTTGGCA
ACCAGCATCGCAGTGGGAACGATGCCCTCATTCAGCATTTGCATGGTTT
GTTGAAAACCGGACATGGCACTCCAGTCGCCTTCCCGTTCCGCTATCGG
CTGAATTTGATTGCGAGTGAGATATTTATGCCAGCCAGCCAGACGCAGA
CGCGCCGAGACAGAACTTAATGGGCCCGCTAACAGCGCGATTTGCTGG
TGACCCAATGCGACCAGATGCTCCACGCCCAGTCGCGTACCGTCTTCAT
GGGAGAAAATAATACTGTTGATGGGTGTCTGGTCAGAGACATCAAGAAA
TAACGCCGGAACATTAGTGCAGGCAGCTTCCACAGCAATGGCATCCTGG
TCATCCAGCGGATAGTTAATGATCAGCCCACTGACGCGTTGCGCGAGAA
GATTGTGCACCGCCGCTTTACAGGCTTCGACGCCGCTTCGTTCTACCAT
CGACACCACCACGCTGGCACCCAGTTGATCGGCGCGAGATTTAATCGCC
GCGACAATTTGCGACGGCGCGTGCAGGGCCAGACTGGAGGTGGCAACG
CCAATCAGCAACGACTGTTTGCCCGCCAGTTGTTGTGCCACGCGGTTGG
GAATGTAATTCAGCTCCGCCATCGCCGCTTCCACTTTTTCCCGCGTTTTC
GCAGAAACGTGGCTGGCCTGGTTCACCACGCGGGAAACGGTCTGATAA
GAGACACCGGCATACTCTGCGACATCGTATAACGTTACTGGTTTCACATT
CACCACCCTGAATTGACTCTCTTCCGGGCGCTATCATGCCATACCGCGA
AAGGTTTTGCGCCATTCGATGGTGTCCGGGATCTCGACGCTCTCCCTTA
TGCGACTCCTGCATTAGGAAGCAGCCCAGTAGTAGGTTGAGGCCGTTGA
GCACCGCCGCCGCAAGGAATGGTGCATGCCGGCATGCCGCCCTTTCGT
CTTCAAGAATTAATTCCCAATTCCCCAGGCATCAAATAAAACGAAAGGCT
CAGTCGAAAGACTGGGCCTTTCGTTTTATCTGTTGTTTGTCGGTGAACGC
TCTCCTGAGTAGGACAAATCCGCCGGGAGCGGATTTGAACGTTGCGAAG
CAACGGCCCGGAGGGTGGCGGGCAGGACGCCCGCCATAAACTGCCAG
GAATTAATTCCCCAGGCATCAAATAAAACGAAAGGCTCAGTCGAAAGACT
GGGCCTTTCGTTTTATCTGTTGTTTGTCGGTGAACGCTCTCCTGAGTAGG
ACAAATCCGCCGGGAGCGGATTTGAACGTTGCGAAGCAACGGCCCGGA
GGGTGGCGGGCAGGACGCCCGCCATAAACTGCCAGGAATTAATTCCCC
AGGCATCAAATAAAACGAAAGGCTCAGTCGAAAGACTGGGCCTTTCGTT
TTATCTGTTGTTTGTCGGTGAACGCTCTCCTGAGTAGGACAAATCCGCCG
GGAGCGGATTTGAACGTTGCGAAGCAACGGCCCGGAGGGTGGCGGGC
AGGACGCCCGCCATAAACTGCCAGGAATTAATTCCCCAGGCATCAAATA
AAACGAAAGGCTCAGTCGAAAGACTGGGCCTTTCGTTTTATCTGTTGTTT
GTCGGTGAACGCTCTCCTGAGTAGGACAAATCCGCCGGGAGCGGATTT
GAACGTTGCGAAGCAACGGCCCGGAGGGTGGCGGGCAGGACGCCCGC
CATAAACTGCCAGGAATTAATTCCCCAGGCATCAAATAAAACGAAAGGCT
CAGTCGAAAGACTGGGCCTTTCGTTTTATCTGTTGTTTGTCGGTGAACGC
TCTCCTGAGTAGGACAAATCCGCCGGGAGCGGATTTGAACGTTGCGAAG
CAACGGCCCGGAGGGTGGCGGGCAGGACGCCCGCCATAAACTGCCAG
GAATTGGGGATCGGAATTAATTCCCGGTTTAAACCGGGGATCTCGATCC

CGCGAAATTAATACGACTCACTATAGGGGAATTGTGAGCGGATAACAATT
CCCCTCTAGAAATAATTTTGTTTAACTTTAAGAAGGAGATATACATATGGC
TAGCTCGCGAATGATAACTTCTGCTCTTCATCGTGCGGCCGACTGGGCT
AAATCTGTGTTCTCTTCGGCGGCGCTGGGTGATCCTCGCCGTACTGCCC
GCTTGGTTAACGTCGCCGCCCAATTGGCAAAATATTCTGGTAAATCAATA
ACCATCTCATCAGAGGGTAGTAAGGCCGCACAGGAAGGCGCTTACCGAT
TTATCCGCAATCCCAACGTTTCTGCCGAGGCGATCAGAAAGGCTGGCGC
CATGCAAACAGTCAAGTTGGCTCAGGAGTTTCCCGAACTGCTGGCCATT
GAGGCCACCACCTCTTTGAGTTATCGCCACCAGGTCGCCGAAGAGCTTG
GCAAGCTGGGCTCTATTCAGGATAAATCCCGCGGATGGTGGGTTCACTC
CGTTCTCTTGCTCGAGGCCACCACATTCCGCACCGTAGGATTACTGCAT
CAGGAGTGGTGGATGCGCCCGGATGACCCTGCCGATGCGGATGAAAAG
GAGAGTGGCAAATGGCTGGCAGCGGCCGCAACTAGCCGGTTACGCATG
GGCAGCATGATGAGCAACGTGATTGCGGTCTGTGACCGCGAAGCCGAT
ATTCATGCTTATCTGCAGGACAAACTGGCGCATAACGAGCGCTTCGTGG
TGCGCTCCAAGCACCCACGCAAGGACGTAGAGTCTGGGTTGTATCTGTA
CGACCATCTGAAGAACCAACCGGAGTTGGGTGGCTATCAGATCAGCATT
CCGCAAAAGGGCGTGGTGGATAAACGCGGTAAACGTAAAAATCGACCAG
CCCGCAAGGCGAGCTTGAGCCTGCGCAGTGGGCGCATCACGCTAAAAC
AGGGGAATATCACGCTCAACGCGGTGCTGGCCGAGGAGATTAACCCGC
CCAAGGGTGAGACCCCGTTGAAATGGTTGTTGCTGACCAGCGAACCGGT
CGAGTCGCTAGCCCAAGCCTTGCGCGTCATCGACATTTATACCCATCGC
TGGCGGATCGAGGAGTTCCATAAGGCATGGAAAACCGGAGCAGGAGCC
GAGAGGCAACGCATGGAGGAGCCGGATAATCTGGAGCGGATGGTCTCG
ATCCTCTCGTTTGTTGCGGTCAGGCTGTTACAGCTCAGAGAAAGCTTCAC
GCCGCCGCAAGCACTCAGGGCGCAAGGGCTGCTAAAGGAAGCGGAACA
CGTAGAAAGCCAGTCCGCAGAAACGGTGCTGACCCCGGATGAATGTCA
GCTACTGGGCTATCTGGACAAGGGAAAACGCAAGCGCAAAGAGAAAGC
AGGTAGCTTGCAGTGGGCTTACATGGCGATAGCTAGACTGGGCGGTTTT
ATGGACAGCAAGCGAACCGGAATTGCCAGCTGGGGCGCCCTCTGGGAA
GGTTGGGAAGCCCTGCAAAGTAAACTGGATGGCTTTCTTGCCGCCAAGG
ATCTGATGGCGCAGGGGATCAAGATCGGATCCTCTAGAGGAGGTGGCT
CAGAAGGTGGCGGATCTGAAGGTGGCTCTGGAACTAGTGAATTCATGAT
AACTTCTGCTCTTCATCGTGCGGCCGACTGGGCTAAATCTGTGTTCTCTT
CGGCGGCGCTGGGTGATCCTCGCCGTACTGCCCGCTTGGTTAACGTCG
CCGCCCAATTGGCAAAATATTCTGGTAAATCAATAACCATCTCATCAGAG
GGTAGTAAGGCCGCACAGGAAGGCGCTTACCGATTTATCCGCAATCCCA
ACGTTTCTGCCGAGGCGATCAGAAAGGCTGGCGCCATGCAAACAGTCAA
GTTGGCTCAGGAGTTTCCCGAACTGCTGGCCATTGAGGACACCACCTCT
TTGAGTTATCGCCACCAGGTCGCCGAAGAGCTTGGCAAGCTGGGCTCTA
TTCAGGATAAATCCCGCGGATGGTGGGTTCACTCCGTTCTCTTGCTCGA
GGCCACCACATTCCGCACCGTAGGATTACTGCATCAGGAGTGGTGGATG
CGCCCGGATGACCCTGCCGATGCGGATGAAAAGGAGAGTGGCAAATGG
CTGGCAGCGGCCGCAACTAGCCGGTTACGCATGGGCAGCATGATGAGC
AACGTGATTGCGGTCTGTGACCGCGAAGCCGATATTCATGCTTATCTGC
AGGACAAACTGGCGCATAACGAGCGCTTCGTGGTGCGCTCCAAGCACC
CACGCAAGGACGTAGAGTCTGGGTTGTATCTGTACGACCATCTGAAGAA
CCAACCGGAGTTGGGTGGCTATCAGATCAGCATTCCGCAAAAGGGCGT
GGTGGATAAACGCGGTAAACGTAAAAATCGACCAGCCCGCAAGGCGAG
CTTGAGCCTGCGCAGTGGGCGCATCACGCTAAAACAGGGGAATATCAC

GCTCAACGCGGTGCTGGCCGAGGAGATTAACCCGCCCAAGGGTGAGAC
CCCGTTGAAATGGTTGTTGCTGACCAGCGAACCGGTCGAGTCGCTAGCC
CAAGCCTTGCGCGTCATCGACATTTATACCCATCGCTGGCGGATCGAGG
AGTTCCATAAGGCATGGAAAACCGGAGCAGGAGCCGAGAGGCAACGCA
TGGAGGAGCCGGATAATCTGGAGCGGATGGTCTCGATCCTCTCGTTTGT
TGCGGTCAGGCTGTTACAGCTCAGAGAAAGCTTCACGCCGCCGCAAGC
ACTCAGGGCGCAAGGGCTGCTAAAGGAAGCGGAACACGTAGAAAGCCA
GTCCGCAGAAACGGTGCTGACCCCGGATGAATGTCAGCTACTGGGCTAT
CTGGACAAGGGAAAACGCAAGCGCAAAGAGAAAGCAGGTAGCTTGCAG
TGGGCTTACATGGCGATAGCTAGACTGGGCGGTTTTATGGACAGCAAGC
GAACCGGAATTGCCAGCTGGGGCGCCCTCTGGGAAGGTTGGGAAGCCC
TGCAAAGTAAACTGGATGGCTTTCTTGCCGCCAAGGATCTGATGGCGCA
GGGGATCAAGATCCCC

pRC2147
CTGTGCCTTCTAGTTGCCAGCCATCTGTTGTTTGCCCCTCCCCCGTGCC
TTCCTTGACCCTGGAAGGTGCCACTCCCACTGTCCTTTCCTAATAAAATG
AGGAAATTGCATCGCATTGTCTGAGTAGGTGTCATTCTATTCTGGGGGGT
GGGGTGGGGCAGGACAGCAAGGGGGAGGATTGGGAAGACAATAGCAG
GCATGCTGGGGATGCGGTGGGCTCTATGGCTTCTGAGGCGGAAAGAAC
CAGCTGGGGCTCTAGGGGGTATCCCCACGCGCCCTGTAGCGGCGCATT
AAGCGCGGCGGGTGTGGTGGTTACGCGCAGCGTGACCGCTACACTTGC
CAGCGCCCTAGCGCCCGCTCCTTTCGCTTTCTTCCCTTCCTTTCTCGCCA
CGTTCGCCGGCTTTCCCCGTCAAGCTCTAAATCGGGGCATCCCTTTAGG
GTTCCGATTTAGTGCTTTACGGCACCTCGACCCCAAAAAACTTGATTAGG
GTGATGGTTCACGTAGTGGGCCATCGCCCTGATAGACGGTTTTTCGCCC
TTTGACGTTGGAGTCCACGTTCTTTAATAGTGGACTCTTGTTCCAAACTG
GAACAACACTCAACCCTATCTCGGTCTATTCTTTTGATTTATAAGGGATTT
TGGGGATTTCGGCCTATTGGTTAAAAAATGAGCTGATTTAACAAAAATTTA
ACGCGAATTAATTCTGTGGAATGTGTGTCAGTTAGGGTGTGGAAAGTCC
CCAGGCTCCCCAGGCAGGCAGAAGTATGCAAAGCATGCATCTCAATTAG
TCAGCAACCAGGTGTGGAAAGTCCCCAGGCTCCCCAGCAGGCAGAAGT
ATGCAAAGCATGCATCTCAATTAGTCAGCAACCATAGTCCCGCCCCTAAC
TCCGCCCATCCCGCCCCTAACTCCGCCCAGTTCCGCCCATTCTCCGCCC
CATGGCTGACTAATTTTTTTTATTTATGCAGAGGCCGAGGCCGCCTCTGC
CTCTGAGCTATTCCAGAAGTAGTGAGGAGGCTTTTTTGGAGGCCTAGGC
TTTTGCAAAAAGCTCCCGGGAGCTTGTATATCCATTTTCGGATCTGATCA
GCACGTGTTGACAATTAATCATCGGCATAGTATATCGGCATAGTATAATA
CGACAAGGTGAGGAACTAAACCATGGCCAAGTTGACCAGTGCCGTTCCG
GTGCTCACCGCGCGCGACGTCGCCGGAGCGGTCGAGTTCTGGACCGAC
CGGCTCGGGTTCTCCCGGGACTTCGTGGAGGACGACTTCGCCGGTGTG
GTCCGGGACGACGTGACCCTGTTCATCAGCGCGGTCCAGGACCAGGTG
GTGCCGGACAACACCCTGGCCTGGGTGTGGGTGCGCGGCCTGGACGA
GCTGTACGCCGAGTGGTCGGAGGTCGTGTCCACGAACTTCCGGGACGC
CTCCGGGCCGGCCATGACCGAGATCGGCGAGCAGCCGTGGGGGCGGG
AGTTCGCCCTGCGCGACCCGGCCGGCAACTGCGTGCACTTCGTGGCCG
AGGAGCAGGACTGACACGTGCTACGAGATTTCGATTCCACCGCCGCCTT
CTATGAAAGGTTGGGCTTCGGAATCGTTTTCCGGGACGCCGGCTGGATG
ATCCTCCAGCGCGGGGATCTCATGCTGGAGTTCTTCGCCCACCCCAACT
TGTTTATTGCAGCTTATAATGGTTACAAATAAAGCAATAGCATCACAAATT

TCACAAATAAAGCATTTTTTTCACTGCATTCTAGTTGTGGTTTGTCCAAAC
TCATCAATGTATCTTATCATGTCTGTATACCGTCGACCTCTAGCTAGAGC
TTGGCGTAATCATGGTCATAGCTGTTTCCTGTGTGAAATTGTTATCCGCT
CACAATTCCACACAACATACGAGCCGGAAGCATAAAGTGTAAAGCCTGG
GGTGCCTAATGAGTGAGCTAACTCACATTAATTGCGTTGCGCTCACTGC
CCGCTTTCCAGTCGGGAAACCTGTCGTGCCAGCTGCATTAATGAATCGG
CCAACGCGCGGGGAGAGGCGGTTTGCGTATTGGGCGCTCTTCCGCTTC
CTCGCTCACTGACTCGCTGCGCTCGGTCGTTCGGCTGCGGCGAGCGGT
ATCAGCTCACTCAAAGGCGGTAATACGGTTATCCACAGAATCAGGGGAT
AACGCAGGAAAGAACATGTGAGCAAAAGGCCAGCAAAAGGCCAGGAAC
CGTAAAAAGGCCGCGTTGCTGGCGTTTTTCCATAGGCTCCGCCCCCCTG
ACGAGCATCACAAAAATCGACGCTCAAGTCAGAGGTGGCGAAACCCGAC
AGGACTATAAAGATACCAGGCGTTTCCCCCTGGAAGCTCCCTCGTGCGC
TCTCCTGTTCCGACCCTGCCGCTTACCGGATACCTGTCCGCCTTTCTCC
CTTCGGGAAGCGTGGCGCTTTCTCAATGCTCACGCTGTAGGTATCTCAG
TTCGGTGTAGGTCGTTCGCTCCAAGCTGGGCTGTGTGCACGAACCCCCC
GTTCAGCCCGACCGCTGCGCCTTATCCGGTAACTATCGTCTTGAGTCCA
ACCCGGTAAGACACGACTTATCGCCACTGGCAGCAGCCACTGGTAACAG
GATTAGCAGAGCGAGGTATGTAGGCGGTGCTACAGAGTTCTTGAAGTGG
TGGCCTAACTACGGCTACACTAGAAGGACAGTATTTGGTATCTGCGCTCT
GCTGAAGCCAGTTACCTTCGGAAAAAGAGTTGGTAGCTCTTGATCCGGC
AAACAAACCACCGCTGGTAGCGGTGGTTTTTTTGTTTGCAAGCAGCAGAT
TACGCGCAGAAAAAAAGGATCTCAAGAAGATCCTTTGATCTTTTCTACGG
GGTCTGACGCTCAGTGGAACGAAAACTCACGTTAAGGGATTTTGGTCAT
GAGATTATCAAAAAGGATCTTCACCTAGATCCTTTTAAATTAAAAATGAAG
TTTTAAATCAATCTAAAGTATATATGAGTAAACTTGGTCTGACAGTTACCA
ATGCTTAATCAGTGAGGCACCTATCTCAGCGATCTGTCTATTTCGTTCAT
CCATAGTTGCCTGACTCCCCGTCGTGTAGATAACTACGATACGGGAGGG
CTTACCATCTGGCCCCAGTGCTGCAATGATACCGCGAGACCCACGCTCA
CCGGCTCCAGATTTATCAGCAATAAACCAGCCAGCCGGAAGGGCCGAG
CGCAGAAGTGGTCCTGCAACTTTATCCGCCTCCATCCAGTCTATTAATTG
TTGCCGGGAAGCTAGAGTAAGTAGTTCGCCAGTTAATAGTTTGCGCAAC
GTTGTTGCCATTGCTACAGGCATCGTGGTGTCACGCTCGTCGTTTGGTA
TGGCTTCATTCAGCTCCGGTTCCCAACGATCAAGGCGAGTTACATGATC
CCCCATGTTGTGCAAAAAAGCGGTTAGCTCCTTCGGTCCTCCGATCGTT
GTCAGAAGTAAGTTGGCCGCAGTGTTATCACTCATGGTTATGGCAGCAC
TGCATAATTCTCTTACTGTCATGCCATCCGTAAGATGCTTTTCTGTGACTG
GTGAGTACTCAACCAAGTCATTCTGAGAATAGTGTATGCGGCGACCGAG
TTGCTCTTGCCCGGCGTCAATACGGGATAATACCGCGCCACATAGCAGA
ACTTTAAAAGTGCTCATCATTGGAAAACGTTCTTCGGGGCGAAAACTCTC
AAGGATCTTACCGCTGTTGAGATCCAGTTCGATGTAACCCACTCGTGCA
CCCAACTGATCTTCAGCATCTTTTACTTTCACCAGCGTTTCTGGGTGAGC
AAAAACAGGAAGGCAAAATGCCGCAAAAAAGGGAATAAGGGCGACACG
GAAATGTTGAATACTCATACTCTTCCTTTTTCAATATTATTGAAGCATTTAT
CAGGGTTATTGTCTCATGAGCGGATACATATTTGAATGTATTTAGAAAAAT
AAACAAATAGGGGTTCCGCGCACATTTCCCCGAAAAGTGCCACCTGACG
TCGACGGATCGGGAGATCTCCCGATCCCCTATGGTCGACTCTCAGTACA
ATCTGCTCTGATGCCGCATAGTTAAGCCAGTATCTGCTCCCTGCTTGTGT
GTTGGAGGTCGCTGAGTAGTGCGCGAGCAAAATTTAAGCTACAACAAGG
CAAGGCTTGACCGACAATTGCATGAAGAATCTGCTTAGGGTTAGGCGTT

TTGCGCTGCTTCGCGATGTACGGGCCAGATATACGCGTTGACATTGATT
ATTGACTAGTTATTAATAGTAATCAATTACGGGGTCATTAGTTCATAGCCC
ATATATGGAGTTCCGCGTTACATAACTTACGGTAAATGGCCCGCCTGGCT
GACCGCCCAACGACCCCCGCCCATTGACGTCAATAATGACGTATGTTCC
CATAGTAACGCCAATAGGGACTTTCCATTGACGTCAATGGGTGGACTATT
TACGGTAAACTGCCCACTTGGCAGTACATCAAGTGTATCATATGCCAAGT
ACGCCCCCTATTGACGTCAATGACGGTAAATGGCCCGCCTGGCATTATG
CCCAGTACATGACCTTATGGGACTTTCCTACTTGGCAGTACATCTACGTA
TTAGTCATCGCTATTACCATGGTGATGCGGTTTTGGCAGTACATCAATGG
GCGTGGATAGCGGTTTGACTCACGGGGATTTCCAAGTCTCCACCCCATT
GACGTCAATGGGAGTTTGTTTTGGCACCAAAATCAACGGGACTTTCCAAA
ATGTCGTAACAACTCCGCCCCATTGACGCAAATGGGCGGTAGGCGTGTA
CGGTGGGAGGTCTATATAAGCAGAGCTCTCTGGCTAACTAGAGAACCCA
CTGCTTACTGGCTTATCGAAATTAATACGACTCACTATAGGGAGACCCAA
GCTGGCTAGCACCATGGAGCCCCCCAAGAAAAAGCGGAAAGTGCGGGT
TAACATGATCACCAGCGCCCTGCACAGAGCCGCCGATTGGGCCAAGAG
CGTGTTCTCTTCTGCCGCCCTGGGCGACCCTAGAAGAACCGCCAGACTC
GTGAATGTGGCCGCCCAGCTGGCCAAGTACAGCGGCAAGAGCATCACC
ATCAGCAGCGAGGGCAGCAAAGCCGCCCAGGAAGGCGCCTACCGGTTC
ATCAGAAACCCCAACGTGTCCGCCGAGGCCATCAGAAAGGCTGGCGCC
ATGCAGACCGTGAAGCTGGCCCAGGAATTCCCCGAGCTGCTGGCCATC
GAGGACACCACCAGCCTGAGCTACAGACACCAGGTGGCCGAGGAACTG
GGCAAGCTGGGCAGCATCCAGGACAAGAGCAGAGGGTGGTGGGTGCA
CAGCGTGCTGCTGCTGGAAGCCACCACCTTCAGAACCGTGGGCCTGCT
GCACCAGGAATGGTGGATGAGGCCCGACGATCCCGCCGACGCCGACGA
GAAAGAAAGCGGCAAATGGCTGGCCGCTGCCGCCACCAGCAGACTGAG
AATGGGCTCTATGATGAGCAACGTGATCGCCGTGTGCGACAGAGAGGC
CGACATCCACGCCTACCTGCAGGACAAACTGGCCCACAACGAGAGATTC
GTCGTGCGGAGCAAGCACCCCCGGAAGGATGTGGAAAGCGGCCTGTAC
CTGTACGACCACCTGAAGAACCAGCCCGAGCTGGGAGGCTACCAGATC
AGCATCCCCCAGAAAGGCGTGGTGGACAAGCGGGGCAAGCGGAAGAAC
AGACCCGCCAGAAAAGCCAGCCTGTCCCTGCGGAGCGGCCGGATCACA
CTGAAGCAGGGCAACATCACCCTGAACGCCGTGCTGGCCGAAGAGATC
AACCCCCCCAAGGGCGAGACACCCCTGAAGTGGCTGCTGCTGACAAGC
GAGCCCGTGGAATCTCTGGCTCAGGCCCTGAGAGTGATCGACATCTACA
CCCACAGATGGCGGATCGAGGAATTCCACAAGGCCTGGAAAACCGGCG
CTGGCGCCGAGCGGCAGAGAATGGAAGAACCCGACAACCTGGAACGGA
TGGTGTCCATCCTGAGCTTCGTGGCCGTGCGGCTGCTGCAGCTGAGAG
AGAGCTTTACCCCTCCACAGGCCCTGCGGGCTCAGGGACTGCTGAAAG
AAGCCGAGCACGTGGAAAGCCAGAGCGCCGAGACAGTGCTGACCCCCG
ATGAGTGTCAGCTGCTGGGCTACCTGGACAAGGGCAAACGCAAGCGCA
AAGAGAAGGCCGGCTCTCTGCAGTGGGCCTACATGGCCATTGCCCGGC
TGGGCGGATTCATGGACAGCAAGAGAACCGGAATCGCCTCCTGGGGAG
CCCTGTGGGAGGGATGGGAAGCCCTGCAGTCTAAGCTGGACGGCTTCC
TGGCCGCCAAGGACCTGATGGCCCAGGGCATTAAGATCGGGCCCAGCA
GAGGCGGCGGATCTGAAGGGGGAGGATCTGAGGGCGGAAGCGGCACT
AGTCAATTGATGATCACCTCTGCCCTGCATCGGGCCGCTGACTGGGCTA
AGTCCGTGTTTAGCAGTGCCGCTCTGGGAGATCCCAGGCGGACAGCTA
GACTCGTGAACGTGGCAGCTCAGCTGGCTAAATACTCCGGCAAGTCTAT
CACCATCTCCTCCGAGGGCTCCAAGGCTGCTCAGGAAGGGGCTTACAG

ATTCATCCGGAATCCTAATGTGTCTGCCGAAGCCATTCGGAAGGCAGGG
GCTATGCAGACAGTGAAACTGGCTCAGGAATTTCCAGAACTGCTGGCTA
TTGAAGATACCACCTCCCTGTCCTACCGGCATCAGGTGGCAGAAGAACT
GGGAAAACTGGGCTCTATCCAGGATAAGTCCCGGGGGTGGTGGGTGCA
CTCTGTGCTGCTGCTGGAAGCTACAACATTTCGGACAGTGGGACTGCTG
CATCAGGAATGGTGGATGCGCCCCGATGACCCTGCTGATGCAGATGAG
AAAGAATCTGGAAAGTGGCTGGCAGCCGCCGCTACCTCCCGGCTGCGG
ATGGGGAGCATGATGTCCAATGTGATTGCTGTGTGCGATCGGGAAGCCG
ATATTCATGCTTATCTGCAGGATAAGCTGGCTCATAATGAGCGCTTCGTC
GTGCGCTCCAAGCACCCTAGAAAGGACGTGGAATCCGGGCTGTATCTGT
ATGATCATCTGAAAAATCAGCCTGAACTGGGCGGGTATCAGATCTCTATT
CCTCAGAAAGGGGTGGTGGATAAGAGAGGGAAGCGCAAGAATCGGCCT
GCCCGGAAAGCCTCTCTGAGCCTGAGAAGCGGCAGAATTACCCTGAAAC
AGGGGAATATTACACTGAACGCTGTGCTGGCTGAGGAAATCAATCCTCC
TAAGGGCGAAACCCCTCTGAAATGGCTGCTGCTGACCTCCGAGCCTGTG
GAAAGCCTGGCCCAGGCACTGCGCGTGATCGATATCTATACACATCGGT
GGCGGATTGAAGAGTTTCACAAAGCTTGGAAAACAGGGGCAGGCGCTG
AGAGACAGCGGATGGAAGAACCTGATAATCTGGAAAGAATGGTGTCTAT
TCTGTCCTTTGTGGCTGTGCGCCTGCTGCAGCTGCGCGAGTCTTTTACA
CCACCCCAGGCTCTGAGAGCCCAGGGCCTGCTGAAAGAGGCTGAACAT
GTGGAATCCCAGTCCGCCGAAACCGTGCTGACACCTGACGAATGCCAG
CTGCTGGGATATCTGGATAAGGGGAAGCGGAAACGGAAAGAGAAAGCA
GGCAGCCTGCAGTGGGCTTATATGGCTATCGCCAGACTGGGGGGCTTT
ATGGACTCCAAGCGGACCGGCATTGCCTCTTGGGGCGCTCTGTGGGAA
GGCTGGGAGGCTCTGCAGTCCAAACTGGATGGATTTCTGGCCGCTAAAG
ATCTGATGGCTCAGGGGATCAAAATCCATCATCACCATCACCACCTCTAG
AGGGCCCGTTTAAACCCGCTGATCAGCCTCGA

pRC2148
ACGGGCCAGATATACGCGTTGACATTGATTATTGACTAGTTATTAATAGT
AATCAATTACGGGGTCATTAGTTCATAGCCCATATATGGAGTTCCGCGTT
ACATAACTTACGGTAAATGGCCCGCCTGGCTGACCGCCCAACGACCCCC
GCCCATTGACGTCAATAATGACGTATGTTCCCATAGTAACGCCAATAGGG
ACTTTCCATTGACGTCAATGGGTGGACTATTTACGGTAAACTGCCCACTT
GGCAGTACATCAAGTGTATCATATGCCAAGTACGCCCCCTATTGACGTCA
ATGACGGTAAATGGCCCGCCTGGCATTATGCCCAGTACATGACCTTATG
GGACTTTCCTACTTGGCAGTACATCTACGTATTAGTCATCGCTATTACCA
TGGTGATGCGGTTTTGGCAGTACATCAATGGGCGTGGATAGCGGTTTGA
CTCACGGGGATTTCCAAGTCTCCACCCCATTGACGTCAATGGGAGTTTG
TTTTGGCACCAAAATCAACGGGACTTTCCAAAATGTCGTAACAACTCCGC
CCCATTGACGCAAATGGGCGGTAGGCGTGTACGGTGGGAGGTCTATATA
AGCAGAGCTCTCTGGCTAACTAGAGAACCCACTGCTTACTGGCTTATCG
AAATTAATACGACTCACTATAGGGAGACCCAAGCTGGCTAGCACCATGG
AGCCCCCCAAGAAAAAGCGGAAAGTGCGGGTTAACATGATCACCAGCG
CCCTGCACAGAGCCGCCGATTGGGCCAAGAGCGTGTTCTCTTCTGCCG
CCCTGGGCGACCCTAGAAGAACCGCCAGACTCGTGAATGTGGCCGCCC
AGCTGGCCAAGTACAGCGGCAAGAGCATCACCATCAGCAGCGAGGGCA
GCAAAGCCGCCCAGGAAGGCGCCTACCGGTTCATCAGAAACCCCAACG
TGTCCGCCGAGGCCATCAGAAAGGCTGGCGCCATGCAGACCGTGAAGC
TGGCCCAGGAATTCCCCGAGCTGCTGGCCATCGAGGACACCACCAGCC

TGAGCTACAGACACCAGGTGGCCGAGGAACTGGGCAAGCTGGGCAGCA
TCCAGGACAAGAGCAGAGGGTGGTGGGTGCACAGCGTGCTGCTGCTGG
AAGCCACCACCTTCAGAACCGTGGGCCTGCTGCACCAGGAATGGTGGA
TGAGGCCCGACGATCCCGCCGACGCCGACGAGAAAGAAAGCGGCAAAT
GGCTGGCCGCTGCCGCCACCAGCAGACTGAGAATGGGCTCTATGATGA
GCAACGTGATCGCCGTGTGCGACAGAGAGGCCGACATCCACGCCTACC
TGCAGGACAAACTGGCCCACAACGAGAGATTCGTCGTGCGGAGCAAGC
ACCCCCGGAAGGATGTGGAAAGCGGCCTGTACCTGTACGACCACCTGA
AGAACCAGCCCGAGCTGGGAGGCTACCAGATCAGCATCCCCCAGAAAG
GCGTGGTGGACAAGCGGGGCAAGCGGAAGAACAGACCCGCCAGAAAA
GCCAGCCTGTCCCTGCGGAGCGGCCGGATCACACTGAAGCAGGGCAAC
ATCACCCTGAACGCCGTGCTGGCCGAAGAGATCAACCCCCCCAAGGGC
GAGACACCCCTGAAGTGGCTGCTGCTGACAAGCGAGCCCGTGGAATCT
CTGGCTCAGGCCCTGAGAGTGATCGACATCTACACCCACAGATGGCGG
ATCGAGGAATTCCACAAGGCCTGGAAAACCGGCGCTGGCGCCGAGCGG
CAGAGAATGGAAGAACCCGACAACCTGGAACGGATGGTGTCCATCCTGA
GCTTCGTGGCCGTGCGGCTGCTGCAGCTGAGAGAGAGCTTTACCCCTC
CACAGGCCCTGCGGGCTCAGGGACTGCTGAAAGAAGCCGAGCACGTGG
AAAGCCAGAGCGCCGAGACAGTGCTGACCCCCGATGAGTGTCAGCTGC
TGGGCTACCTGGACAAGGGCAAACGCAAGCGCAAAGAGAAGGCCGGCT
CTCTGCAGTGGGCCTACATGGCCATTGCCCGGCTGGGCGGATTCATGG
ACAGCAAGAGAACCGGAATCGCCTCCTGGGGAGCCCTGTGGGAGGGAT
GGGAAGCCCTGCAGTCTAAGCTGGACGGCTTCCTGGCCGCCAAGGACC
TGATGGCCCAGGGCATTAAGATCCATCATCACCATCACCACCTCTAGAG
GGCCCGTTTAAACCCGCTGATCAGCCTCGACTGTGCCTTCTAGTTGCCA
GCCATCTGTTGTTTGCCCCTCCCCCGTGCCTTCCTTGACCCTGGAAGGT
GCCACTCCCACTGTCCTTTCCTAATAAAATGAGGAAATTGCATCGCATTG
TCTGAGTAGGTGTCATTCTATTCTGGGGGGTGGGGTGGGGCAGGACAG
CAAGGGGGAGGATTGGGAAGACAATAGCAGGCATGCTGGGGATGCGGT
GGGCTCTATGGCTTCTGAGGCGGAAAGAACCAGCTGGGGCTCTAGGGG
GTATCCCCACGCGCCCTGTAGCGGCGCATTAAGCGCGGCGGGTGTGGT
GGTTACGCGCAGCGTGACCGCTACACTTGCCAGCGCCCTAGCGCCCGC
TCCTTTCGCTTTCTTCCCTTCCTTTCTCGCCACGTTCGCCGGCTTTCCCC
GTCAAGCTCTAAATCGGGGCATCCCTTTAGGGTTCCGATTTAGTGCTTTA
CGGCACCTCGACCCCAAAAAACTTGATTAGGGTGATGGTTCACGTAGTG
GGCCATCGCCCTGATAGACGGTTTTTCGCCCTTTGACGTTGGAGTCCAC
GTTCTTTAATAGTGGACTCTTGTTCCAAACTGGAACAACACTCAACCCTA
TCTCGGTCTATTCTTTTGATTTATAAGGGATTTTGGGGATTTCGGCCTATT
GGTTAAAAAATGAGCTGATTTAACAAAAATTTAACGCGAATTAATTCTGTG
GAATGTGTGTCAGTTAGGGTGTGGAAAGTCCCCAGGCTCCCCAGGCAG
GCAGAAGTATGCAAAGCATGCATCTCAATTAGTCAGCAACCAGGTGTGG
AAAGTCCCCAGGCTCCCCAGCAGGCAGAAGTATGCAAAGCATGCATCTC
AATTAGTCAGCAACCATAGTCCCGCCCCTAACTCCGCCCATCCCGCCCC
TAACTCCGCCCAGTTCCGCCCATTCTCCGCCCCATGGCTGACTAATTTTT
TTTATTTATGCAGAGGCCGAGGCCGCCTCTGCCTCTGAGCTATTCCAGA
AGTAGTGAGGAGGCTTTTTTGGAGGCCTAGGCTTTTGCAAAAAGCTCCC
GGGAGCTTGTATATCCATTTTCGGATCTGATCAGCACGTGTTGACAATTA
ATCATCGGCATAGTATATCGGCATAGTATAATACGACAAGGTGAGGAACT
AAACCATGGCCAAGTTGACCAGTGCCGTTCCGGTGCTCACCGCGCGCG
ACGTCGCCGGAGCGGTCGAGTTCTGGACCGACCGGCTCGGGTTCTCCC

GGGACTTCGTGGAGGACGACTTCGCCGGTGTGGTCCGGGACGACGTGA
CCCTGTTCATCAGCGCGGTCCAGGACCAGGTGGTGCCGGACAACACCC
TGGCCTGGGTGTGGGTGCGCGGCCTGGACGAGCTGTACGCCGAGTGG
TCGGAGGTCGTGTCCACGAACTTCCGGGACGCCTCCGGGCCGGCCATG
ACCGAGATCGGCGAGCAGCCGTGGGGGCGGGAGTTCGCCCTGCGCGA
CCCGGCCGGCAACTGCGTGCACTTCGTGGCCGAGGAGCAGGACTGACA
CGTGCTACGAGATTTCGATTCCACCGCCGCCTTCTATGAAAGGTTGGGC
TTCGGAATCGTTTTCCGGGACGCCGGCTGGATGATCCTCCAGCGCGGG
GATCTCATGCTGGAGTTCTTCGCCCACCCCAACTTGTTTATTGCAGCTTA
TAATGGTTACAAATAAAGCAATAGCATCACAAATTTCACAAATAAAGCATT
TTTTTCACTGCATTCTAGTTGTGGTTTGTCCAAACTCATCAATGTATCTTA
TCATGTCTGTATACCGTCGACCTCTAGCTAGAGCTTGGCGTAATCATGGT
CATAGCTGTTTCCTGTGTGAAATTGTTATCCGCTCACAATTCCACACAAC
ATACGAGCCGGAAGCATAAAGTGTAAAGCCTGGGGTGCCTAATGAGTGA
GCTAACTCACATTAATTGCGTTGCGCTCACTGCCCGCTTTCCAGTCGGG
AAACCTGTCGTGCCAGCTGCATTAATGAATCGGCCAACGCGCGGGGAG
AGGCGGTTTGCGTATTGGGCGCTCTTCCGCTTCCTCGCTCACTGACTCG
CTGCGCTCGGTCGTTCGGCTGCGGCGAGCGGTATCAGCTCACTCAAAG
GCGGTAATACGGTTATCCACAGAATCAGGGGATAACGCAGGAAAGAACA
TGTGAGCAAAAGGCCAGCAAAAGGCCAGGAACCGTAAAAAGGCCGCGT
TGCTGGCGTTTTTCCATAGGCTCCGCCCCCCTGACGAGCATCACAAAAA
TCGACGCTCAAGTCAGAGGTGGCGAAACCCGACAGGACTATAAAGATAC
CAGGCGTTTCCCCCTGGAAGCTCCCTCGTGCGCTCTCCTGTTCCGACCC
TGCCGCTTACCGGATACCTGTCCGCCTTTCTCCCTTCGGGAAGCGTGGC
GCTTTCTCAATGCTCACGCTGTAGGTATCTCAGTTCGGTGTAGGTCGTTC
GCTCCAAGCTGGGCTGTGTGCACGAACCCCCCGTTCAGCCCGACCGCT
GCGCCTTATCCGGTAACTATCGTCTTGAGTCCAACCCGGTAAGACACGA
CTTATCGCCACTGGCAGCAGCCACTGGTAACAGGATTAGCAGAGCGAG
GTATGTAGGCGGTGCTACAGAGTTCTTGAAGTGGTGGCCTAACTACGGC
TACACTAGAAGGACAGTATTTGGTATCTGCGCTCTGCTGAAGCCAGTTAC
CTTCGGAAAAAGAGTTGGTAGCTCTTGATCCGGCAAACAAACCACCGCT
GGTAGCGGTGGTTTTTTTGTTTGCAAGCAGCAGATTACGCGCAGAAAAA
AAGGATCTCAAGAAGATCCTTTGATCTTTTCTACGGGGTCTGACGCTCAG
TGGAACGAAAACTCACGTTAAGGGATTTTGGTCATGAGATTATCAAAAAG
GATCTTCACCTAGATCCTTTTAAATTAAAAATGAAGTTTTAAATCAATCTAA
AGTATATATGAGTAAACTTGGTCTGACAGTTACCAATGCTTAATCAGTGA
GGCACCTATCTCAGCGATCTGTCTATTTCGTTCATCCATAGTTGCCTGAC
TCCCCGTCGTGTAGATAACTACGATACGGGAGGGCTTACCATCTGGCCC
CAGTGCTGCAATGATACCGCGAGACCCACGCTCACCGGCTCCAGATTTA
TCAGCAATAAACCAGCCAGCCGGAAGGGCCGAGCGCAGAAGTGGTCCT
GCAACTTTATCCGCCTCCATCCAGTCTATTAATTGTTGCCGGGAAGCTAG
AGTAAGTAGTTCGCCAGTTAATAGTTTGCGCAACGTTGTTGCCATTGCTA
CAGGCATCGTGGTGTCACGCTCGTCGTTTGGTATGGCTTCATTCAGCTC
CGGTTCCCAACGATCAAGGCGAGTTACATGATCCCCCATGTTGTGCAAA
AAAGCGGTTAGCTCCTTCGGTCCTCCGATCGTTGTCAGAAGTAAGTTGG
CCGCAGTGTTATCACTCATGGTTATGGCAGCACTGCATAATTCTCTTACT
GTCATGCCATCCGTAAGATGCTTTTCTGTGACTGGTGAGTACTCAACCAA
GTCATTCTGAGAATAGTGTATGCGGCGACCGAGTTGCTCTTGCCCGGCG
TCAATACGGGATAATACCGCGCCACATAGCAGAACTTTAAAAGTGCTCAT
CATTGGAAAACGTTCTTCGGGGCGAAAACTCTCAAGGATCTTACCGCTG

TTGAGATCCAGTTCGATGTAACCCACTCGTGCACCCAACTGATCTTCAGC
ATCTTTTACTTTCACCAGCGTTTCTGGGTGAGCAAAAACAGGAAGGCAAA
ATGCCGCAAAAAAGGGAATAAGGGCGACACGGAAATGTTGAATACTCAT
ACTCTTCCTTTTTCAATATTATTGAAGCATTTATCAGGGTTATTGTCTCAT
GAGCGGATACATATTTGAATGTATTTAGAAAAATAAACAAATAGGGGTTC
CGCGCACATTTCCCCGAAAAGTGCCACCTGACGTCGACGGATCGGGAG
ATCTCCCGATCCCCTATGGTCGACTCTCAGTACAATCTGCTCTGATGCCG
CATAGTTAAGCCAGTATCTGCTCCCTGCTTGTGTGTTGGAGGTCGCTGA
GTAGTGCGCGAGCAAAATTTAAGCTACAACAAGGCAAGGCTTGACCGAC
AATTGCATGAAGAATCTGCTTAGGGTTAGGCGTTTTGCGCTGCTTCGCG
ATGT

pRC2152
GGGTGCTTTGCCAAGGGTACCAATGTTTTAATGGCGGATGGGTCTATTG
AATGTATTGAAAACATTGAGGTTGGTAATAAGGTCATGGGTAAAGATGGC
AGACCTCGTGAGGTAATTAAATTGCCCAGAGGAAGAGAAACTATGTACA
GCGTCGTGCAGAAAAGTCAGCACAGAGCCCACAAAAGTGACTCAAGTCG
TGAAGTGCCAGAATTACTCAAGTTTACGTGTAATGCGACCCATGAGTTGG
TTGTTAGAACACCTCGTAGTGTCCGCCGTTTGTCTCGTACCATTAAGGGT
GTCGAATATTTTGAAGTTATTACTTTTGAGATGGGCCAAAAGAAAGCCCC
CGACGGTAGAATTGTTGAGCTTGTCAAGGAAGTTTCAAAGAGCTACCCA
ATATCTGAGGGGCCTGAGAGAGCCAACGAATTAGTAGAATCCTATAGAA
AGGCTTCAAATAAAGCTTATTTTGAGTGGACTATTGAGGCCAGAGATCTT
TCTCTGTTGGGTTCCCATGTTCGTAAAGCTACCTACCAGACTTACGCTCC
AATTCTTTATGAGAATGACCACTTTTTCGACTACATGCAAAAAAGTAAGTT
TCATCTCACCATTGAAGGTCCAAAAGTACTTGCTTATTTACTTGGTTTATG
GATTGGTGATGGATTGTCTGACAGGGCAACTTTTTCGGTTGATTCCAGAG
ATACTTCTTTGATGGAACGTGTTACTGAATATGCTGAAAAGTTGAATTTGT
GCGCCGAGTATAAGGACAGAAAAGAACCACAAGTTGCCAAAACTGTTAA
TTTGTACTCTAAAGTTGTCAGAGGTAATGGTATTCGCAATAATCTTAATAC
TGAGAATCCATTATGGGACGCTATTGTTGGCTTAGGATTCTTGAAGGACG
GTGTCAAAAATATTCCTTCTTTCTTGTCTACGGACAATATCGGTACTCGTG
AAACATTTCTTGCTGGTCTAATTGATTCTGATGGCTATGTTACTGATGAGC
ATGGTATTAAAGCAACAATAAAGACAATTCATACTTCTGTCAGAGATGGTT
TGGTTTCCCTTGCTCGTTCTTTAGGCTTAGTAGTCTCGGTTAACGCAGAA
CCTGCTAAGGTTGACATGAATGTCACCAAACATAAAATTAGTTATGCTATT
TATATGTCTGGTGGAGATGTTTTGCTTAACGTTCTTTCGAAGTGTGCCGG
CTCTAAAAAATTCAGGCCTGCTCCCGCCGCTGCTTTTGCACGTGAGTGC
CGCGGATTTTATTTCGAGTTACAAGAATTGAAGGAAGACGATTATTATGG
GATTACTTTATCTGATGATTCTGATCATCAGTTTTTGCTTGGATCCCAGGT
TGTCGTCCATGCATGCGGTGGCCTGACCGGTCTGAACTCAGGCCTCAC
GACAAATCCTGGTGTATCCGCTTGGCAGGTCAACACAGCTTATACTGCG
GGACAATTGGTCACATATAACGGCAAGACGTATAAATGTTTGCAGCCCCA
CACCTCCTTGGCAGGATGGGAACCATCCAACGTTCCTGCCTTGTGGCAG
CTTCAATGACTGCAGGAAGGGGATCCGGCTGCTAACAAAGCCCGAAAG
GAAGCTGAGTTGGCTGCTGCCACCGCTGAGCAATAACTAGCATAACCCC
TTGGGGCCTCTAAACGGGTCTTGAGGGGTTTTTTGCTGAAAGGAGGAAC
TATATCCGGATAACTACGTCAGGTGGCACTTTTCGGGGAAATGTGCGCG
GAACCCCTATTTGTTTATTTTTCTAAATACATTCAAATATGTATCCGCTCAT
GAGACAATAACCCTGATAAATGCTTCAATAATATTGAAAAAGGAAGAGTA

TGAGTATTCAACATTTCCGTGTCGCCCTTATTCCCTTTTTTGCGGCATTTT
GCCTTCCTGTTTTTGCTCACCCAGAAACGCTGGTGAAAGTAAAAGATGCT
GAAGATCAGTTGGGTGCACGAGTGGGTTACATCGAACTGGATCTCAACA
GCGGTAAGATCCTTGAGAGTTTTCGCCCCGAAGAACGTTCTCCAATGAT
GAGCACTTTTAAAGTTCTGCTATGTGGCGCGGTATTATCCCGTGTTGACG
CCGGGCAAGAGCAACTCGGTCGCCGCATACACTATTCTCAGAATGACTT
GGTTGAGTACTCACCAGTCACAGAAAAGCATCTTACGGATGGCATGACA
GTAAGAGAATTATGCAGTGCTGCCATAACCATGAGTGATAACACTGCGG
CCAACTTACTTCTGACAACGATCGGAGGACCGAAGGAGCTAACCGCTTT
TTTGCACAACATGGGGGATCATGTAACTCGCCTTGATCGTTGGGAACCG
GAGCTGAATGAAGCCATACCAAACGACGAGCGTGACACCACGATGCCTG
TAGCAATGGCAACAACGTTGCGCAAACTATTAACTGGCGAACTACTTACT
CTAGCTTCCCGGCAACAATTAATAGACTGGATGGAGGCGGATAAAGTTG
CAGGACCACTTCTGCGCTCGGCCCTTCCGGCTGGCTGGTTTATTGCTGA
TAAATCTGGAGCCGGTGAGCGTGGGTCTCGCGGTATCATTGCAGCACTG
GGGCCAGATGGTAAGCCCTCCCGTATCGTAGTTATCTACACGACGGGGA
GTCAGGCAACTATGGATGAACGAAATAGACAGATCGCTGAGATAGGTGC
CTCACTGATTAAGCATTGGTAACTGTCAGACCAAGTTTACTCATATATACT
TTAGATTGATTTACCCCGGTTGATAATCAGAAAAGCCCCAAAAACAGGAA
GATTGTATAAGCAAATATTTAAATTGTAAACGTTAATATTTTGTTAAAATTC
GCGTTAAATTTTTGTTAAATCAGCTCATTTTTTAACCAATAGGCCGAAATC
GGCAAAATCCCTTATAAATCAAAAGAATAGCCCGAGATAGGGTTGAGTGT
TGTTCCAGTTTGGAACAAGAGTCCACTATTAAAGAACGTGGACTCCAACG
TCAAAGGGCGAAAAACCGTCTATCAGGGCGATGGCCCACTACGTGAACC
ATCACCCAAATCAAGTTTTTTGGGGTCGAGGTGCCGTAAAGCACTAAATC
GGAACCCTAAAGGGAGCCCCCGATTTAGAGCTTGACGGGGAAAGCCGG
CGAACGTGGCGAGAAAGGAAGGGAAGAAAGCGAAAGGAGCGGGCGCT
AGGGCGCTGGCAAGTGTAGCGGTCACGCTGCGCGTAACCACCACACCC
GCCGCGCTTAATGCGCCGCTACAGGGCGCGTAAAAGGATCTAGGTGAA
GATCCTTTTTGATAATCTCATGACCAAAATCCCTTAACGTGAGTTTTCGTT
CCACTGAGCGTCAGACCCCGTAGAAAAGATCAAAGGATCTTCTTGAGAT
CCTTTTTTTCTGCGCGTAATCTGCTGCTTGCAAACAAAAAAACCACCGCT
ACCAGCGGTGGTTTGTTTGCCGGATCAAGAGCTACCAACTCTTTTTCCGA
AGGTAACTGGCTTCAGCAGAGCGCAGATACCAAATACTGTCCTTCTAGT
GTAGCCGTAGTTAGGCCACCACTTCAAGAACTCTGTAGCACCGCCTACA
TACCTCGCTCTGCTAATCCTGTTACCAGTGGCTGCTGCCAGTGGCGATA
AGTCGTGTCTTACCGGGTTGGACTCAAGACGATAGTTACCGGATAAGGC
GCAGCGGTCGGGCTGAACGGGGGGTTCGTGCACACAGCCCAGCTTGGA
GCGAACGACCTACACCGAACTGAGATACCTACAGCGTGAGCTATGAGAA
AGCGCCACGCTTCCCGAAGGGAGAAAGGCGGACAGGTATCCGGTAAGC
GGCAGGGTCGGAACAGGAGAGCGCACGAGGGAGCTTCCAGGGGGAAA
CGCCTGGTATCTTTATAGTCCTGTCGGGTTTCGCCACCTCTGACTTGAGC
GTCGATTTTTGTGATGCTCGTCAGGGGGGCGGAGCCTATGGAAAAACGC
CAGCAACGCGGCCTTTTTACGGTTCCTGGCCTTTTGCTGGCCTTTTGCTC
ACATGTTCTTTCCTGCGTTATCCCCTGATTCTGTGGATAACCGTATTACC
GCCTTTGAGTGAGCTGATACCGCTCGCCGCAGCCGAACGACCGAGCGC
AGCGAGTCAGTGAGCGAGGAAGCTATGGTGCACTCTCAGTACAATCTGC
TCTGATGCCGCATAGTTAAGCCAGTATACACTCCGCTATCGCTACGTGAC
TGGGTCATGGCTGCGCCCCGACACCCGCCAACACCCGCTGACGCGCCC
TGACGGGCTTGTCTGCTCCCGGCATCCGCTTACAGACAAGCTGTGACCG

TCTCCGGGAGCTGCATGTGTCAGAGGTTTTCACCGTCATCACCGAAACG
CGCGAGGCAGCTGCGGTAAAGCTCATCAGCGTGGTCGTGCAGCGATTC
ACAGATGTCTGCCTGTTCATCCGCGTCCAGCTCGTTGAGTTTCTCCAGAA
GCGTTAATGTCTGGCTTCTGATAAAGCGGGCCATGTTAAGGGCGGTTTT
TTCCTGTTTGGTCACTGATGCCTCCGTGTAAGGGGGATTTCTGTTCATGG
GGGTAATGATACCGATGAAACGAGAGAGGATGCTCACGATACGGGTTAC
TGATGATGAACATGCCCGGTTACTGGAACGTTGTGAGGGTAAACAACTG
GCGGTATGGATGCGGCGGGACCAGAGAAAAATCACTCAGGGTCAATGC
CAGCCGAACGCCAGCAAGACGTAGCCCAGCGCGTCGGCCGCCATGCC
GGCGATAATGGCCTGCTTCTCGCCGAAACGTTTGGTGGCGGGACCAGT
GACGAAGGCTTGAGCGAGGGCGTGCAAGATTCCGAATACCGCAAGCGA
CAGGCCGATCATCGTCGCGCTCCAGCGAAAGCGGTCCTCGCCGAAAAT
GACCCAGAGCGCTGCCGGCACCTGTCCTACGAGTTGCATGATAAAGAAG
ACAGTCATAAGTGCGGCGACGATAGTCATGCCCCGCGCCCACCGGAAG
GAGCTGACTGGGTTGAAGGCTCTCAAGGGCATCGGTCGAGATCCCGGT
GCCTAATGAGTGAGCTAACTTACATTAATTGCGTTGCGCTCACTGCCCGC
TTTCCAGTCGGGAAACCTGTCGTGCCAGCTGCATTAATGAATCGGCCAA
CGCGCGGGGAGAGGCGGTTTGCGTATTGGGCGCCAGGGTGGTTTTTCT
TTTCACCAGTGAGACGGGCAACAGCTGATTGCCCTTCACCGCCTGGCCC
TGAGAGAGTTGCAGCAAGCGGTCCACGCTGGTTTGCCCCAGCAGGCGA
AAATCCTGTTTGATGGTGGTTAACGGCGGGATATAACATGAGCTGTCTTC
GGTATCGTCGTATCCCACTACCGAGATATCCGCACCAACGCGCAGCCCG
GACTCGGTAATGGCGCGCATTGCGCCCAGCGCCATCTGATCGTTGGCA
ACCAGCATCGCAGTGGGAACGATGCCCTCATTCAGCATTTGCATGGTTT
GTTGAAAACCGGACATGGCACTCCAGTCGCCTTCCCGTTCCGCTATCGG
CTGAATTTGATTGCGAGTGAGATATTTATGCCAGCCAGCCAGACGCAGA
CGCGCCGAGACAGAACTTAATGGGCCCGCTAACAGCGCGATTTGCTGG
TGACCCAATGCGACCAGATGCTCCACGCCCAGTCGCGTACCGTCTTCAT
GGGAGAAAATAATACTGTTGATGGGTGTCTGGTCAGAGACATCAAGAAA
TAACGCCGGAACATTAGTGCAGGCAGCTTCCACAGCAATGGCATCCTGG
TCATCCAGCGGATAGTTAATGATCAGCCCACTGACGCGTTGCGCGAGAA
GATTGTGCACCGCCGCTTTACAGGCTTCGACGCCGCTTCGTTCTACCAT
CGACACCACCACGCTGGCACCCAGTTGATCGGCGCGAGATTTAATCGCC
GCGACAATTTGCGACGGCGCGTGCAGGGCCAGACTGGAGGTGGCAACG
CCAATCAGCAACGACTGTTTGCCCGCCAGTTGTTGTGCCACGCGGTTGG
GAATGTAATTCAGCTCCGCCATCGCCGCTTCCACTTTTTCCCGCGTTTTC
GCAGAAACGTGGCTGGCCTGGTTCACCACGCGGGAAACGGTCTGATAA
GAGACACCGGCATACTCTGCGACATCGTATAACGTTACTGGTTTCACATT
CACCACCCTGAATTGACTCTCTTCCGGGCGCTATCATGCCATACCGCGA
AAGGTTTTGCGCCATTCGATGGTGTCCGGGATCTCGACGCTCTCCCTTA
TGCGACTCCTGCATTAGGAAGCAGCCCAGTAGTAGGTTGAGGCCGTTGA
GCACCGCCGCCGCAAGGAATGGTGCATGCCGGCATGCCGCCCTTTCGT
CTTCAAGAATTAATTCCCAATTCCCCAGGCATCAAATAAAACGAAAGGCT
CAGTCGAAAGACTGGGCCTTTCGTTTTATCTGTTGTTTGTCGGTGAACGC
TCTCCTGAGTAGGACAAATCCGCCGGGAGCGGATTTGAACGTTGCGAAG
CAACGGCCCGGAGGGTGGCGGGCAGGACGCCCGCCATAAACTGCCAG
GAATTAATTCCCCAGGCATCAAATAAAACGAAAGGCTCAGTCGAAAGACT
GGGCCTTTCGTTTTATCTGTTGTTTGTCGGTGAACGCTCTCCTGAGTAGG
ACAAATCCGCCGGGAGCGGATTTGAACGTTGCGAAGCAACGGCCCGGA
GGGTGGCGGGCAGGACGCCCGCCATAAACTGCCAGGAATTAATTCCCC

AGGCATCAAATAAAACGAAAGGCTCAGTCGAAAGACTGGGCCTTTCGTT
TTATCTGTTGTTTGTCGGTGAACGCTCTCCTGAGTAGGACAAATCCGCCG
GGAGCGGATTTGAACGTTGCGAAGCAACGGCCCGGAGGGTGGCGGGC
AGGACGCCCGCCATAAACTGCCAGGAATTAATTCCCCAGGCATCAAATA
AAACGAAAGGCTCAGTCGAAAGACTGGGCCTTTCGTTTTATCTGTTGTTT
GTCGGTGAACGCTCTCCTGAGTAGGACAAATCCGCCGGGAGCGGATTT
GAACGTTGCGAAGCAACGGCCCGGAGGGTGGCGGGCAGGACGCCCGC
CATAAACTGCCAGGAATTAATTCCCCAGGCATCAAATAAAACGAAAGGCT
CAGTCGAAAGACTGGGCCTTTCGTTTTATCTGTTGTTTGTCGGTGAACGC
TCTCCTGAGTAGGACAAATCCGCCGGGAGCGGATTTGAACGTTGCGAAG
CAACGGCCCGGAGGGTGGCGGGCAGGACGCCCGCCATAAACTGCCAG
GAATTGGGGATCGGAATTAATTCCCGGTTTAAACCGGGGATCTCGATCC
CGCGAAATTAATACGACTCACTATAGGGGAATTGTGAGCGGATAACAATT
CCCCTCTAGAAATAATTTTGTTTAACTTTAAGAAGGAGATATACATATGGC
TAGCTCGCGAATGATAACTTCTGCTCTTCATCGTGCGGCCGACTGGGCT
AAATCTGTGTTCTCTTCGGCGGCGCTGGGTGATCCTCGCCGTACTGCCC
GCTTGGTTAACGTCGCCGCCCAATTGGCAAAATATTCTGGTAAATCAATA
ACCATCTCATCAGAGGGTAGTAAGGCCGCACAGGAAGGCGCTTACCGAT
TTATCCGCAATCCCAACGTTTCTGCCGAGGCGATCAGAAAGGCTGGCGC
CATGCAAACAGTCAAGTTGGCTCAGGAGTTTCCCGAACTGCTGGCCATT
GAGGACACCACCTCTTTGAGTTATCGCCACCAGGTCGCCGAAGAGCTTG
GCAAGCTGGGCTCTATTCAGGATAAATCCCGCGGATGGTGGGTTCACTC
CGTTCTCTTGCTCGAGGCCACCACATTCCGCACCGTAGGATTACTGCAT
CAGGAGTGGTGGATGCGCCCGGATGACCCTGCCGATGCGGATGAAAAG
GAGAGTGGCAAATGGCTGGCAGCGGCCGCAACTAGCCGGTTACGCATG
GGCAGCATGATGAGCAACGTGATTGCGGTCTGTGACCGCGAAGCCGAT
ATTCATGCTTATCTGCAGGACAAACTGGCGCATAACGAGCGCTTCGTGG
TGCGCTCCAAGCACCCACGCAAGGACGTAGAGTCTGGGTTGTATCTGTA
CGACCATCTGAAGAACCAACCGGAGTTGGGTGGCTATCAGATCAGCATT
CCGCAAAAGGGCGTGGTGGATAAACGCGGTAAACGTAAAAATCGACCAG
CCCGCAAGGCGAGCTTGAGCCTGCGCAGTGGGCGCATCACGCTAAAAC
AGGGGAATATCACGCTCAACGCGGTGCTGGCCGAGGAGATTAACCCGC
CCAAGGGTGAGACCCCGTTGAAATGGTTGTTGCTGACCAGCGAACCGGT
CGAGTCGCTAGCCCAAGCCTTGCGCGTCATCGACATTTATACCCATCGC
TGGCGGATCGAGGAGTTCCATAAGGCATGGAAAACCGGAGCAGGAGCC
GAGAGGCAACGCATGGAGGAGCCGGATAATCTGGAGCGGATGGTCTCG
ATCCTCTCGTTTGTTGCGGTCAGGCTGTTACAGCTCAGAGAAAGCTTCAC
GCCGCCGCAAGCACTCAGGGCGCAAGGGCTGCTAAAGGAAGCGGAACA
CGTAGAAAGCCAGTCCGCAGAAACGGTGCTGACCCCGGATGAATGTCA
GCTACTGGGCTATCTGGACAAGGGAAAACGCAAGCGCAAAGAGAAAGC
AGGTAGCTTGCAGTGGGCTTACATGGCGATAGCTAGACTGGGCGGTTTT
ATGGACAGCAAGCGAACCGGAATTGCCAGCTGGGGCGCCCTCTGGGAA
GGTTGGGAAGCCCTGCAAAGTAAACTGGATGACTTTCTTGCCGCCAAGG
ATCTGATGGCGCAGGGGATCAAGATCGGATCCTCTAGAGGAGGTGGCT
CAGAAGGTGGCGGATCTGAAGGTGGCTCTGGAACTAGTGAATTCATGAT
AACTTCTGCTCTTCATCGTGCGGCCGACTGGGCTAAATCTGTGTTCTCTT
CGGCGGCGCTGGGTGATCCTCGCCGTACTGCCCGCTTGGTTAACGTCG
CCGCCCAATTGGCAAAATATTCTGGTAAATCAATAACCATCTCATCAGAG
GGTAGTAAGGCCGCACAGGAAGGCGCTTACCGATTTATCCGCAATCCCA
ACGTTTCTGCCGAGGCGATCAGAAAGGCTGGCGCCATGCAAACAGTCAA

GTTGGCTCAGGAGTTTCCCGAACTGCTGGCCATTGAGGACACCACCTCT
TTGAGTTATCGCCACCAGGTCGCCGAAGAGCTTGGCAAGCTGGGCTCTA
TTCAGGATAAATCCCGCGGATGGTGGGTTCACTCCGTTCTCTTGCTCGA
GGCCACCACATTCCGCACCGTAGGATTACTGCATCAGGAGTGGTGGATG
CGCCCGGATGACCCTGCCGATGCGGATGAAAAGGAGAGTGGCAAATGG
CTGGCAGCGGCCGCAACTAGCCGGTTACGCATGGGCAGCATGATGAGC
AACGTGATTGCGGTCTGTGACCGCGAAGCCGATATTCATGCTTATCTGC
AGGACAAACTGGCGCATAACGAGCGCTTCGTGGTGCGCTCCAAGCACC
CACGCAAGGACGTAGAGTCTGGGTTGTATCTGTACGACCATCTGAAGAA
CCAACCGGAGTTGGGTGGCTATCAGATCAGCATTCCGCAAAAGGGCGT
GGTGGATAAACGCGGTAAACGTAAAAATCGACCAGCCCGCAAGGCGAG
CTTGAGCCTGCGCAGTGGGCGCATCACGCTAAAACAGGGGAATATCAC
GCTCAACGCGGTGCTGGCCGAGGAGATTAACCCGCCCAAGGGTGAGAC
CCCGTTGAAATGGTTGTTGCTGACCAGCGAACCGGTCGAGTCGCTAGCC
CAAGCCTTGCGCGTCATCGACATTTATACCCATCGCTGGCGGATCGAGG
AGTTCCATAAGGCATGGAAAACCGGAGCAGGAGCCGAGAGGCAACGCA
TGGAGGAGCCGGATAATCTGGAGCGGATGGTCTCGATCCTCTCGTTTGT
TGCGGTCAGGCTGTTACAGCTCAGAGAAAGCTTCACGCCGCCGCAAGC
ACTCAGGGCGCAAGGGCTGCTAAAGGAAGCGGAACACGTAGAAAGCCA
GTCCGCAGAAACGGTGCTGACCCCGGATGAATGTCAGCTACTGGGCTAT
CTGGACAAGGGAAAACGCAAGCGCAAAGAGAAAGCAGGTAGCTTGCAG
TGGGCTTACATGGCGATAGCTAGACTGGGCGGTTTTATGGACAGCAAGC
GAACCGGAATTGCCAGCTGGGGCGCCCTCTGGGAAGGTTGGGAAGCCC
TGCAAAGTAAACTGGATGGCTTTCTTGCCGCCAAGGATCTGATGGCGCA
GGGGATCAAGATCCCC

pRC2153
GGGTGCTTTGCCAAGGGTACCAATGTTTTAATGGCGGATGGGTCTATTG
AATGTATTGAAAACATTGAGGTTGGTAATAAGGTCATGGGTAAAGATGGC
AGACCTCGTGAGGTAATTAAATTGCCCAGAGGAAGAGAAACTATGTACA
GCGTCGTGCAGAAAAGTCAGCACAGAGCCCACAAAAGTGACTCAAGTCG
TGAAGTGCCAGAATTACTCAAGTTTACGTGTAATGCGACCCATGAGTTGG
TTGTTAGAACACCTCGTAGTGTCCGCCGTTTGTCTCGTACCATTAAGGGT
GTCGAATATTTTGAAGTTATTACTTTTGAGATGGGCCAAAAGAAAGCCCC
CGACGGTAGAATTGTTGAGCTTGTCAAGGAAGTTTCAAAGAGCTACCCA
ATATCTGAGGGGCCTGAGAGAGCCAACGAATTAGTAGAATCCTATAGAA
AGGCTTCAAATAAAGCTTATTTTGAGTGGACTATTGAGGCCAGAGATCTT
TCTCTGTTGGGTTCCCATGTTCGTAAAGCTACCTACCAGACTTACGCTCC
AATTCTTTATGAGAATGACCACTTTTTCGACTACATGCAAAAAAGTAAGTT
TCATCTCACCATTGAAGGTCCAAAAGTACTTGCTTATTTACTTGGTTTATG
GATTGGTGATGGATTGTCTGACAGGGCAACTTTTTCGGTTGATTCCAGAG
ATACTTCTTTGATGGAACGTGTTACTGAATATGCTGAAAAGTTGAATTTGT
GCGCCGAGTATAAGGACAGAAAAGAACCACAAGTTGCCAAAACTGTTAA
TTTGTACTCTAAAGTTGTCAGAGGTAATGGTATTCGCAATAATCTTAATAC
TGAGAATCCATTATGGGACGCTATTGTTGGCTTAGGATTCTTGAAGGACG
GTGTCAAAAATATTCCTTCTTTCTTGTCTACGGACAATATCGGTACTCGTG
AAACATTTCTTGCTGGTCTAATTGATTCTGATGGCTATGTTACTGATGAGC
ATGGTATTAAAGCAACAATAAAGACAATTCATACTTCTGTCAGAGATGGTT
TGGTTTCCCTTGCTCGTTCTTTAGGCTTAGTAGTCTCGGTTAACGCAGAA
CCTGCTAAGGTTGACATGAATGTCACCAAACATAAAATTAGTTATGCTATT

TATATGTCTGGTGGAGATGTTTTGCTTAACGTTCTTTCGAAGTGTGCCGG
CTCTAAAAAATTCAGGCCTGCTCCCGCCGCTGCTTTTGCACGTGAGTGC
CGCGGATTTTATTTCGAGTTACAAGAATTGAAGGAAGACGATTATTATGG
GATTACTTTATCTGATGATTCTGATCATCAGTTTTTGCTTGGATCCCAGGT
TGTCGTCCATGCATGCGGTGGCCTGACCGGTCTGAACTCAGGCCTCAC
GACAAATCCTGGTGTATCCGCTTGGCAGGTCAACACAGCTTATACTGCG
GGACAATTGGTCACATATAACGGCAAGACGTATAAATGTTTGCAGCCCCA
CACCTCCTTGGCAGGATGGGAACCATCCAACGTTCCTGCCTTGTGGCAG
CTTCAATGACTGCAGGAAGGGGATCCGGCTGCTAACAAAGCCCGAAAG
GAAGCTGAGTTGGCTGCTGCCACCGCTGAGCAATAACTAGCATAACCCC
TTGGGGCCTCTAAACGGGTCTTGAGGGGTTTTTTGCTGAAAGGAGGAAC
TATATCCGGATAACTACGTCAGGTGGCACTTTTCGGGGAAATGTGCGCG
GAACCCCTATTTGTTTATTTTTCTAAATACATTCAAATATGTATCCGCTCAT
GAGACAATAACCCTGATAAATGCTTCAATAATATTGAAAAAGGAAGAGTA
TGAGTATTCAACATTTCCGTGTCGCCCTTATTCCCTTTTTTGCGGCATTTT
GCCTTCCTGTTTTTGCTCACCCAGAAACGCTGGTGAAAGTAAAAGATGCT
GAAGATCAGTTGGGTGCACGAGTGGGTTACATCGAACTGGATCTCAACA
GCGGTAAGATCCTTGAGAGTTTTCGCCCCGAAGAACGTTCTCCAATGAT
GAGCACTTTTAAAGTTCTGCTATGTGGCGCGGTATTATCCCGTGTTGACG
CCGGGCAAGAGCAACTCGGTCGCCGCATACACTATTCTCAGAATGACTT
GGTTGAGTACTCACCAGTCACAGAAAAGCATCTTACGGATGGCATGACA
GTAAGAGAATTATGCAGTGCTGCCATAACCATGAGTGATAACACTGCGG
CCAACTTACTTCTGACAACGATCGGAGGACCGAAGGAGCTAACCGCTTT
TTTGCACAACATGGGGGATCATGTAACTCGCCTTGATCGTTGGGAACCG
GAGCTGAATGAAGCCATACCAAACGACGAGCGTGACACCACGATGCCTG
TAGCAATGGCAACAACGTTGCGCAAACTATTAACTGGCGAACTACTTACT
CTAGCTTCCCGGCAACAATTAATAGACTGGATGGAGGCGGATAAAGTTG
CAGGACCACTTCTGCGCTCGGCCCTTCCGGCTGGCTGGTTTATTGCTGA
TAAATCTGGAGCCGGTGAGCGTGGGTCTCGCGGTATCATTGCAGCACTG
GGGCCAGATGGTAAGCCCTCCCGTATCGTAGTTATCTACACGACGGGGA
GTCAGGCAACTATGGATGAACGAAATAGACAGATCGCTGAGATAGGTGC
CTCACTGATTAAGCATTGGTAACTGTCAGACCAAGTTTACTCATATATACT
TTAGATTGATTTACCCCGGTTGATAATCAGAAAAGCCCCAAAAACAGGAA
GATTGTATAAGCAAATATTTAAATTGTAAACGTTAATATTTTGTTAAAATTC
GCGTTAAATTTTTGTTAAATCAGCTCATTTTTTAACCAATAGGCCGAAATC
GGCAAAATCCCTTATAAATCAAAAGAATAGCCCGAGATAGGGTTGAGTGT
TGTTCCAGTTTGGAACAAGAGTCCACTATTAAAGAACGTGGACTCCAACG
TCAAAGGGCGAAAAACCGTCTATCAGGGCGATGGCCCACTACGTGAACC
ATCACCCAAATCAAGTTTTTTGGGGTCGAGGTGCCGTAAAGCACTAAATC
GGAACCCTAAAGGGAGCCCCCGATTTAGAGCTTGACGGGGAAAGCCGG
CGAACGTGGCGAGAAAGGAAGGGAAGAAAGCGAAAGGAGCGGGCGCT
AGGGCGCTGGCAAGTGTAGCGGTCACGCTGCGCGTAACCACCACACCC
GCCGCGCTTAATGCGCCGCTACAGGGCGCGTAAAAGGATCTAGGTGAA
GATCCTTTTTGATAATCTCATGACCAAAATCCCTTAACGTGAGTTTTCGTT
CCACTGAGCGTCAGACCCCGTAGAAAAGATCAAAGGATCTTCTTGAGAT
CCTTTTTTTCTGCGCGTAATCTGCTGCTTGCAAACAAAAAAACCACCGCT
ACCAGCGGTGGTTTGTTTGCCGGATCAAGAGCTACCAACTCTTTTTCCGA
AGGTAACTGGCTTCAGCAGAGCGCAGATACCAAATACTGTCCTTCTAGT
GTAGCCGTAGTTAGGCCACCACTTCAAGAACTCTGTAGCACCGCCTACA
TACCTCGCTCTGCTAATCCTGTTACCAGTGGCTGCTGCCAGTGGCGATA

AGTCGTGTCTTACCGGGTTGGACTCAAGACGATAGTTACCGGATAAGGC
GCAGCGGTCGGGCTGAACGGGGGGTTCGTGCACACAGCCCAGCTTGGA
GCGAACGACCTACACCGAACTGAGATACCTACAGCGTGAGCTATGAGAA
AGCGCCACGCTTCCCGAAGGGAGAAAGGCGGACAGGTATCCGGTAAGC
GGCAGGGTCGGAACAGGAGAGCGCACGAGGGAGCTTCCAGGGGGAAA
CGCCTGGTATCTTTATAGTCCTGTCGGGTTTCGCCACCTCTGACTTGAGC
GTCGATTTTTGTGATGCTCGTCAGGGGGGCGGAGCCTATGGAAAAACGC
CAGCAACGCGGCCTTTTTACGGTTCCTGGCCTTTTGCTGGCCTTTTGCTC
ACATGTTCTTTCCTGCGTTATCCCCTGATTCTGTGGATAACCGTATTACC
GCCTTTGAGTGAGCTGATACCGCTCGCCGCAGCCGAACGACCGAGCGC
AGCGAGTCAGTGAGCGAGGAAGCTATGGTGCACTCTCAGTACAATCTGC
TCTGATGCCGCATAGTTAAGCCAGTATACACTCCGCTATCGCTACGTGAC
TGGGTCATGGCTGCGCCCCGACACCCGCCAACACCCGCTGACGCGCCC
TGACGGGCTTGTCTGCTCCCGGCATCCGCTTACAGACAAGCTGTGACCG
TCTCCGGGAGCTGCATGTGTCAGAGGTTTTCACCGTCATCACCGAAACG
CGCGAGGCAGCTGCGGTAAAGCTCATCAGCGTGGTCGTGCAGCGATTC
ACAGATGTCTGCCTGTTCATCCGCGTCCAGCTCGTTGAGTTTCTCCAGAA
GCGTTAATGTCTGGCTTCTGATAAAGCGGGCCATGTTAAGGGCGGTTTT
TTCCTGTTTGGTCACTGATGCCTCCGTGTAAGGGGGATTTCTGTTCATGG
GGGTAATGATACCGATGAAACGAGAGAGGATGCTCACGATACGGGTTAC
TGATGATGAACATGCCCGGTTACTGGAACGTTGTGAGGGTAAACAACTG
GCGGTATGGATGCGGCGGGACCAGAGAAAAATCACTCAGGGTCAATGC
CAGCCGAACGCCAGCAAGACGTAGCCCAGCGCGTCGGCCGCCATGCC
GGCGATAATGGCCTGCTTCTCGCCGAAACGTTTGGTGGCGGGACCAGT
GACGAAGGCTTGAGCGAGGGCGTGCAAGATTCCGAATACCGCAAGCGA
CAGGCCGATCATCGTCGCGCTCCAGCGAAAGCGGTCCTCGCCGAAAAT
GACCCAGAGCGCTGCCGGCACCTGTCCTACGAGTTGCATGATAAAGAAG
ACAGTCATAAGTGCGGCGACGATAGTCATGCCCCGCGCCCACCGGAAG
GAGCTGACTGGGTTGAAGGCTCTCAAGGGCATCGGTCGAGATCCCGGT
GCCTAATGAGTGAGCTAACTTACATTAATTGCGTTGCGCTCACTGCCCGC
TTTCCAGTCGGGAAACCTGTCGTGCCAGCTGCATTAATGAATCGGCCAA
CGCGCGGGGAGAGGCGGTTTGCGTATTGGGCGCCAGGGTGGTTTTTCT
TTTCACCAGTGAGACGGGCAACAGCTGATTGCCCTTCACCGCCTGGCCC
TGAGAGAGTTGCAGCAAGCGGTCCACGCTGGTTTGCCCCAGCAGGCGA
AAATCCTGTTTGATGGTGGTTAACGGCGGGATATAACATGAGCTGTCTTC
GGTATCGTCGTATCCCACTACCGAGATATCCGCACCAACGCGCAGCCCG
GACTCGGTAATGGCGCGCATTGCGCCCAGCGCCATCTGATCGTTGGCA
ACCAGCATCGCAGTGGGAACGATGCCCTCATTCAGCATTTGCATGGTTT
GTTGAAAACCGGACATGGCACTCCAGTCGCCTTCCCGTTCCGCTATCGG
CTGAATTTGATTGCGAGTGAGATATTTATGCCAGCCAGCCAGACGCAGA
CGCGCCGAGACAGAACTTAATGGGCCCGCTAACAGCGCGATTTGCTGG
TGACCCAATGCGACCAGATGCTCCACGCCCAGTCGCGTACCGTCTTCAT
GGGAGAAAATAATACTGTTGATGGGTGTCTGGTCAGAGACATCAAGAAA
TAACGCCGGAACATTAGTGCAGGCAGCTTCCACAGCAATGGCATCCTGG
TCATCCAGCGGATAGTTAATGATCAGCCCACTGACGCGTTGCGCGAGAA
GATTGTGCACCGCCGCTTTACAGGCTTCGACGCCGCTTCGTTCTACCAT
CGACACCACCACGCTGGCACCCAGTTGATCGGCGCGAGATTTAATCGCC
GCGACAATTTGCGACGGCGCGTGCAGGGCCAGACTGGAGGTGGCAACG
CCAATCAGCAACGACTGTTTGCCCGCCAGTTGTTGTGCCACGCGGTTGG
GAATGTAATTCAGCTCCGCCATCGCCGCTTCCACTTTTTCCCGCGTTTTC

GCAGAAACGTGGCTGGCCTGGTTCACCACGCGGGAAACGGTCTGATAA
GAGACACCGGCATACTCTGCGACATCGTATAACGTTACTGGTTTCACATT
CACCACCCTGAATTGACTCTCTTCCGGGCGCTATCATGCCATACCGCGA
AAGGTTTTGCGCCATTCGATGGTGTCCGGGATCTCGACGCTCTCCCTTA
TGCGACTCCTGCATTAGGAAGCAGCCCAGTAGTAGGTTGAGGCCGTTGA
GCACCGCCGCCGCAAGGAATGGTGCATGCCGGCATGCCGCCCTTTCGT
CTTCAAGAATTAATTCCCAATTCCCCAGGCATCAAATAAAACGAAAGGCT
CAGTCGAAAGACTGGGCCTTTCGTTTTATCTGTTGTTTGTCGGTGAACGC
TCTCCTGAGTAGGACAAATCCGCCGGGAGCGGATTTGAACGTTGCGAAG
CAACGGCCCGGAGGGTGGCGGGCAGGACGCCCGCCATAAACTGCCAG
GAATTAATTCCCCAGGCATCAAATAAAACGAAAGGCTCAGTCGAAAGACT
GGGCCTTTCGTTTTATCTGTTGTTTGTCGGTGAACGCTCTCCTGAGTAGG
ACAAATCCGCCGGGAGCGGATTTGAACGTTGCGAAGCAACGGCCCGGA
GGGTGGCGGGCAGGACGCCCGCCATAAACTGCCAGGAATTAATTCCCC
AGGCATCAAATAAAACGAAAGGCTCAGTCGAAAGACTGGGCCTTTCGTT
TTATCTGTTGTTTGTCGGTGAACGCTCTCCTGAGTAGGACAAATCCGCCG
GGAGCGGATTTGAACGTTGCGAAGCAACGGCCCGGAGGGTGGCGGGC
AGGACGCCCGCCATAAACTGCCAGGAATTAATTCCCCAGGCATCAAATA
AAACGAAAGGCTCAGTCGAAAGACTGGGCCTTTCGTTTTATCTGTTGTTT
GTCGGTGAACGCTCTCCTGAGTAGGACAAATCCGCCGGGAGCGGATTT
GAACGTTGCGAAGCAACGGCCCGGAGGGTGGCGGGCAGGACGCCCGC
CATAAACTGCCAGGAATTAATTCCCCAGGCATCAAATAAAACGAAAGGCT
CAGTCGAAAGACTGGGCCTTTCGTTTTATCTGTTGTTTGTCGGTGAACGC
TCTCCTGAGTAGGACAAATCCGCCGGGAGCGGATTTGAACGTTGCGAAG
CAACGGCCCGGAGGGTGGCGGGCAGGACGCCCGCCATAAACTGCCAG
GAATTGGGGATCGGAATTAATTCCCGGTTTAAACCGGGGATCTCGATCC
CGCGAAATTAATACGACTCACTATAGGGGAATTGTGAGCGGATAACAATT
CCCCTCTAGAAATAATTTTGTTTAACTTTAAGAAGGAGATATACATATGGC
TAGCTCGCGAATGATAACTTCTGCTCTTCATCGTGCGGCCGACTGGGCT
AAATCTGTGTTCTCTTCGGCGGCGCTGGGTGATCCTCGCCGTACTGCCC
GCTTGGTTAACGTCGCCGCCCAATTGGCAAAATATTCTGGTAAATCAATA
ACCATCTCATCAGAGGGTAGTAAGGCCGCACAGGAAGGCGCTTACCGAT
TTATCCGCAATCCCAACGTTTCTGCCGAGGCGATCAGAAAGGCTGGCGC
CATGCAAACAGTCAAGTTGGCTCAGGAGTTTCCCGAACTGCTGGCCATT
GAGGACACCACCTCTTTGAGTTATCGCCACCAGGTCGCCGAAGAGCTTG
GCAAGCTGGGCTCTATTCAGGATAAATCCCGCGGATGGTGGGTTCACTC
CGTTCTCTTGCTCGAGGCCACCACATTCCGCACCGTAGGATTACTGCAT
CAGGAGTGGTGGATGCGCCCGGATGACCCTGCCGATGCGGATGAAAAG
GAGAGTGGCAAATGGCTGGCAGCGGCCGCAACTAGCCGGTTACGCATG
GGCAGCATGATGAGCAACGTGATTGCGGTCTGTGACCGCGAAGCCGAT
ATTCATGCTTATCTGCAGGACAAACTGGCGCATAACGAGCGCTTCGTGG
TGCGCTCCAAGCACCCACGCAAGGACGTAGAGTCTGGGTTGTATCTGTA
CGACCATCTGAAGAACCAACCGGAGTTGGGTGGCTATCAGATCAGCATT
CCGCAAAAGGGCGTGGTGGATAAACGCGGTAAACGTAAAAATCGACCAG
CCCGCAAGGCGAGCTTGAGCCTGCGCAGTGGGCGCATCACGCTAAAAC
AGGGGAATATCACGCTCAACGCGGTGCTGGCCGAGGAGATTAACCCGC
CCAAGGGTGAGACCCCGTTGAAATGGTTGTTGCTGACCAGCGAACCGGT
CGAGTCGCTAGCCCAAGCCTTGCGCGTCATCGACATTTATACCCATCGC
TGGCGGATCGAGGAGTTCCATAAGGCATGGAAAACCGGAGCAGGAGCC
GAGAGGCAACGCATGGAGGAGCCGGATAATCTGGAGCGGATGGTCTCG

ATCCTCTCGTTTGTTGCGGTCAGGCTGTTACAGCTCAGAGAAAGCTTCAC
GCCGCCGCAAGCACTCAGGGCGCAAGGGCTGCTAAAGGAAGCGGAACA
CGTAGAAAGCCAGTCCGCAGAAACGGTGCTGACCCCGGATGAATGTCA
GCTACTGGGCTATCTGGACAAGGGAAAACGCAAGCGCAAAGAGAAAGC
AGGTAGCTTGCAGTGGGCTTACATGGCGATAGCTAGACTGGGCGGTTTT
ATGGACAGCAAGCGAACCGGAATTGCCAGCTGGGGCGCCCTCTGGGAA
GGTTGGGAAGCCCTGCAAAGTAAACTGGATGGCTTTCTTGCCGCCAAGG
ATCTGATGGCGCAGGGGATCAAGATCGGATCCTCTAGAGGAGGTGGCT
CAGAAGGTGGCGGATCTGAAGGTGGCTCTGGAACTAGTGAATTCATGAT
AACTTCTGCTCTTCATCGTGCGGCCGACTGGGCTAAATCTGTGTTCTCTT
CGGCGGCGCTGGGTGATCCTCGCCGTACTGCCCGCTTGGTTAACGTCG
CCGCCCAATTGGCAAAATATTCTGGTAAATCAATAACCATCTCATCAGAG
GGTAGTAAGGCCGCACAGGAAGGCGCTTACCGATTTATCCGCAATCCCA
ACGTTTCTGCCGAGGCGATCAGAAAGGCTGGCGCCATGCAAACAGTCAA
GTTGGCTCAGGAGTTTCCCGAACTGCTGGCCATTGAGGCCACCACCTCT
TTGAGTTATCGCCACCAGGTCGCCGAAGAGCTTGGCAAGCTGGGCTCTA
TTCAGGATAAATCCCGCGGATGGTGGGTTCACTCCGTTCTCTTGCTCGA
GGCCACCACATTCCGCACCGTAGGATTACTGCATCAGGAGTGGTGGATG
CGCCCGGATGACCCTGCCGATGCGGATGAAAAGGAGAGTGGCAAATGG
CTGGCAGCGGCCGCAACTAGCCGGTTACGCATGGGCAGCATGATGAGC
AACGTGATTGCGGTCTGTGACCGCGAAGCCGATATTCATGCTTATCTGC
AGGACAAACTGGCGCATAACGAGCGCTTCGTGGTGCGCTCCAAGCACC
CACGCAAGGACGTAGAGTCTGGGTTGTATCTGTACGACCATCTGAAGAA
CCAACCGGAGTTGGGTGGCTATCAGATCAGCATTCCGCAAAAGGGCGT
GGTGGATAAACGCGGTAAACGTAAAAATCGACCAGCCCGCAAGGCGAG
CTTGAGCCTGCGCAGTGGGCGCATCACGCTAAAACAGGGGAATATCAC
GCTCAACGCGGTGCTGGCCGAGGAGATTAACCCGCCCAAGGGTGAGAC
CCCGTTGAAATGGTTGTTGCTGACCAGCGAACCGGTCGAGTCGCTAGCC
CAAGCCTTGCGCGTCATCGACATTTATACCCATCGCTGGCGGATCGAGG
AGTTCCATAAGGCATGGAAAACCGGAGCAGGAGCCGAGAGGCAACGCA
TGGAGGAGCCGGATAATCTGGAGCGGATGGTCTCGATCCTCTCGTTTGT
TGCGGTCAGGCTGTTACAGCTCAGAGAAAGCTTCACGCCGCCGCAAGC
ACTCAGGGCGCAAGGGCTGCTAAAGGAAGCGGAACACGTAGAAAGCCA
GTCCGCAGAAACGGTGCTGACCCCGGATGAATGTCAGCTACTGGGCTAT
CTGGACAAGGGAAAACGCAAGCGCAAAGAGAAAGCAGGTAGCTTGCAG
TGGGCTTACATGGCGATAGCTAGACTGGGCGGTTTTATGGACAGCAAGC
GAACCGGAATTGCCAGCTGGGGCGCCCTCTGGGAAGGTTGGGAAGCCC
TGCAAAGTAAACTGGATGACTTTCTTGCCGCCAAGGATCTGATGGCGCA
GGGGATCAAGATCCCC

pRC2163
CTGTGCCTTCTAGTTGCCAGCCATCTGTTGTTTGCCCCTCCCCCGTGCC
TTCCTTGACCCTGGAAGGTGCCACTCCCACTGTCCTTTCCTAATAAAATG
AGGAAATTGCATCGCATTGTCTGAGTAGGTGTCATTCTATTCTGGGGGGT
GGGGTGGGGCAGGACAGCAAGGGGGAGGATTGGGAAGACAATAGCAG
GCATGCTGGGGATGCGGTGGGCTCTATGGCTTCTGAGGCGGAAAGAAC
CAGCTGGGGCTCTAGGGGGTATCCCCACGCGCCCTGTAGCGGCGCATT
AAGCGCGGCGGGTGTGGTGGTTACGCGCAGCGTGACCGCTACACTTGC
CAGCGCCCTAGCGCCCGCTCCTTTCGCTTTCTTCCCTTCCTTTCTCGCCA
CGTTCGCCGGCTTTCCCCGTCAAGCTCTAAATCGGGGCATCCCTTTAGG

GTTCCGATTTAGTGCTTTACGGCACCTCGACCCCAAAAAACTTGATTAGG
GTGATGGTTCACGTAGTGGGCCATCGCCCTGATAGACGGTTTTTCGCCC
TTTGACGTTGGAGTCCACGTTCTTTAATAGTGGACTCTTGTTCCAAACTG
GAACAACACTCAACCCTATCTCGGTCTATTCTTTTGATTTATAAGGGATTT
TGGGGATTTCGGCCTATTGGTTAAAAAATGAGCTGATTTAACAAAAATTTA
ACGCGAATTAATTCTGTGGAATGTGTGTCAGTTAGGGTGTGGAAAGTCC
CCAGGCTCCCCAGGCAGGCAGAAGTATGCAAAGCATGCATCTCAATTAG
TCAGCAACCAGGTGTGGAAAGTCCCCAGGCTCCCCAGCAGGCAGAAGT
ATGCAAAGCATGCATCTCAATTAGTCAGCAACCATAGTCCCGCCCCTAAC
TCCGCCCATCCCGCCCCTAACTCCGCCCAGTTCCGCCCATTCTCCGCCC
CATGGCTGACTAATTTTTTTTATTTATGCAGAGGCCGAGGCCGCCTCTGC
CTCTGAGCTATTCCAGAAGTAGTGAGGAGGCTTTTTTGGAGGCCTAGGC
TTTTGCAAAAAGCTCCCGGGAGCTTGTATATCCATTTTCGGATCTGATCA
GCACGTGTTGACAATTAATCATCGGCATAGTATATCGGCATAGTATAATA
CGACAAGGTGAGGAACTAAACCATGGCCAAGTTGACCAGTGCCGTTCCG
GTGCTCACCGCGCGCGACGTCGCCGGAGCGGTCGAGTTCTGGACCGAC
CGGCTCGGGTTCTCCCGGGACTTCGTGGAGGACGACTTCGCCGGTGTG
GTCCGGGACGACGTGACCCTGTTCATCAGCGCGGTCCAGGACCAGGTG
GTGCCGGACAACACCCTGGCCTGGGTGTGGGTGCGCGGCCTGGACGA
GCTGTACGCCGAGTGGTCGGAGGTCGTGTCCACGAACTTCCGGGACGC
CTCCGGGCCGGCCATGACCGAGATCGGCGAGCAGCCGTGGGGGCGGG
AGTTCGCCCTGCGCGACCCGGCCGGCAACTGCGTGCACTTCGTGGCCG
AGGAGCAGGACTGACACGTGCTACGAGATTTCGATTCCACCGCCGCCTT
CTATGAAAGGTTGGGCTTCGGAATCGTTTTCCGGGACGCCGGCTGGATG
ATCCTCCAGCGCGGGGATCTCATGCTGGAGTTCTTCGCCCACCCCAACT
TGTTTATTGCAGCTTATAATGGTTACAAATAAAGCAATAGCATCACAAATT
TCACAAATAAAGCATTTTTTTCACTGCATTCTAGTTGTGGTTTGTCCAAAC
TCATCAATGTATCTTATCATGTCTGTATACCGTCGACCTCTAGCTAGAGC
TTGGCGTAATCATGGTCATAGCTGTTTCCTGTGTGAAATTGTTATCCGCT
CACAATTCCACACAACATACGAGCCGGAAGCATAAAGTGTAAAGCCTGG
GGTGCCTAATGAGTGAGCTAACTCACATTAATTGCGTTGCGCTCACTGC
CCGCTTTCCAGTCGGGAAACCTGTCGTGCCAGCTGCATTAATGAATCGG
CCAACGCGCGGGGAGAGGCGGTTTGCGTATTGGGCGCTCTTCCGCTTC
CTCGCTCACTGACTCGCTGCGCTCGGTCGTTCGGCTGCGGCGAGCGGT
ATCAGCTCACTCAAAGGCGGTAATACGGTTATCCACAGAATCAGGGGAT
AACGCAGGAAAGAACATGTGAGCAAAAGGCCAGCAAAAGGCCAGGAAC
CGTAAAAAGGCCGCGTTGCTGGCGTTTTTCCATAGGCTCCGCCCCCCTG
ACGAGCATCACAAAAATCGACGCTCAAGTCAGAGGTGGCGAAACCCGAC
AGGACTATAAAGATACCAGGCGTTTCCCCCTGGAAGCTCCCTCGTGCGC
TCTCCTGTTCCGACCCTGCCGCTTACCGGATACCTGTCCGCCTTTCTCC
CTTCGGGAAGCGTGGCGCTTTCTCAATGCTCACGCTGTAGGTATCTCAG
TTCGGTGTAGGTCGTTCGCTCCAAGCTGGGCTGTGTGCACGAACCCCCC
GTTCAGCCCGACCGCTGCGCCTTATCCGGTAACTATCGTCTTGAGTCCA
ACCCGGTAAGACACGACTTATCGCCACTGGCAGCAGCCACTGGTAACAG
GATTAGCAGAGCGAGGTATGTAGGCGGTGCTACAGAGTTCTTGAAGTGG
TGGCCTAACTACGGCTACACTAGAAGGACAGTATTTGGTATCTGCGCTCT
GCTGAAGCCAGTTACCTTCGGAAAAAGAGTTGGTAGCTCTTGATCCGGC
AAACAAACCACCGCTGGTAGCGGTGGTTTTTTTGTTTGCAAGCAGCAGAT
TACGCGCAGAAAAAAAGGATCTCAAGAAGATCCTTTGATCTTTTCTACGG
GGTCTGACGCTCAGTGGAACGAAAACTCACGTTAAGGGATTTTGGTCAT

GAGATTATCAAAAAGGATCTTCACCTAGATCCTTTTAAATTAAAAATGAAG
TTTTAAATCAATCTAAAGTATATATGAGTAAACTTGGTCTGACAGTTACCA
ATGCTTAATCAGTGAGGCACCTATCTCAGCGATCTGTCTATTTCGTTCAT
CCATAGTTGCCTGACTCCCCGTCGTGTAGATAACTACGATACGGGAGGG
CTTACCATCTGGCCCCAGTGCTGCAATGATACCGCGAGACCCACGCTCA
CCGGCTCCAGATTTATCAGCAATAAACCAGCCAGCCGGAAGGGCCGAG
CGCAGAAGTGGTCCTGCAACTTTATCCGCCTCCATCCAGTCTATTAATTG
TTGCCGGGAAGCTAGAGTAAGTAGTTCGCCAGTTAATAGTTTGCGCAAC
GTTGTTGCCATTGCTACAGGCATCGTGGTGTCACGCTCGTCGTTTGGTA
TGGCTTCATTCAGCTCCGGTTCCCAACGATCAAGGCGAGTTACATGATC
CCCCATGTTGTGCAAAAAAGCGGTTAGCTCCTTCGGTCCTCCGATCGTT
GTCAGAAGTAAGTTGGCCGCAGTGTTATCACTCATGGTTATGGCAGCAC
TGCATAATTCTCTTACTGTCATGCCATCCGTAAGATGCTTTTCTGTGACTG
GTGAGTACTCAACCAAGTCATTCTGAGAATAGTGTATGCGGCGACCGAG
TTGCTCTTGCCCGGCGTCAATACGGGATAATACCGCGCCACATAGCAGA
ACTTTAAAAGTGCTCATCATTGGAAAACGTTCTTCGGGGCGAAAACTCTC
AAGGATCTTACCGCTGTTGAGATCCAGTTCGATGTAACCCACTCGTGCA
CCCAACTGATCTTCAGCATCTTTTACTTTCACCAGCGTTTCTGGGTGAGC
AAAAACAGGAAGGCAAAATGCCGCAAAAAAGGGAATAAGGGCGACACG
GAAATGTTGAATACTCATACTCTTCCTTTTTCAATATTATTGAAGCATTTAT
CAGGGTTATTGTCTCATGAGCGGATACATATTTGAATGTATTTAGAAAAAT
AAACAAATAGGGGTTCCGCGCACATTTCCCCGAAAAGTGCCACCTGACG
TCGACGGATCGGGAGATCTCCCGATCCCCTATGGTCGACTCTCAGTACA
ATCTGCTCTGATGCCGCATAGTTAAGCCAGTATCTGCTCCCTGCTTGTGT
GTTGGAGGTCGCTGAGTAGTGCGCGAGCAAAATTTAAGCTACAACAAGG
CAAGGCTTGACCGACAATTGCATGAAGAATCTGCTTAGGGTTAGGCGTT
TTGCGCTGCTTCGCGATGTACGGGCCAGATATACGCGTTGACATTGATT
ATTGACTAGTTATTAATAGTAATCAATTACGGGGTCATTAGTTCATAGCCC
ATATATGGAGTTCCGCGTTACATAACTTACGGTAAATGGCCCGCCTGGCT
GACCGCCCAACGACCCCCGCCCATTGACGTCAATAATGACGTATGTTCC
CATAGTAACGCCAATAGGGACTTTCCATTGACGTCAATGGGTGGACTATT
TACGGTAAACTGCCCACTTGGCAGTACATCAAGTGTATCATATGCCAAGT
ACGCCCCCTATTGACGTCAATGACGGTAAATGGCCCGCCTGGCATTATG
CCCAGTACATGACCTTATGGGACTTTCCTACTTGGCAGTACATCTACGTA
TTAGTCATCGCTATTACCATGGTGATGCGGTTTTGGCAGTACATCAATGG
GCGTGGATAGCGGTTTGACTCACGGGGATTTCCAAGTCTCCACCCCATT
GACGTCAATGGGAGTTTGTTTTGGCACCAAAATCAACGGGACTTTCCAAA
ATGTCGTAACAACTCCGCCCCATTGACGCAAATGGGCGGTAGGCGTGTA
CGGTGGGAGGTCTATATAAGCAGAGCTCTCTGGCTAACTAGAGAACCCA
CTGCTTACTGGCTTATCGAAATTAATACGACTCACTATAGGGAGACCCAA
GCTGGCTAGCACCATGGAGCCCCCCAAGAAAAAGCGGAAAGTGCGGGT
TATGGTGAGCAAGGGCGAGGAGCTGTTCACCGGGGTGGTGCCCATCCT
GGTCGAGCTGGACGGCGACGTAAACGGCCACAAGTTCAGCGTGCGCGG
CGAGGGCGAGGGCGATGCCACCAACGGCAAGCTGACCCTGAAGTTCAT
CTGCACCACCGGCAAGCTGCCCGTGCCCTGGCCCACCCTCGTGACCAC
CCTGACCTACGGCGTGCAGTGCTTCAGCCGCTACCCCGACCACATGAA
GCAGCACGACTTCTTCAAGTCCGCCATGCCCGAAGGCTACGTCCAGGA
GCGCACCATCTCCTTCAAGGACGACGGCACCTACAAGACCCGCGCCGA
GGTGAAGTTCGAGGGCGACACCCTGGTGAACCGCATCGAGCTGAAGGG
CATCGACTTCAAGGAGGACGGCAACATCCTGGGGCACAAGCTGGAGTA

CAACTACAACAGCCACAACGTCTATATCACCGCTGACAAGCAGAAGAAC
GGCATCAAGGCCAACTTCAAGATCCGCCACAACATCGAGGACGGCAGC
GTGCAGCTCGCCGACCACTACCAGCAGAACACCCCCATCGGCGACGGC
CCCGTGCTGCTGCCCGACAACCACTACCTGAGCACCCAGTCCGCCCTG
AGCAAAGACCCCAACGAGAAGCGCGATCACATGGTCCTGCTGGAGTTC
GTGACCGCCGCCGGGATCACTCTCGGCATGGACGAGCTGTACAAGAAC
ATGATCACCAGCGCCCTGCACAGAGCCGCCGATTGGGCCAAGAGCGTG
TTCTCTTCTGCCGCCCTGGGCGACCCTAGAAGAACCGCCAGACTCGTGA
ATGTGGCCGCCCAGCTGGCCAAGTACAGCGGCAAGAGCATCACCATCA
GCAGCGAGGGCAGCAAAGCCGCCCAGGAAGGCGCCTACCGGTTCATCA
GAAACCCCAACGTGTCCGCCGAGGCCATCAGAAAGGCTGGCGCCATGC
AGACCGTGAAGCTGGCCCAGGAATTCCCCGAGCTGCTGGCCATCGAGG
ACACCACCAGCCTGAGCTACAGACACCAGGTGGCCGAGGAACTGGGCA
AGCTGGGCAGCATCCAGGACAAGAGCAGAGGGTGGTGGGTGCACAGC
GTGCTGCTGCTGGAAGCCACCACCTTCAGAACCGTGGGCCTGCTGCAC
CAGGAATGGTGGATGAGGCCCGACGATCCCGCCGACGCCGACGAGAAA
GAAAGCGGCAAATGGCTGGCCGCTGCCGCCACCAGCAGACTGAGAATG
GGCTCTATGATGAGCAACGTGATCGCCGTGTGCGACAGAGAGGCCGAC
ATCCACGCCTACCTGCAGGACAAACTGGCCCACAACGAGAGATTCGTCG
TGCGGAGCAAGCACCCCCGGAAGGATGTGGAAAGCGGCCTGTACCTGT
ACGACCACCTGAAGAACCAGCCCGAGCTGGGAGGCTACCAGATCAGCA
TCCCCCAGAAAGGCGTGGTGGACAAGCGGGGCAAGCGGAAGAACAGAC
CCGCCAGAAAAGCCAGCCTGTCCCTGCGGAGCGGCCGGATCACACTGA
AGCAGGGCAACATCACCCTGAACGCCGTGCTGGCCGAAGAGATCAACC
CCCCCAAGGGCGAGACACCCCTGAAGTGGCTGCTGCTGACAAGCGAGC
CCGTGGAATCTCTGGCTCAGGCCCTGAGAGTGATCGACATCTACACCCA
CAGATGGCGGATCGAGGAATTCCACAAGGCCTGGAAAACCGGCGCTGG
CGCCGAGCGGCAGAGAATGGAAGAACCCGACAACCTGGAACGGATGGT
GTCCATCCTGAGCTTCGTGGCCGTGCGGCTGCTGCAGCTGAGAGAGAG
CTTTACCCCTCCACAGGCCCTGCGGGCTCAGGGACTGCTGAAAGAAGC
CGAGCACGTGGAAAGCCAGAGCGCCGAGACAGTGCTGACCCCCGATGA
GTGTCAGCTGCTGGGCTACCTGGACAAGGGCAAACGCAAGCGCAAAGA
GAAGGCCGGCTCTCTGCAGTGGGCCTACATGGCCATTGCCCGGCTGGG
CGGATTCATGGACAGCAAGAGAACCGGAATCGCCTCCTGGGGAGCCCT
GTGGGAGGGATGGGAAGCCCTGCAGTCTAAGCTGGACGGCTTCCTGGC
CGCCAAGGACCTGATGGCCCAGGGCATTAAGATCGGGCCCAGCAGAGG
CGGCGGATCTGAAGGGGGAGGATCTGAGGGCGGAAGCGGCACTAGTC
AATTGATGATCACCTCTGCCCTGCATCGGGCCGCTGACTGGGCTAAGTC
CGTGTTTAGCAGTGCCGCTCTGGGAGATCCCAGGCGGACAGCTAGACT
CGTGAACGTGGCAGCTCAGCTGGCTAAATACTCCGGCAAGTCTATCACC
ATCTCCTCCGAGGGCTCCAAGGCTGCTCAGGAAGGGGCTTACAGATTCA
TCCGGAATCCTAATGTGTCTGCCGAAGCCATTCGGAAGGCAGGGGCTAT
GCAGACAGTGAAACTGGCTCAGGAATTTCCAGAACTGCTGGCTATTGAA
GATACCACCTCCCTGTCCTACCGGCATCAGGTGGCAGAAGAACTGGGAA
AACTGGGCTCTATCCAGGATAAGTCCCGGGGGTGGTGGGTGCACTCTG
TGCTGCTGCTGGAAGCTACAACATTTCGGACAGTGGGACTGCTGCATCA
GGAATGGTGGATGCGCCCCGATGACCCTGCTGATGCAGATGAGAAAGA
ATCTGGAAAGTGGCTGGCAGCCGCCGCTACCTCCCGGCTGCGGATGGG
GAGCATGATGTCCAATGTGATTGCTGTGTGCGATCGGGAAGCCGATATT
CATGCTTATCTGCAGGATAAGCTGGCTCATAATGAGCGCTTCGTCGTGC

GCTCCAAGCACCCTAGAAAGGACGTGGAATCCGGGCTGTATCTGTATGA
TCATCTGAAAAATCAGCCTGAACTGGGCGGGTATCAGATCTCTATTCCTC
AGAAAGGGGTGGTGGATAAGAGAGGGAAGCGCAAGAATCGGCCTGCCC
GGAAAGCCTCTCTGAGCCTGAGAAGCGGCAGAATTACCCTGAAACAGG
GGAATATTACACTGAACGCTGTGCTGGCTGAGGAAATCAATCCTCCTAA
GGGCGAAACCCCTCTGAAATGGCTGCTGCTGACCTCCGAGCCTGTGGA
AAGCCTGGCCCAGGCACTGCGCGTGATCGATATCTATACACATCGGTGG
CGGATTGAAGAGTTTCACAAAGCTTGGAAAACAGGGGCAGGCGCTGAGA
GACAGCGGATGGAAGAACCTGATAATCTGGAAAGAATGGTGTCTATTCT
GTCCTTTGTGGCTGTGCGCCTGCTGCAGCTGCGCGAGTCTTTTACACCA
CCCCAGGCTCTGAGAGCCCAGGGCCTGCTGAAAGAGGCTGAACATGTG
GAATCCCAGTCCGCCGAAACCGTGCTGACACCTGACGAATGCCAGCTG
CTGGGATATCTGGATAAGGGGAAGCGGAAACGGAAAGAGAAAGCAGGC
AGCCTGCAGTGGGCTTATATGGCTATCGCCAGACTGGGGGGCTTTATGG
ACTCCAAGCGGACCGGCATTGCCTCTTGGGGCGCTCTGTGGGAAGGCT
GGGAGGCTCTGCAGTCCAAACTGGATGGATTTCTGGCCGCTAAAGATCT
GATGGCTCAGGGGATCAAAATCCATCATCACCATCACCACCTCTAGAGG
GCCCGTTTAAACCCGCTGATCAGCCTCGA

pRC2164
CTGTGCCTTCTAGTTGCCAGCCATCTGTTGTTTGCCCCTCCCCCGTGCC
TTCCTTGACCCTGGAAGGTGCCACTCCCACTGTCCTTTCCTAATAAAATG
AGGAAATTGCATCGCATTGTCTGAGTAGGTGTCATTCTATTCTGGGGGGT
GGGGTGGGGCAGGACAGCAAGGGGGAGGATTGGGAAGACAATAGCAG
GCATGCTGGGGATGCGGTGGGCTCTATGGCTTCTGAGGCGGAAAGAAC
CAGCTGGGGCTCTAGGGGGTATCCCCACGCGCCCTGTAGCGGCGCATT
AAGCGCGGCGGGTGTGGTGGTTACGCGCAGCGTGACCGCTACACTTGC
CAGCGCCCTAGCGCCCGCTCCTTTCGCTTTCTTCCCTTCCTTTCTCGCCA
CGTTCGCCGGCTTTCCCCGTCAAGCTCTAAATCGGGGCATCCCTTTAGG
GTTCCGATTTAGTGCTTTACGGCACCTCGACCCCAAAAAACTTGATTAGG
GTGATGGTTCACGTAGTGGGCCATCGCCCTGATAGACGGTTTTTCGCCC
TTTGACGTTGGAGTCCACGTTCTTTAATAGTGGACTCTTGTTCCAAACTG
GAACAACACTCAACCCTATCTCGGTCTATTCTTTTGATTTATAAGGGATTT
TGGGGATTTCGGCCTATTGGTTAAAAAATGAGCTGATTTAACAAAAATTTA
ACGCGAATTAATTCTGTGGAATGTGTGTCAGTTAGGGTGTGGAAAGTCC
CCAGGCTCCCCAGGCAGGCAGAAGTATGCAAAGCATGCATCTCAATTAG
TCAGCAACCAGGTGTGGAAAGTCCCCAGGCTCCCCAGCAGGCAGAAGT
ATGCAAAGCATGCATCTCAATTAGTCAGCAACCATAGTCCCGCCCCTAAC
TCCGCCCATCCCGCCCCTAACTCCGCCCAGTTCCGCCCATTCTCCGCCC
CATGGCTGACTAATTTTTTTTATTTATGCAGAGGCCGAGGCCGCCTCTGC
CTCTGAGCTATTCCAGAAGTAGTGAGGAGGCTTTTTTGGAGGCCTAGGC
TTTTGCAAAAAGCTCCCGGGAGCTTGTATATCCATTTTCGGATCTGATCA
GCACGTGTTGACAATTAATCATCGGCATAGTATATCGGCATAGTATAATA
CGACAAGGTGAGGAACTAAACCATGGCCAAGTTGACCAGTGCCGTTCCG
GTGCTCACCGCGCGCGACGTCGCCGGAGCGGTCGAGTTCTGGACCGAC
CGGCTCGGGTTCTCCCGGGACTTCGTGGAGGACGACTTCGCCGGTGTG
GTCCGGGACGACGTGACCCTGTTCATCAGCGCGGTCCAGGACCAGGTG
GTGCCGGACAACACCCTGGCCTGGGTGTGGGTGCGCGGCCTGGACGA
GCTGTACGCCGAGTGGTCGGAGGTCGTGTCCACGAACTTCCGGGACGC
CTCCGGGCCGGCCATGACCGAGATCGGCGAGCAGCCGTGGGGGCGGG

AGTTCGCCCTGCGCGACCCGGCCGGCAACTGCGTGCACTTCGTGGCCG
AGGAGCAGGACTGACACGTGCTACGAGATTTCGATTCCACCGCCGCCTT
CTATGAAAGGTTGGGCTTCGGAATCGTTTTCCGGGACGCCGGCTGGATG
ATCCTCCAGCGCGGGGATCTCATGCTGGAGTTCTTCGCCCACCCCAACT
TGTTTATTGCAGCTTATAATGGTTACAAATAAAGCAATAGCATCACAAATT
TCACAAATAAAGCATTTTTTTCACTGCATTCTAGTTGTGGTTTGTCCAAAC
TCATCAATGTATCTTATCATGTCTGTATACCGTCGACCTCTAGCTAGAGC
TTGGCGTAATCATGGTCATAGCTGTTTCCTGTGTGAAATTGTTATCCGCT
CACAATTCCACACAACATACGAGCCGGAAGCATAAAGTGTAAAGCCTGG
GGTGCCTAATGAGTGAGCTAACTCACATTAATTGCGTTGCGCTCACTGC
CCGCTTTCCAGTCGGGAAACCTGTCGTGCCAGCTGCATTAATGAATCGG
CCAACGCGCGGGGAGAGGCGGTTTGCGTATTGGGCGCTCTTCCGCTTC
CTCGCTCACTGACTCGCTGCGCTCGGTCGTTCGGCTGCGGCGAGCGGT
ATCAGCTCACTCAAAGGCGGTAATACGGTTATCCACAGAATCAGGGGAT
AACGCAGGAAAGAACATGTGAGCAAAAGGCCAGCAAAAGGCCAGGAAC
CGTAAAAAGGCCGCGTTGCTGGCGTTTTTCCATAGGCTCCGCCCCCCTG
ACGAGCATCACAAAAATCGACGCTCAAGTCAGAGGTGGCGAAACCCGAC
AGGACTATAAAGATACCAGGCGTTTCCCCCTGGAAGCTCCCTCGTGCGC
TCTCCTGTTCCGACCCTGCCGCTTACCGGATACCTGTCCGCCTTTCTCC
CTTCGGGAAGCGTGGCGCTTTCTCAATGCTCACGCTGTAGGTATCTCAG
TTCGGTGTAGGTCGTTCGCTCCAAGCTGGGCTGTGTGCACGAACCCCCC
GTTCAGCCCGACCGCTGCGCCTTATCCGGTAACTATCGTCTTGAGTCCA
ACCCGGTAAGACACGACTTATCGCCACTGGCAGCAGCCACTGGTAACAG
GATTAGCAGAGCGAGGTATGTAGGCGGTGCTACAGAGTTCTTGAAGTGG
TGGCCTAACTACGGCTACACTAGAAGGACAGTATTTGGTATCTGCGCTCT
GCTGAAGCCAGTTACCTTCGGAAAAAGAGTTGGTAGCTCTTGATCCGGC
AAACAAACCACCGCTGGTAGCGGTGGTTTTTTTGTTTGCAAGCAGCAGAT
TACGCGCAGAAAAAAAGGATCTCAAGAAGATCCTTTGATCTTTTCTACGG
GGTCTGACGCTCAGTGGAACGAAAACTCACGTTAAGGGATTTTGGTCAT
GAGATTATCAAAAAGGATCTTCACCTAGATCCTTTTAAATTAAAAATGAAG
TTTTAAATCAATCTAAAGTATATATGAGTAAACTTGGTCTGACAGTTACCA
ATGCTTAATCAGTGAGGCACCTATCTCAGCGATCTGTCTATTTCGTTCAT
CCATAGTTGCCTGACTCCCCGTCGTGTAGATAACTACGATACGGGAGGG
CTTACCATCTGGCCCCAGTGCTGCAATGATACCGCGAGACCCACGCTCA
CCGGCTCCAGATTTATCAGCAATAAACCAGCCAGCCGGAAGGGCCGAG
CGCAGAAGTGGTCCTGCAACTTTATCCGCCTCCATCCAGTCTATTAATTG
TTGCCGGGAAGCTAGAGTAAGTAGTTCGCCAGTTAATAGTTTGCGCAAC
GTTGTTGCCATTGCTACAGGCATCGTGGTGTCACGCTCGTCGTTTGGTA
TGGCTTCATTCAGCTCCGGTTCCCAACGATCAAGGCGAGTTACATGATC
CCCCATGTTGTGCAAAAAAGCGGTTAGCTCCTTCGGTCCTCCGATCGTT
GTCAGAAGTAAGTTGGCCGCAGTGTTATCACTCATGGTTATGGCAGCAC
TGCATAATTCTCTTACTGTCATGCCATCCGTAAGATGCTTTTCTGTGACTG
GTGAGTACTCAACCAAGTCATTCTGAGAATAGTGTATGCGGCGACCGAG
TTGCTCTTGCCCGGCGTCAATACGGGATAATACCGCGCCACATAGCAGA
ACTTTAAAAGTGCTCATCATTGGAAAACGTTCTTCGGGGCGAAAACTCTC
AAGGATCTTACCGCTGTTGAGATCCAGTTCGATGTAACCCACTCGTGCA
CCCAACTGATCTTCAGCATCTTTTACTTTCACCAGCGTTTCTGGGTGAGC
AAAAACAGGAAGGCAAAATGCCGCAAAAAAGGGAATAAGGGCGACACG
GAAATGTTGAATACTCATACTCTTCCTTTTTCAATATTATTGAAGCATTTAT
CAGGGTTATTGTCTCATGAGCGGATACATATTTGAATGTATTTAGAAAAAT

AAACAAATAGGGGTTCCGCGCACATTTCCCCGAAAAGTGCCACCTGACG
TCGACGGATCGGGAGATCTCCCGATCCCCTATGGTCGACTCTCAGTACA
ATCTGCTCTGATGCCGCATAGTTAAGCCAGTATCTGCTCCCTGCTTGTGT
GTTGGAGGTCGCTGAGTAGTGCGCGAGCAAAATTTAAGCTACAACAAGG
CAAGGCTTGACCGACAATTGCATGAAGAATCTGCTTAGGGTTAGGCGTT
TTGCGCTGCTTCGCGATGTACGGGCCAGATATACGCGTTGACATTGATT
ATTGACTAGTTATTAATAGTAATCAATTACGGGGTCATTAGTTCATAGCCC
ATATATGGAGTTCCGCGTTACATAACTTACGGTAAATGGCCCGCCTGGCT
GACCGCCCAACGACCCCCGCCCATTGACGTCAATAATGACGTATGTTCC
CATAGTAACGCCAATAGGGACTTTCCATTGACGTCAATGGGTGGACTATT
TACGGTAAACTGCCCACTTGGCAGTACATCAAGTGTATCATATGCCAAGT
ACGCCCCCTATTGACGTCAATGACGGTAAATGGCCCGCCTGGCATTATG
CCCAGTACATGACCTTATGGGACTTTCCTACTTGGCAGTACATCTACGTA
TTAGTCATCGCTATTACCATGGTGATGCGGTTTTGGCAGTACATCAATGG
GCGTGGATAGCGGTTTGACTCACGGGGATTTCCAAGTCTCCACCCCATT
GACGTCAATGGGAGTTTGTTTTGGCACCAAAATCAACGGGACTTTCCAAA
ATGTCGTAACAACTCCGCCCCATTGACGCAAATGGGCGGTAGGCGTGTA
CGGTGGGAGGTCTATATAAGCAGAGCTCTCTGGCTAACTAGAGAACCCA
CTGCTTACTGGCTTATCGAAATTAATACGACTCACTATAGGGAGACCCAA
GCTGGCTAGCACCATGGAGCCCCCCAAGAAAAAGCGGAAAGTGCGGGT
TATGGTGAGCAAGGGCGAGGAGCTGTTCACCGGGGTGGTGCCCATCCT
GGTCGAGCTGGACGGCGACGTAAACGGCCACAAGTTCAGCGTGCGCGG
CGAGGGCGAGGGCGATGCCACCAACGGCAAGCTGACCCTGAAGTTCAT
CTGCACCACCGGCAAGCTGCCCGTGCCCTGGCCCACCCTCGTGACCAC
CCTGACCTACGGCGTGCAGTGCTTCAGCCGCTACCCCGACCACATGAA
GCAGCACGACTTCTTCAAGTCCGCCATGCCCGAAGGCTACGTCCAGGA
GCGCACCATCTCCTTCAAGGACGACGGCACCTACAAGACCCGCGCCGA
GGTGAAGTTCGAGGGCGACACCCTGGTGAACCGCATCGAGCTGAAGGG
CATCGACTTCAAGGAGGACGGCAACATCCTGGGGCACAAGCTGGAGTA
CAACTACAACAGCCACAACGTCTATATCACCGCTGACAAGCAGAAGAAC
GGCATCAAGGCCAACTTCAAGATCCGCCACAACATCGAGGACGGCAGC
GTGCAGCTCGCCGACCACTACCAGCAGAACACCCCCATCGGCGACGGC
CCCGTGCTGCTGCCCGACAACCACTACCTGAGCACCCAGTCCGCCCTG
AGCAAAGACCCCAACGAGAAGCGCGATCACATGGTCCTGCTGGAGTTC
GTGACCGCCGCCGGGATCACTCTCGGCATGGACGAGCTGTACAAGAAC
ATGATCACCAGCGCCCTGCACAGAGCCGCCGATTGGGCCAAGAGCGTG
TTCTCTTCTGCCGCCCTGGGCGACCCTAGAAGAACCGCCAGACTCGTGA
ATGTGGCCGCCCAGCTGGCCAAGTACAGCGGCAAGAGCATCACCATCA
GCAGCGAGGGCAGCAAAGCCGCCCAGGAAGGCGCCTACCGGTTCATCA
GAAACCCCAACGTGTCCGCCGAGGCCATCAGAAAGGCTGGCGCCATGC
AGACCGTGAAGCTGGCCCAGGAATTCCCCGAGCTGCTGGCCATCGAGG
ACACCACCAGCCTGAGCTACAGACACCAGGTGGCCGAGGAACTGGGCA
AGCTGGGCAGCATCCAGGACAAGAGCAGAGGGTGGTGGGTGCACAGC
GTGCTGCTGCTGGAAGCCACCACCTTCAGAACCGTGGGCCTGCTGCAC
CAGGAATGGTGGATGAGGCCCGACGATCCCGCCGACGCCGACGAGAAA
GAAAGCGGCAAATGGCTGGCCGCTGCCGCCACCAGCAGACTGAGAATG
GGCTCTATGATGAGCAACGTGATCGCCGTGTGCGACAGAGAGGCCGAC
ATCCACGCCTACCTGCAGGACAAACTGGCCCACAACGAGAGATTCGTCG
TGCGGAGCAAGCACCCCCGGAAGGATGTGGAAAGCGGCCTGTACCTGT
ACGACCACCTGAAGAACCAGCCCGAGCTGGGAGGCTACCAGATCAGCA

TCCCCCAGAAAGGCGTGGTGGACAAGCGGGGCAAGCGGAAGAACAGAC
CCGCCAGAAAAGCCAGCCTGTCCCTGCGGAGCGGCCGGATCACACTGA
AGCAGGGCAACATCACCCTGAACGCCGTGCTGGCCGAAGAGATCAACC
CCCCCAAGGGCGAGACACCCCTGAAGTGGCTGCTGCTGACAAGCGAGC
CCGTGGAATCTCTGGCTCAGGCCCTGAGAGTGATCGACATCTACACCCA
CAGATGGCGGATCGAGGAATTCCACAAGGCCTGGAAAACCGGCGCTGG
CGCCGAGCGGCAGAGAATGGAAGAACCCGACAACCTGGAACGGATGGT
GTCCATCCTGAGCTTCGTGGCCGTGCGGCTGCTGCAGCTGAGAGAGAG
CTTTACCCCTCCACAGGCCCTGCGGGCTCAGGGACTGCTGAAAGAAGC
CGAGCACGTGGAAAGCCAGAGCGCCGAGACAGTGCTGACCCCCGATGA
GTGTCAGCTGCTGGGCTACCTGGACAAGGGCAAACGCAAGCGCAAAGA
GAAGGCCGGCTCTCTGCAGTGGGCCTACATGGCCATTGCCCGGCTGGG
CGGATTCATGGACAGCAAGAGAACCGGAATCGCCTCCTGGGGAGCCCT
GTGGGAGGGATGGGAAGCCCTGCAGTCTAAGCTGGACGGCTTCCTGGC
CGCCAAGGACCTGATGGCCCAGGGCATTAAGATCCATCATCACCATCAC
CACCTCTAGAGGGCCCGTTTAAACCCGCTGATCAGCCTCGA

pRC2194
TCGCGCGTTTCGGTGATGACGGTGAAAACCTCTGACACATGCAGCTCCC
GGAGACGGTCACAGCTTGTCTGTAAGCGGATGCCGGGAGCAGACAAGC
CCGTCAGGGCGCGTCAGCGGGTGTTGGCGGGTGTCGGGGCTGGCTTA
ACTATGCGGCATCAGAGCAGATTGTACTGAGAGTGCACCATATGCGGTG
TGAAATACCGCACAGATGCGTAAGGAGAAAATACCGCATCAGGCGCCAT
TCGCCATTCAGGCTGCGCAACTGTTGGGAAGGGCGATCGGTGCGGGCC
TCTTCGCTATTACGCCAGCTGGCGAAAGGGGGATGTGCTGCAAGGCGAT
TAAGTTGGGTAACGCCAGGGTTTTCCCAGTCACGACGTTGTAAAACGAC
GGCCAGTGAATTCCTGTCTCTTATACACATCTTGCATTGTTGGAATTTGC
CGTTTGATATTGGAATACATTCTTAAATAAATGTGGTTATGTTATACATCA
TTTTAATGCGCATTTCTCGCTTTACGTTTTTTTGCTAATGACTTATTACTTG
CTGTTTATTTTATGTTTATTTTAGACTGGGCGTTTTTCCATAGGCTCCGCC
CCCCTGACGAGCATCACAAAAATCGACGCTCAAGTCAGAGGTGGCGAAA
CCCGACAGGACTATAAAGATACCAGGCGTTTCCCCCTGGAAGCTCCCTC
GTGCGCTCTCCTGTTCCGACCCTGCCGCTTACCGGATACCTGTCCGCCT
TTCTCCCTTCGGGAAGCGTGGCGCTTTCTCATAGCTCACGCTGTAGGTA
TCTCAGTTCGGTGTAGGTCGTTCGCTCCAAGCTGGGCTGTGTGCTCGAA
CCCCCCGTTCAGCCCGACCGCTGCGCCTTATCCGGTAACTATCGTCTTG
AGTCCAACCCGGTAAGACACGACTTATCGCCACTGGCAGCAGCCACTGG
TAACAGGATTAGCAGAGCGAGGTATGTAGGCGGTGCTACAGAGTTCTTG
AAGTGGTGGCCTAACTACGGCTACACTAGAAGGACAGTATTTGGTATCT
GCGCTCTGCTGAAGCCAGTTACCTTCGGAAAAAGAGTTGGTAGCTCTTG
ATCCGGCAAACAAACCACCGCTGGTAGCGGTGGTTTTTTTGTTTGCAAG
CAGCAGATTACGCGCAGAAAAAAAGGATCTCAAGAAGATCCTTTGATCTT
TTCTACGCCCGATCCAGACATGATAAGATACATTGATGAGTTTGGACAAA
CCACAACTAGAATGCAGTGAAAAAAATGCTTTATTTGTGAAATTTGTGATG
CTATTGCTTTATTTGTAACCATTATAAGCTGCAATAAACAAGTTGGGGTG
GGCGAAGAACTCCAGCATGAGATCCCCGCGCTGGAGGATCATCCAGCC
GGCGTCCCGGAAAACGATTCCGAAGCCCAACCTTTCATAGAAGGCGGC
GGTGGAATCGAAATCTCGTGATGGCAGGTTGGGCGTCGCTTGGTCGGT
CATTTCGAAGATCCCCTCAGAAGAACTCGTCAAGAAGGCGATAGAAGGC
GATGCGCTGCGAATCGGGAGCGGCGATACCGTAAAGCACGAGGAAGCG

GTCAGCCCATTCGCCGCCAAGCTCTTCAGCAATATCACGGGTAGCCAAC
GCTATGTCCTGATAGCGGTCCGCCACACCCAGCCGGCCACAGTCGATG
AATCCAGAAAAGCGGCCATTTTCCACCATGATATTCGGCAAGCAGGCAT
CGCCATGGGTCACGACGAGATCATCGCCGTCGGGCATGCGCGCCTTGA
GCCTGGCGAACAGTTCGGCTGGCGCGAGCCCCTGATGCTCTTCGTCCA
GATCATCCTGATCGACAAGACCGGCTTCCATCCGAGTACGTGCTCGCTC
GATGCGATGTTTCGCTTGGTGGTCGAATGGGCAGGTAGCCGGATCAAG
CGTATGCAGCCGCCGCATTGCATCAGCCATGATGGATACTTTCTCGGCA
GGAGCAAGGTGAGATGACAGGAGATCCTGCCCCGGCACTTCGCCCAAT
AGCAGCCAGTCCCTTCCCGCTTCAGTGACAACGTCGAGCACAGCTGCG
CAAGGAACGCCCGTCGTGGCCAGCCACGATAGCCGCGCTGCCTCGTCC
TGCAGTTCATTCAGGGCACCGGACAGGTCGGTCTTGACAAAAAGAACCG
GGCGCCCCTGCGCTGACAGCCGGAACACGGCGGCATCAGAGCAGCCG
ATTGTCTGTTGTGCCCAGTCATAGCCGAATAGCCTCTCCACCCAAGCGG
CCGGAGAACCTGCGTGCAATCCATCTTGTTCAATGGCCGATCCCATGGT
TTAGTTCCTCACCTTGTCGTATTATACTATGCCGATATACTATGCCGATGA
TTAATTGTCAACAGGCTGCAGGTCGAAAGGCCCGGAGATGAGGAAGAG
GAGAACAGCGCGGCAGACGTGCGCTTTTGAAGCGTGCAGAATGCCGGG
CCTCCGGAGGACCTTCGGGCGCCCGCCCCGCCCCTGAGCCCGCCCCT
GAGCCCGCCCCCGGACCCACCCCTTCCCAGCCTCTGAGCCCAGAAAGC
GAAGGAGCAAAGCTGCTATTGGCCGCTGCCCCAAAGGCCTACCCGCTT
CCATTGCTCAGCGGTGCTGTCCATCTGCACGAGACTAGTGAGACGTGCT
ACTTCCATTTGTCACGTCCTGCACGACGCGAGCTGCGGGGCGGGGGGG
AACTTCCTGACTAGGGGAGGAGTAGAAGGTGGCGCGAAGGGGCCACCA
AAGAACGGAGCCGGTTGGCGCCTACCGGTGGATGTGGAATGTGTGCGA
GGCCAGAGGCCACTTGTGTAGCGCCAAGTGCCCAGCGGGGCTGCTAAA
GCGCATGCTCCAGACTGCCTTGGGAAAAGCGCCTCCCCTACCCGGTAG
AATTTCGACGACCTGCCAATTGCCACAGAATTGGGGATCCTCTAGATAAA
GATGTGTTTGAGCCTAGTTATAATGATTTAAAATTCACGGTCCAGATGTG
TATAAGAGACAGAAGCTTGGCGTAATCATGGTCATAGCTGTTTCCTGTGT
GAAATTGTTATCCGCTCACAATTCCACACAACATACGAGCCGGAAGCATA
AAGTGTAAAGCCTGGGGTGCCTAATGAGTGAGCTAACTCACATTAATTGC
GTTGCGCTCACTGCCCGCTTTCCAGTCGGGAAACCTGTCGTGCCAGCTG
CATTAATGAATCGGCCAACGCGCGGGGAGAGGCGGTTTGCGTATTGGG
CGCTCTTCCGCTTCCTCGCTCACTGACTCGCTGCGCTCGGTCGTTCGGC
TGCGGCGAGCGGTATCAGCTCACTCAAAGGCGGTAATACGGTTATCCAC
AGAATCAGGGGATAACGCAGGAAAGAACATGTCCATAGAGCCCACCGCA
TCCCCAGCATGCCTGCTATTGTCTTCCCAATCCTCCCCCTTGCTGTCCTG
CCCCACCCCACCCCCCAGAATAGAATGACACCTACTCAGACAATGCGAT
GCAATTTCCTCATTTTATTAGGAAAGGACAGTGGGAGTGGCACCTTCCAG
GGTCAAGGAAGGCACGGGGGAGGGGCAAACAACAGATGGCTGGCAACT
AGAAGGCACAGTCGAGGCTGATCAGCGGGTTTAAACGGGCCCTCTAGAT
TAGTGGTGATGGTGATGATGGTTAGCCTCCCCCATCTCCCGGGCAAACG
TGCGCGCCAGGTCGCATATCGTCGGTATGGAGCCGGGGGTGGTGACGT
GGGTCTGGACCATCCCGGAGGTAAGTTGCAGCAGGGCGTCCCGGCAGC
CGGCGGGCGATTGGTCGTAATCCAGGATAAAGACGTGCATGGAACGGA
GGCGTTTGGCCAAGACGTCCAAGGCCCAGGCAAACACGTTATACAGGTC
GCCGTTGGGGGCCAGCAACTCGGGGGCCCGAAACAGGGTAAATAACGT
GTCCCCGATATGGGGTCGTGGGCCCGCGTTGCTCTGGGGCTCGGCACC
CTGGGGCGGCACGGCCGTCCCCGAAAGCTGTCCCCAGTCCTCCCGCCA

CGACCCGCCGCACTGCAGATACCGCACCGTATTGGCAAGTAGCCCGTA
AACGCGGCGAATCGCAGCCAGCATAGCCAGGTCCAGCCGCTCGCCGGG
GCGCTGGCGTTTGGCCAGGCGGTCGATGTGTCTGTCCTCCGGAAGGGC
CCCAAGCACGATGTTGGTGCCGGGCAAGGTCGGCGGGATGAGGGCCA
CGAACGCCAGCACGGCCTGGGGGGTCATGCTGCCCATAAGGTACCGCG
CGGCCGGGTAGCACAGGAGGGCGGCGATGGGATGGCGGTCGAAGATG
AGGGTGAGGGCCGGGGGCGGGGCATGTGAGCTCCCAGCCTCCCCCCC
GATATGAGGAGCCAGAACGGCGTCGGTCACGGCATAAGGCATGCCCAT
TGTTATCTGGGCGCTTGTCATTACCACCGCCGCGTCCCCGGCCGATATC
TCACCCTGGTCGAGGCGGTGTTGTGTGGTGTAGATGTTCGCGATTGTCT
CGGAAGCCCCCAGCACCCGCCAGTAAGTCATCGGCTCGGGTACGTAGA
CGATATCGTCGCGCGAACCCAGGGCCACCAGCAGTTGCGTGGTGGTGG
TTTTCCCCATCCCGTGGGGACCGTCTATATAAACCCGCAGTAGCGTGGG
CATTTTCTGCTCCGGGCGGACTTCCGTGGCTTCTTGCTGCCGGCGAGG
GCGCAACGCCGTACGTCGGTTGCTATGGCCGCGAGAACGCGCAGCCTG
GTCGAACGCAGACGCGACTTCTACACAGCCATCGGTCCAGACGGCCGC
GCTTCTGCGGGCGATTTGTGTACGCCCGACAGTCCCGGCTCCGGATCG
GACGATTGCGTCGCATCGACCCTGCGCCCAAGCTGCATCATCGAAATTG
CCGTCAACCAAGCTCTGATAGAGTTGGTCAAGACCAATGCGGAGCATAT
ACGCCCGGAGCCGCGGCGATCCTGCAAGCTCCGGATGCCTCCGCTCGA
AGTAGCGCGTCTGCTGCTCCATACAAGCCAACCACGGCCTCCAGAAGAA
GATGTTGGCGACCTCGTATTGGGAATCCCCGAACATCGCCTCGCTCCAG
TCAATGACCGCTGTTATGCGGCCATTGTCCGTCAGGACATTGTTGGAGC
CGAAATCCGCGTGCACGAGGTGCCGGACTTCGGGGCAGTCCTCGGCCC
AAAGCATCAGCTCATCGAGAGCCTGCGCGACGGACGCACTGACGGTGT
CGTCCATCACAGTTTGCCAGTGATACACATGGGGATCAGCAATCGCGCA
TATGAAATCACGCCATGTAGTGTATTGACCGATTCCTTGCGGTCCGAATG
GGCCGAACCCGCTCGTCTGGCTAAGATCGGCCGCAGCGATGGCATCCA
TGGCCTCCGCGACCGGCTGCAGAACAGCGGGCAGTTCGGTTTCAGGCA
GGTCTTGCAACGTGACACCCTGTGCACGGCGGGAGATGCAATAGGTCA
GGCTCTCGCTGAATTCCCCAATGTCAAGCACTTCCGGAATCGGGAGCGC
GGCCGATGCAAAGTGCCGATAAACATAACGATCTTTGTAGAAACCATCG
GCGCAGCTATTTACCCGCAGGACATATCCACGCCCTCCTACATCGAAGC
TGAAAGCACGAGATTCTTCGCCCTCCGAGAGCTGCATCAGGTCGGAAAC
GCTGTCGAACTTTTCGATCAGAAACTTCTCGACAGACGTCGCGGTGAGT
TCAGGCTTTTTCATGGTGGCCTCGAGCGGCCGCCACTGTGCTGGATATC
TGCAGAATTCCACCACACTGGACTAGTGGATCCGAGCTCGGTACCAAGC
TTAAGTTTAAACGCTAGCCAGCTTGGGTCTCCCTATAGTGAGTCGTATTA
ATTTCGATAAGCCAGTAAGCAGTGGGTTCTCTAGTTAGCCAGAGAGCTCT
GCTTATATAGACCTCCCACCGTACACGCCTACCGCCCATTTGCGTCAAT
GGGGCGGAGTTGTTACGACATTTTGGAAAGTCCCGTTGATTTTGGTGCC
AAAACAAACTCCCATTGACGTCAATGGGGTGGAGACTTGGAAATCCCCG
TGAGTCAAACCGCTATCCACGCCCATTGATGTACTGCCAAAACCGCATC
ACACATGTGAGCAAAAGGCCAGCAAAAGGCCAGGAACCGTAAAAAGGC
CGCGTTGCTGGCGTTTTTCCATAGGCTCCGCCCCCCTGACGAGCATCAC
AAAAATCGACGCTCAAGTCAGAGGTGGCGAAACCCGACAGGACTATAAA
GATACCAGGCGTTTCCCCCTGGAAGCTCCCTCGTGCGCTCTCCTGTTCC
GACCCTGCCGCTTACCGGATACCTGTCCGCCTTTCTCCCTTCGGGAAGC
GTGGCGCTTTCTCATAGCTCACGCTGTAGGTATCTCAGTTCGGTGTAGG
TCGTTCGCTCCAAGCTGGGCTGTGTGCACGAACCCCCCGTTCAGCCCG

ACCGCTGCGCCTTATCCGGTAACTATCGTCTTGAGTCCAACCCGGTAAG
ACACGACTTATCGCCACTGGCAGCAGCCACTGGTAACAGGATTAGCAGA
GCGAGGTATGTAGGCGGTGCTACAGAGTTCTTGAAGTGGTGGCCTAACT
ACGGCTACACTAGAAGGACAGTATTTGGTATCTGCGCTCTGCTGAAGCC
AGTTACCTTCGGAAAAAGAGTTGGTAGCTCTTGATCCGGCAAACAAACCA
CCGCTGGTAGCGGTGGTTTTTTTGTTTGCAAGCAGCAGATTACGCGCAG
AAAAAAAGGATCTCAAGAAGATCCTTTGATCTTTTCTACGGGGTCTGACG
CTCAGTGGAACGAAAACTCACGTTAAGGGATTTTGGTCATGAGATTATCA
AAAAGGATCTTCACCTAGATCCTTTTAAATTAAAAATGAAGTTTTAAATCA
ATCTAAAGTATATATGAGTAAACTTGGTCTGACAGTTACCAATGCTTAATC
AGTGAGGCACCTATCTCAGCGATCTGTCTATTTCGTTCATCCATAGTTGC
CTGACTCCCCGTCGTGTAGATAACTACGATACGGGAGGGCTTACCATCT
GGCCCCAGTGCTGCAATGATACCGCGAGACCCACGCTCACCGGCTCCA
GATTTATCAGCAATAAACCAGCCAGCCGGAAGGGCCGAGCGCAGAAGT
GGTCCTGCAACTTTATCCGCCTCCATCCAGTCTATTAATTGTTGCCGGGA
AGCTAGAGTAAGTAGTTCGCCAGTTAATAGTTTGCGCAACGTTGTTGCCA
TTGCTACAGGCATCGTGGTGTCACGCTCGTCGTTTGGTATGGCTTCATTC
AGCTCCGGTTCCCAACGATCAAGGCGAGTTACATGATCCCCCATGTTGT
GCAAAAAAGCGGTTAGCTCCTTCGGTCCTCCGATCGTTGTCAGAAGTAA
GTTGGCCGCAGTGTTATCACTCATGGTTATGGCAGCACTGCATAATTCTC
TTACTGTCATGCCATCCGTAAGATGCTTTTCTGTGACTGGTGAGTACTCA
ACCAAGTCATTCTGAGAATAGTGTATGCGGCGACCGAGTTGCTCTTGCC
CGGCGTCAATACGGGATAATACCGCGCCACATAGCAGAACTTTAAAAGT
GCTCATCATTGGAAAACGTTCTTCGGGGCGAAAACTCTCAAGGATCTTAC
CGCTGTTGAGATCCAGTTCGATGTAACCCACTCGTGCACCCAACTGATC
TTCAGCATCTTTTACTTTCACCAGCGTTTCTGGGTGAGCAAAAACAGGAA
GGCAAAATGCCGCAAAAAAGGGAATAAGGGCGACACGGAAATGTTGAAT
ACTCATACTCTTCCTTTTTCAATATTATTGAAGCATTTATCAGGGTTATTGT
CTCATGAGCGGATACATATTTGAATGTATTTAGAAAAATAAACAAATAGG
GGTTCCGCGCACATTTCCCCGAAAAGTGCCACCTGACGTCTAAGAAACC
ATTATTATCATGACATTAACCTATAAAAATAGGCGTATCACGAGGCCCTTT
CGTC

pRC1721
CCGACACCATCGAATGGTGCAAAACCTTTCGCGGTATGGCATGATAGCG
CCCGGAAGAGAGTCAATTCAGGGTGGTGAATGTGAAACCAGTAACGTTA
TACGATGTCGCAGAGTATGCCGGTGTCTCTTATCAGACCGTTTCCCGCG
TGGTGAACCAGGCCAGCCACGTTTCTGCGAAAACGCGGGAAAAAGTGG
AAGCGGCGATGGCGGAGCTGAATTACATTCCCAACCGCGTGGCACAAC
AACTGGCGGGCAAACAGTCGTTGCTGATTGGCGTTGCCACCTCCAGTCT
GGCCCTGCACGCGCCGTCGCAAATTGTCGCGGCGATTAAATCTCGCGC
CGATCAACTGGGTGCCAGCGTGGTGGTGTCGATGGTAGAACGAAGCGG
CGTCGAAGCCTGTAAAGCGGCGGTGCACAATCTTCTCGCGCAACGCGT
CAGTGGGCTGATCATTAACTATCCGCTGGATGACCAGGATGCCATTGCT
GTGGAAGCTGCCTGCACTAATGTTCCGGCGTTATTTCTTGATGTCTCTGA
CCAGACACCCATCAACAGTATTATTTTCTCCCATGAAGACGGTACGCGAC
TGGGCGTGGAGCATCTGGTCGCATTGGGTCACCAGCAAATCGCGCTGTT
AGCGGGCCCATTAAGTTCTGTCTCGGCGCGTCTGCGTCTGGCTGGCTG
GCATAAATATCTCACTCGCAATCAAATTCAGCCGATAGCGGAACGGGAA
GGCGACTGGAGTGCCATGTCCGGTTTTCAACAAACCATGCAAATGCTGA

ATGAGGGCATCGTTCCCACTGCGATGCTGGTTGCCAACGATCAGATGGC
GCTGGGCGCAATGCGCGCCATTACCGAGTCCGGGCTGCGCGTTGGTGC
GGATATCTCGGTAGTGGGATACGACGATACCGAAGACAGCTCATGTTAT
ATCCCGCCGTTAACCACCATCAAACAGGATTTTCGCCTGCTGGGGCAAA
CCAGCGTGGACCGCTTGCTGCAACTCTCTCAGGGCCAGGCGGTGAAGG
GCAATCAGCTGTTGCCCGTCTCACTGGTGAAAAGAAAAACCACCCTGGC
GCCCAATACGCAAACCGCCTCTCCCCGCGCGTTGGCCGATTCATTAATG
CAGCTGGCACGACAGGTTTCCCGACTGGAAAGCGGGCAGTGAGCGCAA
CGCAATTAATGTGAGTTAGCTCACTCATTAGGCACAATTCTCATGTTTGA
CAGCTTATCATCGACTGCACGGTGCACCAATGCTTCTGGCGTCAGGCAG
CCATCGGAAGCTGTGGTATGGCTGTGCAGGTCGTAAATCACTGCATAAT
TCGTGTCGCTCAAGGCGCACTCCCGTTCTGGATAATGTTTTTTGCGCCG
ACATCATAACGGTTCTGGCAAATATTCTGAAATGAGCTGTTGACAATTAAT
CATCGGCTCGTATAATGTGTGGAATTGTGAGCGGATAACAATTTCACACA
GGAAACAGCCAGTCCGTTTAGGTGTTTTCACGAGCACTTCACCAACAAG
GACCATAGCATATGGAAATGATGCTCGATAAGAAACAGATTCGTGCGATC
TTTCTCTTTGAGTTTAAAATGGGTCGCAAAGCGGCGGAGACGACGCGTA
ATATTAACAACGCGTTCGGTCCTGGCACCGCGAACGAGCGTACCGTGCA
ATGGTGGTTCAAAAAGTTTCGCAAAGGCGACGAATCTCTGGAGGACGAA
GAGCGTTCTGGCCGCCCGTCCGAGGTTGACAACGACCAGCTGCGTGCA
ATCATCGAAGCTGATCCGCTGACTACCACCCGCGAAGTTGCTGAAGAAC
TGAATGTGGATCACTCTACTGTGGTTCGCCACCTGAAACAGATCGGTAAA
GTAAAAAAACTGGACAAATGGGTTCCTCATGAACTGTCTGAAAACCAGAA
AAACCGTCGTTTCGAAGTTAGCTCCTCTCTGATTCTGCGTAACAACAACG
AACCGTTCCTGGATCGTATCGTAACCTGTGATGAGAAATGGATTCTGTAT
GATAACCGTCGCCGCTCTGCTCAGTGGCTGGATCGCGAAGAAGCTCCAA
AACACTTCCCGAAACCGAATCTGCACCAGAAGAAAGTCATGGTAACCGT
ATGGTGGTCTGCCGCAGGTGTTATCCACTATTCCTTCCTGAACCCGGGC
GAAACTATCACCAGCGAAAAATACTGCCAGCAGATTGACGAAATGCACC
GTAAACTGCAGCGTCTGCAGCCAGCACTGGTGAATCGTAAAGGTCCGAT
CCTGCTGCATGATAACGCCCGTCCGCACGTTGCCCAACCGACCCTGCA
GAAACTGAACGAACTGGGCTATGAAGTTCTGCCACACCCGCCGTACTCC
CCGGATCTGTCCCCGACTGACTACCATTTCTTCAAGCATCTGGACAACTT
CCTGCAGGGTAAACGTTTTCACAACCAACAGGACGCAGAAAACGCTTTC
CAGGAGTTCGTCGAAAGCCGTTCCACTGACTTCTACGCGACCGGTATCA
ACAAGCTGATCAGCCGTTGGCAGAAATGCGTGGACTGTAACGGCAGCTA
CTTCGATTAAGGATCCTCTAGAGTCGACCTGCAGGCAAGCTTGGCACTG
GCCGTCGTTTTACAACGTCGTGACTGGGAAAACCCTGGCGTTACCCAAC
TTAATCGCCTTGCAGCACATCCCCCTTTCGCCAGCTGGCGTAATAGCGA
AGAGGCCCGCACCGATCGCCCTTCCCAACAGTTGCGCAGCCTGAATGG
CGAATGGCAGCTTGGCTGTTTTGGCGGATGAGATAAGATTTTCAGCCTG
ATACAGATTAAATCAGAACGCAGAAGCGGTCTGATAAAACAGAATTTGCC
TGGCGGCAGTAGCGCGGTGGTCCCACCTGACCCCATGCCGAACTCAGA
AGTGAAACGCCGTAGCGCCGATGGTAGTGTGGGGTCTCCCCATGCGAG
AGTAGGGAACTGCCAGGCATCAAATAAAACGAAAGGCTCAGTCGAAAGA
CTGGGCCTTTCGTTTTATCTGTTGTTTGTCGGTGAACGCTCTCCTGAGTA
GGACAAATCCGCCGGGAGCGGATTTGAACGTTGCGAAGCAACGGCCCG
GAGGGTGGCGGGCAGGACGCCCGCCATAAACTGCCAGGCATCAAATTA
AGCAGAAGGCCATCCTGACGGATGGCCTTTTTGCGTTTCTACAAACTCTT
TTTGTTTATTTTTCTAAATACATTCAAATATGTATCCGCTCATGAGACAATA

ACCCTGATAAATGCTTCAATAATATTGAAAAAGGAAGAGTATGAGTATTCA
ACATTTCCGTGTCGCCCTTATTCCCTTTTTTGCGGCATTTTGCCTTCCTGT
TTTTGCTCACCCAGAAACGCTGGTGAAAGTAAAAGATGCTGAAGATCAGT
TGGGTGCACGAGTGGGTTACATCGAACTGGATCTCAACAGCGGTAAGAT
CCTTGAGAGTTTTCGCCCCGAAGAACGTTCTCCAATGATGAGCACTTTTA
AAGTTCTGCTATGTGGCGCGGTATTATCCCGTGTTGACGCCGGGCAAGA
GCAACTCGGTCGCCGCATACACTATTCTCAGAATGACTTGGTTGAGTACT
CACCAGTCACAGAAAAGCATCTTACGGATGGCATGACAGTAAGAGAATT
ATGCAGTGCTGCCATAACCATGAGTGATAACACTGCGGCCAACTTACTTC
TGACAACGATCGGAGGACCGAAGGAGCTAACCGCTTTTTTGCACAACAT
GGGGGATCATGTAACTCGCCTTGATCGTTGGGAACCGGAGCTGAATGAA
GCCATACCAAACGACGAGCGTGACACCACGATGCCTGTAGCAATGGCAA
CAACGTTGCGCAAACTATTAACTGGCGAACTACTTACTCTAGCTTCCCGG
CAACAATTAATAGACTGGATGGAGGCGGATAAAGTTGCAGGACCACTTC
TGCGCTCGGCCCTTCCGGCTGGCTGGTTTATTGCTGATAAATCTGGAGC
CGGTGAGCGTGGGTCTCGCGGTATCATTGCAGCACTGGGGCCAGATGG
TAAGCCCTCCCGTATCGTAGTTATCTACACGACGGGGAGTCAGGCAACT
ATGGATGAACGAAATAGACAGATCGCTGAGATAGGTGCCTCACTGATTA
AGCATTGGTAACTGTCAGACCAAGTTTACTCATATATACTTTAGATTGATT
TACCCCGGTTGATAATCAGAAAAGCCCCAAAAACAGGAAGATTGTATAAG
CAAATATTTAAATTGTAAACGTTAATATTTTGTTAAAATTCGCGTTAAATTT
TTGTTAAATCAGCTCATTTTTTAACCAATAGGCCGAAATCGGCAAAATCC
CTTATAAATCAAAAGAATAGACCGAGATAGGGTTGAGTGTTGTTCCAGTT
TGGAACAAGAGTCCACTATTAAAGAACGTGGACTCCAACGTCAAAGGGC
GAAAAACCGTCTATCAGGGCGATGGCCCACTACGTGAACCATCACCCAA
ATCAAGTTTTTTGGGGTCGAGGTGCCGTAAAGCACTAAATCGGAACCCT
AAAGGGAGCCCCCGATTTAGAGCTTGACGGGGAAAGCCGGCGAACGTG
GCGAGAAAGGAAGGGAAGAAAGCGAAAGGAGCGGGCGCTAGGGCGCT
GGCAAGTGTAGCGGTCACGCTGCGCGTAACCACCACACCCGCCGCGCT
TAATGCGCCGCTACAGGGCGCGTAAAAGGATCTAGGTGAAGATCCTTTT
TGATAATCTCATGACCAAAATCCCTTAACGTGAGTTTTCGTTCCACTGAG
CGTCAGACCCCGTAGAAAAGATCAAAGGATCTTCTTGAGATCCTTTTTTT
CTGCGCGTAATCTGCTGCTTGCAAACAAAAAAACCACCGCTACCAGCGG
TGGTTTGTTTGCCGGATCAAGAGCTACCAACTCTTTTTCCGAAGGTAACT
GGCTTCAGCAGAGCGCAGATACCAAATACTGTCCTTCTAGTGTAGCCGT
AGTTAGGCCACCACTTCAAGAACTCTGTAGCACCGCCTACATACCTCGC
TCTGCTAATCCTGTTACCAGTGGCTGCTGCCAGTGGCGATAAGTCGTGT
CTTACCGGGTTGGACTCAAGACGATAGTTACCGGATAAGGCGCAGCGGT
CGGGCTGAACGGGGGGTTCGTGCACACAGCCCAGCTTGGAGCGAACGA
CCTACACCGAACTGAGATACCTACAGCGTGAGCTATGAGAAAGCGCCAC
GCTTCCCGAAGGGAGAAAGGCGGACAGGTATCCGGTAAGCGGCAGGGT
CGGAACAGGAGAGCGCACGAGGGAGCTTCCAGGGGGAAACGCCTGGT
ATCTTTATAGTCCTGTCGGGTTTCGCCACCTCTGACTTGAGCGTCGATTT
TTGTGATGCTCGTCAGGGGGGCGGAGCCTATGGAAAAACGCCAGCAAC
GCGGCCTTTTTACGGTTCCTGGCCTTTTGCTGGCCTTTTGCTCACATGTT
CTTTCCTGCGTTATCCCCTGATTCTGTGGATAACCGTATTACCGCCTTTG
AGTGAGCTGATACCGCTCGCCGCAGCCGAACGACCGAGCGCAGCGAGT
CAGTGAGCGAGGAAGCGGAAGAGCGCCTGATGCGGTATTTTCTCCTTAC
GCATCTGTGCGGTATTTCACACCGCATATATGGTGCACTCTCAGTACAAT
CTGCTCTGATGCCGCATAGTTAAGCCAGTATACACTCCGCTATCGCTAC

GTGACTGGGTCATGGCTGCGCCCCGACACCCGCCAACACCCGCTGACG
CGCCCTGACGGGCTTGTCTGCTCCCGGCATCCGCTTACAGACAAGCTGT
GACCGTCTCCGGGAGCTGCATGTGTCAGAGGTTTTCACCGTCATCACCG
AAACGCGCGAGGCAGCTGCGGTAAAGCTCATCAGCGTGGTCGTGCAGC
GATTCACAGATGTCTGCCTGTTCATCCGCGTCCAGCTCGTTGAGTTTCTC
CAGAAGCGTTAATGTCTGGCTTCTGATAAAGCGGGCCATGTTAAGGGCG
GTTTTTTCCTGTTTGGTCACTTGATGCCTCCGTGTAAGGGGGAATTTCTG
TTCATGGGGGTAATGATACCGATGAAACGAGAGAGGATGCTCACGATAC
GGGTTACTGATGATGAACATGCCCGGTTACTGGAACGTTGTGAGGGTAA
ACAACTGGCGGTATGGATGCGGCGGGACCAGAGAAAAATCACTCAGGG
TCAATGCCAGCGCTTCGTTAATACAGATGTAGGTGTTCCACAGGGTAGC
CAGCAGCATCCTGCGATGCAGATCCGGAACATAATGGTGCAGGGCGCT
GACTTCCGCGTTTCCAGACTTTACGAAACACGGAAACCGAAGACCATTC
ATGTTGTTGCTCAGGTCGCAGACGTTTTGCAGCAGCAGTCGCTTCACGT
TCGCTCGCGTATCGGTGATTCATTCTGCTAACCAGTAAGGCAACCCCGC
CAGCCTAGCCGGGTCCTCAACGACAGGAGCACGATCATGCGCACCCGT
GGCCAGGACCCAACGCTGCCCGAAATT
